# Direct Synthesis of 5-Aryl Barbituric Acids by Rhodium(II)-Catalyzed Reactions of Arenes with Diazo Compounds**

**DOI:** 10.1002/anie.201502324

**Published:** 2015-05-08

**Authors:** Daniel Best, David J Burns, Hon Wai Lam

**Affiliations:** School of Chemistry, University of Nottingham, University Park Nottingham, NG7 2RD (UK)

**Keywords:** arylation, barbituric acid, carbenes, diazo compounds, rhodium

## Abstract

A commercially available rhodium(II) complex catalyzes the direct arylation of 5-diazobarbituric acids with arenes, allowing straightforward access to 5-aryl barbituric acids. Free N—H groups are tolerated on the barbituric acid, with no complications arising from N—H insertion processes. This method was applied to the concise synthesis of a potent matrix metalloproteinase (MMP) inhibitor.

Barbiturates have a long history in medicinal chemistry, having appeared in thousands of biologically active compounds since their emergence as sedatives and hypnotics at the turn of the 20th century.^1^ More than 100 years after its introduction, phenobarbital (Figure 1) remains the most widely prescribed antiepileptic drug worldwide.^2^ 5-Aryl barbituric acids have received renewed interest owing to their ability to inhibit matrix metalloproteinases (MMPs) and the tumor necrosis factor alpha (TNF-α) converting enzyme (TACE),^3^ leading to their application in cancer treatment^4^ and in vivo imaging^5^ (Figure 1).

**Figure 1:**
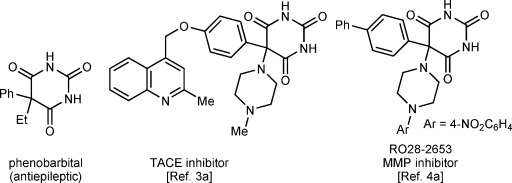
Biologically active 5-aryl barbituric acids.

Our interest in 5-aryl barbituric acids stems from their suitability as substrates for C—H functionalization; under ruthenium catalysis, they undergo oxidative annulation with alkynes to form spiroindenes.^6^ The conventional approach to 5-aryl barbituric acids is the condensation of ureas with 2-aryl malonic acids or esters3a, 4a,e, 5a, 7 (Scheme 1 a).^8^ In turn, 2-aryl malonic acids or esters can be prepared by palladium-5b, 9 or copper-catalyzed^10^ cross-couplings between malonates and haloarenes, or by alkoxycarbonylation of aryl acetate esters (which have limited commercial availability).4e, 5a, 7b

**scheme 1:**
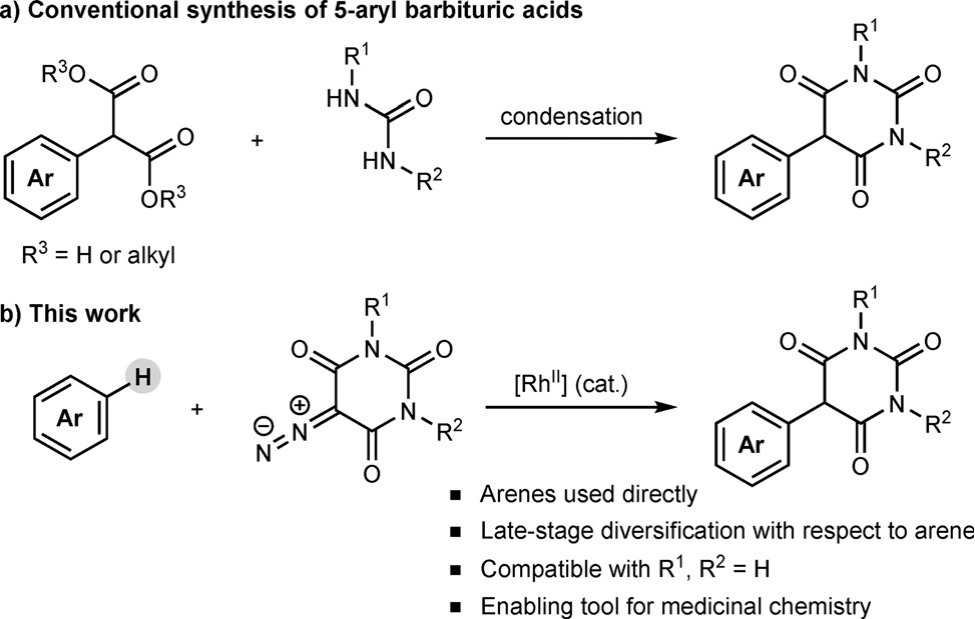
Synthesis of 5-aryl barbituric acids.

Although we found that these condensation routes to 5-aryl barbituric acids were sometimes successful, they were incompatible with electron-deficient aryl groups owing to decarboxylation and other problematic side reactions. Furthermore, this early-stage diversification strategy is not ideal for library synthesis. Our attempts to develop a late-stage diversification approach by adapting existing malonate–haloarene cross-couplings^9^, ^10^ to barbituric acids were unsuccessful because of poor reactivity. These limitations represent significant synthetic hurdles to compounds of considerable chemical and biological importance.

We envisioned an ideal strategy whereby a barbituric acid moiety would be coupled directly with arenes, without recourse to functional groups such as halides on the arene partner. As well as providing significantly improved access to useful substrates for C—H functionalization,^6^ a more direct approach to 5-aryl barbituric acids would be a highly enabling tool for medicinal chemists.^11^ Herein, we report the efficient Rh^II^-catalyzed direct arylation of 5-diazobarbituric acids with arenes at low catalyst loadings (Scheme 1 b) and its application to the concise synthesis of an MMP inhibitor.

Prior to our investigations, the direct arylation of α-diazocarbonyl compounds has shown promise.^12^ Whereas intramolecular C—H insertion reactions of α-diazocarbonyl compounds with arenes are well-known,^13^ intermolecular reactions are more challenging. The reaction of α-diazoesters or α-diazoketones with arenes under Rh^II^ catalysis results in cycloheptatrienes,^14^ which can undergo an acid-catalyzed rearrangement to give products of net α-arylation.^15^ More recent reports describe the arylation of α-diazoesters or closely related compounds with (hetero)arenes under metal^16^–^20^ or acid^21^ catalysis. However, the corresponding reactions of α-diazo-1,3-dicarbonyl compounds are less common,16d,e, 22, 23 and to the best of our knowledge, no reports of transition-metal-catalyzed couplings between 5-diazobarbituric acids and arenes exist.^24^ Indeed, despite the biological significance of barbiturates, catalytic transformations of 5-diazobarbituric acids appear to be restricted to the cyclopropanation of styrenes.^25^ Interestingly, these reactions were conducted in fluorobenzene, but no products resulting from arene C—H insertion were observed.^25^ Given these observations, the success of our proposed method was far from certain.

Fortunately, we discovered that just 0.1 mol % of commercially available [Rh_2_(esp)_2_]^26^ smoothly catalyzed the coupling of 5-diazo-1,3-dimethylbarbituric acid (**1 a**) with benzene at room temperature to give **2 a** in 77 % yield (Scheme 2).^27^ Further studies showed that a range of monosubstituted arenes were tolerated (**2 b**–**2 h**). These reactions were performed with no precautions to exclude air or water, and the inexpensive arenes were used as the solvent. The products were formed with moderate to excellent regioselectivities and, with the exception of **2 b** and **2 ka**, were isolated as mixtures of two regioisomers^28^ after chromatography. In most cases, recrystallization allowed for isolation of the pure *para* isomer (see the Supporting Information for details). Electron-rich arenes reacted with **1 a** to provide **2 b**–**2 d** in good yields. With toluene, no products from benzylic C—H insertion were observed.^27^ Use of a small excess of anisole (1.2 equiv) resulted in a reduced (but synthetically useful) 64 % yield of **2 c** owing to a lower conversion, attributable to inefficient mixing of the reagents. Fluorobenzene reacted smoothly to give **2 f** in 78 % yield, further demonstrating that relatively electron-neutral arenes (Hammett constant *σ*_p_ of F: 0.06)^29^ are effective. Chloro- and bromobenzene were also surprisingly effective (**2 g** and **2 h**), despite being deactivated substrates (*σ*_p_ of Cl and Br: 0.23).^29^ Even trifluoromethoxybenzene (*σ*_p_ of OCF_3_: 0.35)^29^ gave **2 e** in good yield, albeit in a 4:1 regioisomeric ratio. Arenes with *meta*-directing substituents, such as CF_3_, CN, CO_2_Me, or NO_2_ groups, were unsuitable, but disubstituted arenes, such as *meta*-xylene and 1,3-dimethoxybenzene, reacted with **1 a** to give **2 i** and **2 j** in high regioselectivities. The reactions of *ortho*-xylene and 1,2-dimethoxybenzene were high-yielding, but less regioselective (**2 ka** and **2 l**). With *ortho*-xylene, the minor regioisomer **2 kb** (see the Supporting Information for the structure) was also isolated in 14 % yield. 1-Methylindole reacted smoothly to give 5-(3-indolyl)barbituric acid **3** in 73 % yield [Eq. (1)].^16^

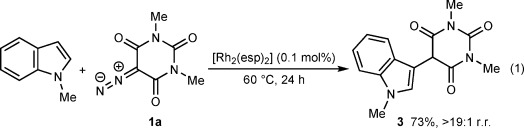
1

**scheme 2:**
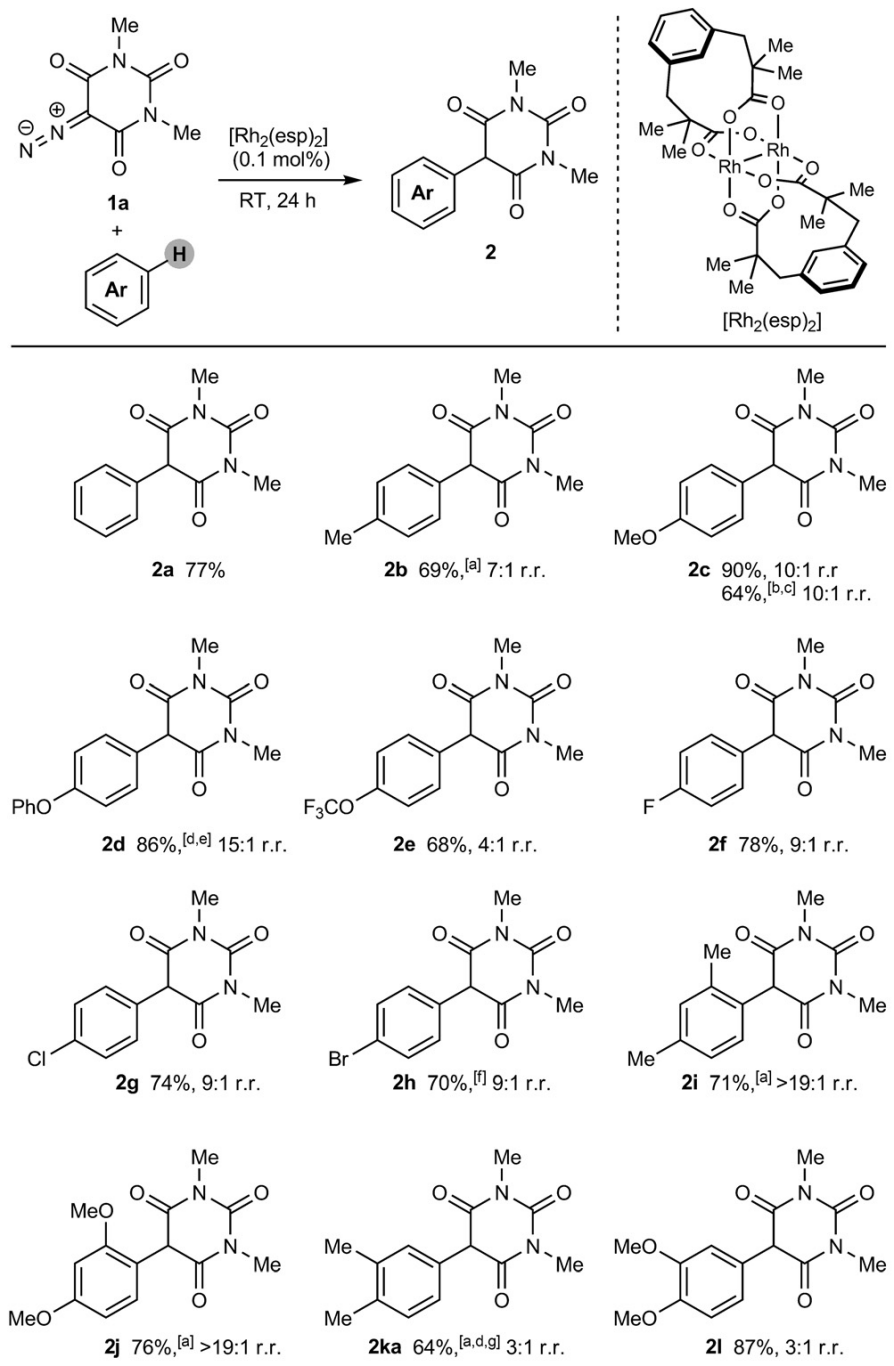
Rhodium(II)-catalyzed arylation of 5-diazobarbituric acid 1 a. Reactions were conducted with 2.00 mmol of 1 a in 2.0 mL of the arene. r.r.=regioisomeric ratio as determined by ^1^H NMR analysis of the unpurified reaction mixture. Yields are of isolated mixtures of inseparable regioisomers in the same ratio as in the unpurified mixtures. [a] Isolated as a single regioisomer. [b] Conducted with 3.00 mmol of 1 a and 3.60 mmol of anisole. [c] Isolated as a 15:1 mixture of regioisomers. [d] Conducted with 1.00 mmol of 1 a in 1.0 mL of the arene. [e] Conducted at 30 °C for 7 h. [f] Isolated as a 10:1 mixture of regioisomers. [g] The minor isomer 2 kb was isolated in 14 % yield.

Our focus now turned to the variation of the 5-diazobarbituric acid (Table 1). The first question to address was whether N alkylation is essential, given that most biologically active barbiturates are not 1,3-dialkylated, and free N—H groups might be expected to undergo insertion reactions with a rhodium carbenoid. Remarkably, this concern was unwarranted; 5-diazo-1-methylbarbituric acid **1 b** reacted with anisole to provide **4 a** in 68 % yield (entry 1), whereas 5-diazobarbituric acid **1 c**, which bears two free N—H groups, gave **4 b** in 93 % yield (entry 2). Coupling of **1 c** with diphenyl ether using 0.25 mol % of [Rh_2_(esp)_2_] at 120 °C gave **4 c** in excellent yield with good isomeric purity. Compound **4 c** is an important precursor to biologically active barbiturates.4b,e,f, 5b,c A thiocarbonyl group was also tolerated (entry 4), but our conditions did not provide good results when applied to other α-diazo-1,3-dicarbonyl compounds.^30^

**Table 1:** Rhodium(II)-catalyzed arylation of various 5-diazobarbituric acids.^[a]^ 
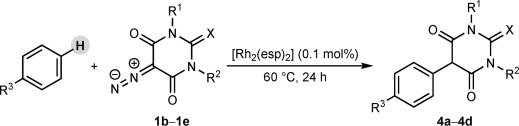

Entry	Product		*T* [°C]	Yield^[b]^ [%]	r.r.^[c]^
1	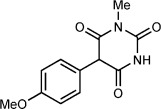	**4 a**	60	68^[d]^	10:1
2^[e]^	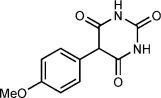	**4 b**	90	93	9:1
3^[f]^	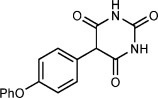	**4 c**	120	89	9:1
4	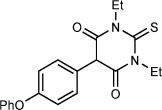	**4 d**	40	71	6:1

[a] Reactions were conducted with 0.50 mmol of **1 b**–**1 e** in 0.5 mL of the arene. [b] Yield of isolated inseparable mixtures of regioisomers in the same ratio as in the unpurified reaction mixtures. [c] Regioisomeric ratio as determined by ^1^H NMR analysis of the unpurified reaction mixtures. [d] Isolated as a single regioisomer. [e] Conducted with 1.00 mmol of **1 c** and 1.0 mL of anisole. [f] Conducted with 0.25 mol % of [Rh_2_(esp)_2_].

To further demonstrate the advantages of our method, we synthesized the potent and selective MMP inhibitor **8** (IC_50_: 1 nm vs. MMP-9 with 26-fold selectivity over MMP-2;5a Scheme 3). The Rh^II^-catalyzed reaction of diazobarbituric acid **1 c** with 4-bromophenyl phenyl ether occurred at the most sterically accessible site, with no evidence of reaction at the 4-bromophenyl ring, to give 5-aryl barbituric acid **5** in 87 % yield and a regioisomeric ratio of 9:1. This compound was previously accessed in six steps from commercial materials in 37 % overall yield,5a which highlights the brevity of our approach. Bromination at the C5 position with pyridinium tribromide provided the readily separable isomers **6** (51 %) and **7** (6 %).^31^ Displacement of the bromide of **6** with *N*-isopropylpiperazine then gave MMP inhibitor **8** in 70 % yield.5a

**scheme 3:**
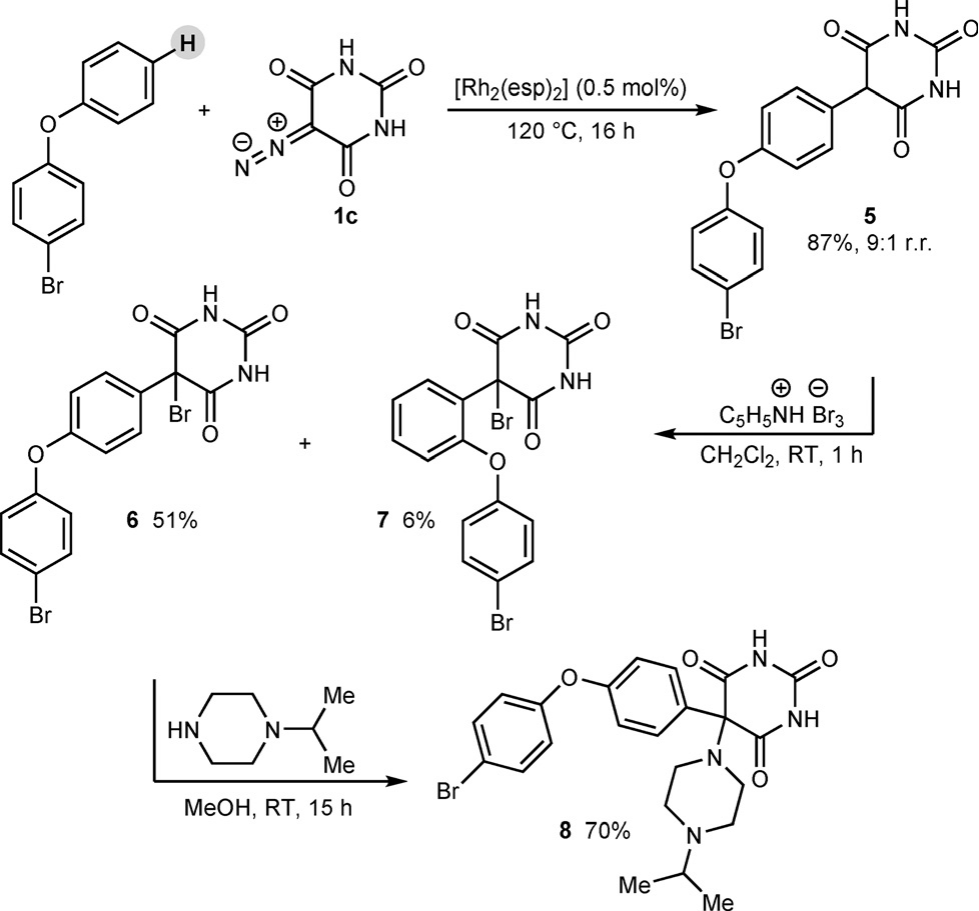
Synthesis of MMP-9 inhibitor 8.

Finally, [Rh_2_(esp)_2_] also efficiently catalyzes C(sp^3^)—H insertion reactions in the absence of arenes;^32^ alkylation of **1 a** with cyclohexane proceeded smoothly to form **9** in 83 % yield [Eq. (2)].

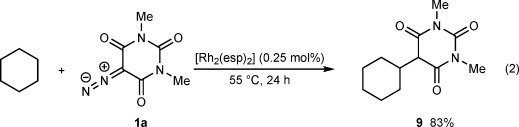
2

In conclusion, the coupling of arenes with 5-diazobarbituric acids proceeds efficiently under Rh^II^ catalysis to provide medicinally important compounds in a direct manner that is more suited to drug discovery than existing technologies. The method is compatible with free N—H groups on the barbituric acids, with no complications arising from N—H insertion processes. The operational simplicity, mild conditions, and low loading of a commercially available catalyst further increase the appeal of this method.

## Supporting Information

As a service to our authors and readers, this journal provides supporting information supplied by the authors. Such materials are peer reviewed and may be re-organized for online delivery, but are not copy-edited or typeset. Technical support issues arising from supporting information (other than missing files) should be addressed to the authors. miscellaneous_information
