# Supplementary material for: Direct Synthesis of 5-Aryl Barbituric Acids by Rhodium(II)-Catalyzed Reactions of Arenes with Diazo Compounds
Source: Angew Chem Int Ed Engl. 2015 May 8;54(25):7410–3. doi: 10.1002/anie.201502324 (PMC4479025; doi:10.1002/anie.201502324)
Supplement: Supplementary file 1 — As a service to our authors and readers, this journal provides supporting information supplied by the authors. Such materials are peer reviewed and may be re-organized for online delivery, but are not copy-edited or typeset. Technical support issues arising from supporting information (other than missing files) should be addressed to the authors. miscellaneous_information [file anie0054-7410-sd1.pdf]

Angewandte Chemie
Eine Zeitschrift der Gesellschaft Deutscher Chemiker

Supporting Information

Direct Synthesis of 5-Aryl Barbituric Acids by Rhodium(II)-Catalyzed Reactions of Arenes with Diazo Compounds**

*Daniel Best, David J. Burns, and Hon Wai Lam**

anie_201502324_sm_miscellaneous_information.pdf

Supporting Information

Contents **Page**

General Information 2

Preparation of 5-Diazobarbituric acids 2

Evaluation of Rhodium Complexes in the Arylation of 5-Diazo-1,3-dimethylbarbituric acid with Toluene 4

Rh(II)-Catalyzed Arylation of 5-Diazobarbituric acids 4

Synthesis of MMP inhibitor **8** 14

Rh(II)-Catalyzed Coupling of 5-diazo-1,3-dimethylbarbituric acid with Cyclohexane 15

NMR Spectra 16

General Information

Commercially available reagents and solvents were used as received. Pet. ether refers to 40–60 °C petroleum ether. IMS refers to Industrial Methylated Spirit (95:5 EtOH/MeOH). Thin layer chromatography (TLC) was performed on Merck DF-Alufoilien $60F_{254}$ 0.2 mm precoated plates. Compounds were visualized by exposure to UV light or by dipping the plates into solutions of potassium permanganate, ceric ammonium molybdate, or vanillin followed by heating. AcOH-doped silica TLC plates were prepared by soaking in *ca.* 20% AcOH/EtOAc followed by gentle drying with a heat gun. Flash column chromatography was carried out using silica gel (Sigma Aldrich 60 Å particle size 35–70 micron) with isocratic or step gradient elution as indicated. AcOH-doped silica columns were prepared by slurry packing in *ca.* 20% AcOH/PhMe followed by equilibration with 1% AcOH in the weak solvent component of the subsequently applied eluent (either PhMe or 1:1 PhMe/pet. ether). Melting points were recorded on a Gallenkamp melting point apparatus and are uncorrected. The solvent of recrystallization is reported in parentheses. Infra-red spectra were recorded on a Nicolet Avatar 360 FT instrument on the neat compound using an attenuated total reflection (ATR) accessory with a diamond crystal and a germanium sample plate. NMR spectra were acquired on a Jeol EX270, Bruker DPX300, Bruker AV400, Bruker AV(III)400, Bruker DPX400, or a Bruker AV(III)500 spectrometer. ^{1}H and ^{13}C NMR spectra were referenced to external tetramethylsilane, *via* the residual protonated solvent (^{1}H) or the solvent itself (^{13}C), or using $CFCl_3$ as an external standard at 0.00 ppm (^{19}F). All chemical shifts are reported in parts per million (ppm). For ^{1}H NMR spectroscopy, shifts are referenced to 7.27 ppm for $CDCl_3$, 2.50 ppm for $(CD_3)_2SO$, or 1.94 ppm for CD_3CN. For ^{13}C NMR spectroscopy, shifts are referenced to 77.0 ppm for $CDCl_3$, 39.50 ppm for $(CD_3)_2SO$, or 1.32 ppm for CD_3CN. Abbreviations used in the description of resonances are: s (singlet), d (doublet), t (triplet), q, (quartet), br (broad), m (multiplet). Coupling constants (*J*) are quoted to the nearest 0.1 Hz. Assignments in ^{13}C NMR spectroscopy were made using the DEPT sequence with secondary pulses at 90° and 135°. High resolution mass spectra were recorded using electrospray ionization (ESI) techniques at the School of Chemistry, University of Nottingham.

Preparation of 5-Diazobarbituric Acids

General Procedure A

To a mixture of the 1,3-dicarbonyl compound (1.0 equiv) and methanesulfonyl azide[1] (1.0–1.1 equiv) in MeCN at 0 °C was added Et_3N (2.0 equiv) dropwise. The ice bath was removed and the

1. Prepared according to: Matano, Y.; Ohkubo, H.; Honsho, Y.; Saito, A.; Seki, S.; Imahori, H. *Org. Lett.* **2013**, *15*, 932–935.

mixture stirred at room temperature for 2 h, pre-adsorbed onto silica gel and purified by flash column chromatography.

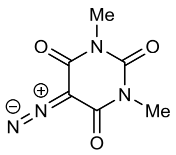

5-Diazo-1,3-dimethylbarbituric acid (1a).[2] The title compound was prepared according to General Procedure A from 1,3-dimethylbarbituric acid (7.80 g, 50 mmol), methanesulfonyl azide (6.29 g, 52 mmol), and Et_3N (14 mL, 100 mmol) in MeCN (100 mL), and purified by flash column chromatography (2–12% acetone/PhMe) to give a pale yellow solid (6.50 g, 71%). Physical and spectroscopic data were in accordance with the literature.[2]

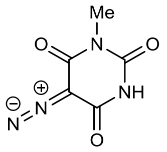

5-Diazo-1-methylbarbituric acid (1b).[3] The title compound was prepared according to General Procedure A from 1-methylbarbituric acid[4] (710 mg, 5.00 mmol), methanesulfonyl azide (666 mg, 5.50 mmol), and Et_3N (1.4 mL, 10 mmol) in MeCN (25 mL), and purified by flash column chromatography (5–30% acetone/PhMe) to give a white solid (616 mg, 73%). Physical and spectroscopic data were in accordance with the literature.[3]

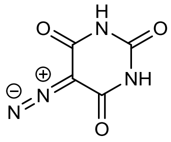

5-Diazobarbituric acid (1c).[2] General Procedure A was followed using barbituric acid (1.30 g, 10.0 mmol), methanesulfonyl azide (1.33 g, 11.0 mmol), and Et_3N (2.8 mL, 20 mmol) in MeCN (100 mL). The crude reaction mixture was passed through a short pad of silica gel, eluting with 7:2:1 EtOAc/IPA/H_2O, concentrated *in vacuo*, and recrystallized from IMS to provide the title compound (1.10 g, 71%) as a pale yellow crystalline solid. Physical and spectroscopic data were in accordance with the literature.[2]

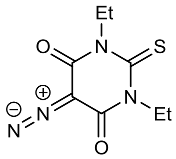

5-Diazo-1,3-diethyl-2-thiobarbituric acid (1d). The title compound was prepared according to General Procedure A from 1,3-diethyl-2-thiobarbituric acid (1.00 g, 5.00 mmol), methanesulfonyl azide (666 mg, 5.50 mmol), and Et_3N (1.4 mL, 10.0 mmol) in MeCN (25 mL), and purified by flash column chromatography (1–6% acetone/PhMe) to give a pale red solid (306 mg, 27%). R_f 0.75 (20% acetone/PhMe); m.p. 71–74 °C ($CHCl_3$); IR 2144, 1686, 1670, 1440, 1389, 1368, 1334, 1260, 1241, 1220, 1107, 1068, 877, 750 cm^{-1}; ^1H NMR (300 MHz, $(CD_3)_3SO$) δ 4.37 (4H, q, J = 7.0 Hz, 2 x C**H**$_2$), 1.18 (6H, t, J = 7.0 Hz, 2 x C**H**$_3$); ^{13}C NMR (75.5 MHz, $(CD_3)_3SO$) δ 177.8 (C), 156.4 (2 x C), 42.9 (2 x CH_2), 11.9 (2 x

2. Ramachary, D. B.; Narayana, V. V.; Ramakumar, K. *Tetrahedron Lett.* **2008**, *49*, 2704–2709.
3. Wessiak, A.; Bruice, T. C. *J. Am. Chem. Soc.* **1983**, *105*, 4809–4825.
4. Prepared according to: Mao, Y.; Tian, W.; Huang, Z.; An, J. *J. Heterocycl. Chem.* **2014**, *51*, 594–597.

CH_3), CN_2 not resolved; HRMS (ESI +ve) Exact mass calculated for $C_8H_{11}N_4O_2S$ $[M+H]^+$: 227.0597, found 227.0597.

Evaluation of Rhodium Complexes in the Arylation of 5-Diazo-1,3-dimethylbarbituric acid with Toluene[a]

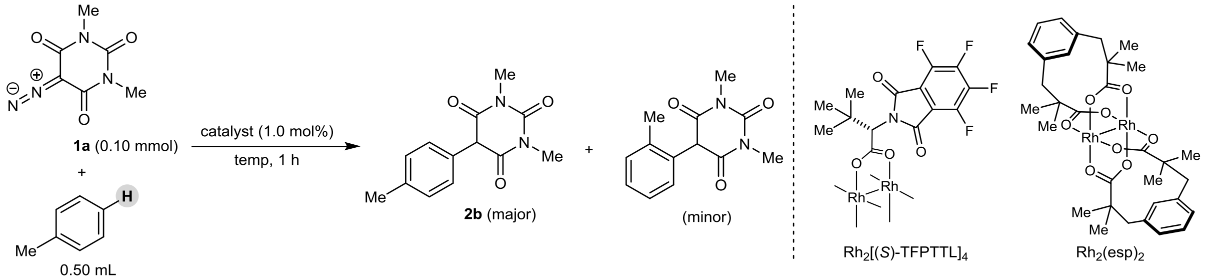

Entry	Catalyst	Temp [°C]	Yield [%][b]	r.r.[c]
1	$Rh_2(OAc)_4$	RT	0	–
2	$Rh_2(OAc)_4$	50	0	–
3	$Rh_2(O_2CCPh_3)_4$	RT	0	–
4	$Rh_2(O_2CCPh_3)_4$	50	0	–
5	$Rh_2[O_2C(n\text{-}C_7H_{15})]_4$	RT	0	–
6	$Rh_2(OPiv)_4$	RT	0	–
7[d]	$Rh_2(OPiv)_4$	50	51	88:12
8	$Rh_2[O_2C(n\text{-}C_3F_7)]_4$	RT	0	–
9	$Rh_2[(S)\text{-TFPTTL}]_4$	RT	64	92:8
10	$Rh_2(esp)_2$	RT	69	88:12

[a] Reactions were conducted with 0.10 mmol of **1a** in 0.50 mL of the toluene. [b] Yield of arylation products determined by ^{1}H NMR analysis of the unpurified reaction mixtures using 1,3,5-trimethoxybenzene as an internal standard. [c] Regioisomeric ratio as determined by ^{1}H NMR analysis of the unpurified reaction mixtures. [d] Reaction conducted for 1.5 h.

Despite the marginally superior performance of $Rh_2[(S)\text{-TFPTTL}]_4$, the more economical catalyst $Rh_2(esp)_2$ was chosen for further reaction development.

Rh(II)-Catalyzed Arylation of 5-Diazobarbituric Acids

General Procedure B

A slurry of 5-diazobarbituric acid **1a** (364 mg, 2.00 mmol) and $Rh_2(esp)_2$ (1.5 mg, 2.0 µmol) in the appropriate arene (2.00 mL) was stirred at room temperature for 24 h. The excess arene was removed *in vacuo* and the residue was purified by flash column chromatography to provide the 5-arylbarbituric acid, often as a mixture of regioisomers. Recrystallization from IMS/pet. ether provided a sample of the analytically pure *para*-isomer (recrystallization conditions were not optimized).

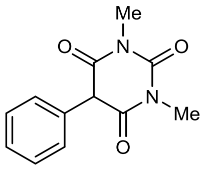

1,3-Dimethyl-5-phenylbarbituric acid (2a).[5] The title compound was prepared according to General Procedure B using benzene (2.00 mL). Purification by flash column chromatography (40:40:20:1 PhMe/pet. ether/acetone/AcOH on AcOH-doped silica gel) provided the title compound (356 mg, 77%) as a white crystalline solid. Physical and spectral data were in agreement with the literature.[5] R_f 0.53 (40:40:20:1 PhMe/pet. ether/acetone/AcOH on AcOH-doped SiO_2).

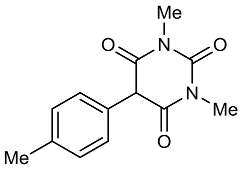

1,3-Dimethyl-5-(4-methylphenyl)barbituric acid (2b). General Procedure B was followed using toluene (2.00 mL). ^{1}H NMR analysis of the crude reaction mixture indicated a 7:1 mixture of regioisomers. Purification by flash column chromatography (40:40:20:1 PhMe/pet. ether/acetone/AcOH on AcOH-doped silica gel) gave the title compound (245 mg, 69%) as a single isomer as a white crystalline solid. R_f 0.60 (40:40:20:1 PhMe/pet. ether/acetone/AcOH on AcOH-doped SiO_2); m.p. 124–126 °C (IMS/pet. ether); IR 1698, 1668, 1455, 1439, 1423, 1388, 1371, 1287, 1256, 1133, 821, 762 cm^{-1}; ^{1}H NMR (400 MHz, $(CD_3)_2SO$) δ 7.15 (4H, apparent s, 4 × Ar**H**), 4.93 (1H, s, C**H**), 3.16 (6H, s, 2 x NC**H**$_3$), 2.29 (3H, s, ArC**H**$_3$); ^{13}C NMR (100.4 MHz, $(CD_3)_2SO$) δ 167.7 (2 x C), 151.8 (C), 137.2 (C), 131.7 (C), 129.2 (2 x CH), 129.0 (2 x CH), 54.9 (CH), 28.3 (2 x CH_3), 20.6 (CH_3); HRMS (ESI +ve) Exact mass calculated for $C_{13}H_{14}N_2NaO_3$ $[M+Na]^+$: 269.0897, found 269.0895.

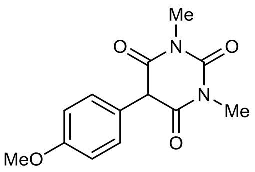

5-(4-Methoxyphenyl)-1,3-dimethylbarbituric acid (2c).[5] *Method 1:* General Procedure B was followed using anisole (2.00 mL). ^{1}H NMR analysis of the crude reaction mixture indicated a 10:1 mixture of regioisomers. Purification by flash column chromatography (40:40:20:1 PhMe/pet. ether/acetone/AcOH on AcOH-doped silica gel) provided the title compound (470 mg, 90%) as a 10:1 mixture of regioisomers as a white crystalline solid. The pure *para*-isomer was obtained by recrystallization from IMS/pet. ether (365 mg, 70%). Physical and spectroscopic data were in accordance with the literature.[5] R_f 0.50 (40:40:20:1 PhMe/pet. ether/acetone/AcOH on AcOH-doped SiO_2).

Method 2: A suspension of 5-diazobarbituric acid **1a** (546 mg, 3.00 mmol) and $Rh_2(esp)_2$ (2.3 mg, 3.0 µmol) in anisole (0.40 mL, 3.7 mmol) was stirred at room temperature for 24 h. Purification by flash column chromatography (4–20% acetone/(1:1 PhMe/pet. ether); 1% AcOH on AcOH-doped silica gel) provided the title compound (503 mg, 64%) as a 15:1 mixture of regioisomers as a white crystalline solid.

5. Reddy Chidipudi, S.; Khan, I.; Lam, H. W. *Angew. Chem., Int. Ed.* **2012**, *51*, 12115–12119.

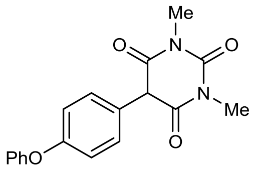

1,3-Dimethyl-5-(4-phenoxyphenyl)barbituric acid (2d). A modification of General Procedure B (in that the reaction scale was 1.00 mmol, the temperature was 30 °C and the reaction time was 7 h) was followed using 5-diazobarbituric acid **1a** (182 mg, 1.00 mmol), $Rh_2(esp)_2$ (0.8 mg, 1 µmol), and diphenyl ether (1.00 mL). ^{1}H NMR analysis of the crude reaction mixture indicated a 15:1 mixture of regioisomers. Purification by flash column chromatography (4–20% acetone/(1:1 PhMe/pet. ether); 1% AcOH on AcOH-doped silica gel) provided the title compound (280 mg, 86%) as a 15:1 mixture of regioisomers as a white crystalline solid. The pure *para*-isomer was obtained by recrystallization from IMS/pet. ether (199 mg, 61%). R_f 0.60 (40:40:20:1 PhMe/pet. ether/acetone/AcOH on AcOH-doped SiO_2); m.p. 147–148 °C (IMS/pet. ether); IR 1689, 1670, 1586, 1506, 1479, 1450, 1425, 1377, 1290, 1234, 1171, 1116, 874, 855, 753 cm^{-1}; ^{1}H NMR (300 MHz, $CDCl_3$) δ 7.40–7.31 (2H, m, 2 x Ar**H**), 7.21–7.11 (3H, m, 3 x Ar**H**), 7.05–6.96 (4H, m, 4 x Ar**H**), 4.65 (1H, s, C**H**), 3.37 (6H, s, 2 x NC**H**$_3$); ^{13}C NMR (75.5 MHz, $CDCl_3$) δ 167.3 (2 x C), 157.9 (C), 156.3 (C), 151.5 (C), 129.8 (2 x CH), 129.3 (2 x CH), 127.8 (C), 123.9 (CH), 119.4 (2 x CH), 119.0 (2 x CH), 54.8 (CH), 28.9 (2 x CH_3); HRMS (ESI +ve) Exact mass calculated for $C_{18}H_{17}N_2O_4$ [M+H]$^+$: 325.1183, found 325.1184.

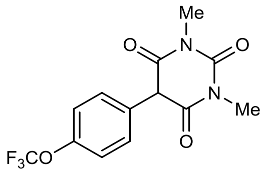

1,3-Dimethyl-5-(4-(trifluoromethoxy)phenyl)barbituric acid (2e). General Procedure B was followed using trifluoromethoxybenzene (2.00 mL). ^{1}H NMR analysis of the crude reaction mixture indicated a 4:1 mixture of regioisomers. Purification by flash column chromatography (40:40:20:1 PhMe/pet. ether/acetone/AcOH on AcOH-doped SiO_2) provided the title compound (428 mg, 68%) as a 4:1 mixture of regioisomers as a pale yellow oil. R_f 0.40 (40:40:20:1 PhMe/pet. ether/acetone/AcOH on AcOH-doped SiO_2); IR 1677, 1509, 1443, 1422, 1377, 1256, 1211, 1166, 1106, 755 cm^{-1}; Major isomer: ^{1}H NMR (400 MHz, $CDCl_3$) δ 7.31–7.22 (4H, m, 4 x Ar**H**), 4.70 (1H, s, C**H**), 3.37 (6H, s, 2 x NC**H**$_3$); ^{13}C NMR (100.6 MHz, $CDCl_3$) δ 166.7 (2 x C), 151.3 (C), 149.4 (q, J = 1.7 Hz, C), 131.9 (C), 129.7 (2 x CH), 121.7 (2 x CH), 120.3 (q, J = 257.8 Hz, CF_3), 54.7 (CH), 28.9 (2 x CH_3); Minor isomer: ^{1}H NMR diagnostic peaks (400 MHz, $CDCl_3$) δ 7.48–7.40 (2H, m, 2 x Ar**H**), 7.34 (1H, td, J = 7.5, 1.0 Hz, Ar**H**), 4.66 (1H, s, C**H**); ^{13}C NMR (100.6 MHz, $CDCl_3$) δ 166.1 (2 x C), 151.2 (C), 146.8 (C), 133.4 (CH), 130.5 (CH), 126.8 (CH), 125.8 (C), 120.2 (dd, J = 259.7 Hz, CF_3), 118.6 (q, J = 1.9 Hz, CH), 51.9 (CH), 28.8 (2 x CH); HRMS (ESI +ve) Exact mass calculated for $C_{13}H_{12}F_3N_2O_4$ [M+H]$^+$: 317.0744, found 317.0740.

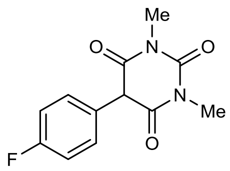

5-(4-Fluorophenyl)-1,3-dimethylbarbituric acid (2f). General Procedure B was followed using fluorobenzene (2.00 mL). ^{1}H NMR analysis of the crude reaction mixture indicated a 9:1 mixture of regioisomers. Purification by flash column chromatography (2–20% acetone/(1:1 PhMe/pet. ether); 1% AcOH on AcOH-doped silica gel) provided the title compound (350 mg, 78%) as a 9:1 mixture of regioisomers as a white crystalline solid. The pure *para*-isomer was obtained by recrystallization from IMS/pet. ether (284 mg, 57%). R_f 0.44 (40:40:20:1 PhMe/pet. ether/acetone/AcOH on AcOH-doped SiO_2); m.p. 110–112 °C (IMS/pet. ether); IR 1699, 1665, 1615, 1598, 1510, 1468, 1375, 1301, 1291, 1236, 1223, 1192, 1162, 1133, 1095, 840, 761 cm^{-1}; ^{1}H NMR (400 MHz, $CDCl_3$) δ 7.25–7.18 (2H, m, 2 x Ar**H**), 7.12–7.04 (2H, 2 × Ar**H**), 4.67 (1H, s, C**H**), 3.38 (6H, s, 2 x NC**H**$_3$); ^{13}C NMR (100.6 MHz, $CDCl_3$) δ 167.0 (2 x C), 162.7 (d, *J* = 248.7 Hz, C), 151.4 (C), 129.8 (d, *J* = 8.4 Hz, 2 x CH), 129.2 (d, *J* = 3.5 Hz, C), 116.3 (d, *J* = 22.1 Hz, 2 x CH), 54.6 (CH), 28.9 (2 x CH_3); ^{19}F NMR (282 MHz, $CDCl_3$) δ –112.5 (tt, *J* = 8.4, 5.0 Hz); HRMS (ESI +ve) Exact mass calculated for $C_{12}H_{12}FN_2O_3$ $[M+H]^+$: 251.0826, found 251.0829.

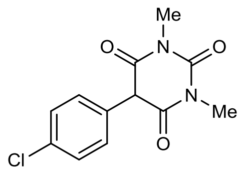

5-(4-Chlorophenyl)-1,3-dimethylbarbituric acid (2g). General Procedure B was followed using chlorobenzene (2.00 mL). ^{1}H NMR analysis of the crude reaction mixture indicated a 9:1 mixture of regioisomers. Purification by flash column chromatography (2–20% acetone/(1:1 PhMe/pet. ether); 1% AcOH on AcOH-doped silica gel) provided the title compound (394 mg, 74%) as a 9:1 mixture of regioisomers as a white crystalline solid. The pure *para*-isomer was obtained by recrystallization from IMS/pet. ether (252 mg, 47%). m.p. 126–127 °C (IMS/pet. ether); R_f 0.44 (40:40:20:1 PhMe/pet. ether/acetone/AcOH on AcOH-doped SiO_2); IR 1701, 1664, 1492, 1466, 1440, 1420, 1409, 1384, 13171, 1256, 1130, 1092, 1015, 842, 827, 767, 759 cm^{-1}; ^{1}H NMR (400 MHz, $CDCl_3$) δ 7.39–7.35 (2H, m, 2 × Ar**H**), 7.19–7.15 (2H, m, 2 × Ar**H**), 4.66 (1H, s, C**H**), 3.37 (6H, d, 2 x NC**H**$_3$); ^{13}C NMR (75.5 MHz, $CDCl_3$) δ 166.8 (2 x C), 151.4 (C), 135.0 (C), 131.8 (C), 129.5 (2 x CH), 129.4 (2 x CH), 54.8 (CH), 28.9 (CH_3); HRMS (ESI +ve) Exact mass calculated for $C_{12}H_{12}ClN_2O_3$ $[M+H]^+$: 267.0531, found 267.0536.

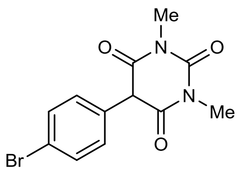

5-(4-Bromophenyl)-1,3-dimethylbarbituric acid (2h). General Procedure B was followed using bromobenzene (2.00 mL). ^{1}H NMR analysis of the crude reaction mixture indicated a 9:1 mixture of regioisomers. Purification by flash column chromatography (2–20% acetone/(1:1 PhMe/Pet. ether); 1% AcOH on AcOH-doped silica gel) provided the title compound (435 mg, 70%) as a 10:1 mixture of

regioisomers as a white crystalline solid. The pure *para*-isomer was obtained by recrystallization from IMS/pet. ether (313 mg, 50%). m.p. 118–122 °C (IMS/pet. ether); R_f 0.44 (40:40:20:1 PhMe/pet. ether/acetone/AcOH on AcOH-doped SiO_2); m.p. 118–122 °C (IMS/Pet. ether); IR 1697, 1679, 1666, 1605, 1492, 1483, 1369, 1009, 829, 759 cm^{-1}; ^{1}H NMR (300 MHz, $CDCl_3$) δ 7.55–7.49 (2H, m, 2 × Ar**H**), 7.14–7.08 (2H, m, 2 x Ar**H**), 4.64 (1H, s, C**H**), 3.37 (6H, s, 2 x NC**H**$_3$); ^{13}C NMR (75.5 MHz, $CDCl_3$) δ 166.7 (2 x C), 151.3 (C), 132.5 (C), 132.3 (2 x CH), 129.7 (2 x CH), 123.1 (C), 54.9 (CH), 28.9 (CH_3); HRMS (ESI +ve) Exact mass calculated for $C_{12}H_{12}BrN_2O_3$ $[M+H]^+$: 311.0026, found 311.0019.

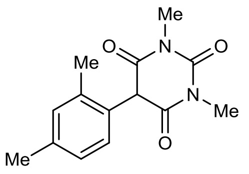

1,3-Dimethyl-5-(2,4-dimethylphenyl)barbituric acid (2i). General Procedure B was followed using *m*-xylene (2.00 mL). ^{1}H NMR analysis of the crude reaction mixture indicated a >19:1 mixture of regioisomers. Purification by flash column chromatography (40:40:20:1 PhMe/pet. ether/acetone/AcOH on AcOH-doped SiO_2) provided the title compound (369 mg, 71%) as a single regioisomer as a pale yellow waxy solid. R_f 0.60 (40:40:20:1 PhMe/pet. ether/acetone/AcOH on AcOH-doped SiO_2); IR 1671, 1440, 1419, 1374, 1286, 1262, 1106, 1031, 753 cm^{-1}; ^{1}H NMR (300 MHz, $CDCl_3$) δ 7.07 (1H, br s, Ar**H**), 7.00 (1H, br d, *J* = 7.8 Hz, Ar**H**), 6.87 (1H, d, *J* = 7.8 Hz, Ar**H**), 4.82 (1H, s, C**H**), 3.36 (6H, s, 2 x NC**H**$_3$), 2.35 (3H, s, ArC**H**$_3$), 2.31 (3H, s, ArC**H**$_3$); ^{13}C NMR (100.4 MHz, $CDCl_3$) δ 167.3 (2 x C), 151.6 (C), 138.5 (C), 136.4 (C), 132.1 (CH), 129.8 (C), 128.0 (CH), 127.3 (CH), 52.6 (CH), 28.7 (2 x CH_3), 20.9 (CH_3), 19.7 (CH_3); HRMS (ESI +ve) Exact mass calculated for $C_{14}H_{17}N_2O_3$ $[M+H]^+$: 261.1234, found 261.1234.

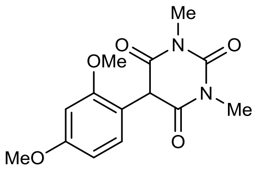

5-(2,4-Dimethoxyphenyl)-1,3-dimethylbarbituric acid (2j). General Procedure B was followed using 1,3-dimethoxybenzene (2.00 mL). ^{1}H NMR analysis of the crude reaction mixture indicated a >19:1 mixture of regioisomers. Purification by flash column chromatography (2–16% acetone/(1:1 PhMe/pet. ether); 1% AcOH on AcOH-doped silica gel) provided the title compound (444 mg, 76%) as a single regioisomer as a white crystalline solid. R_f 0.40 (40:40:20:1 PhMe/pet. ether/acetone/AcOH on AcOH-doped SiO_2); m.p. 119–120 °C (IMS/pet. ether); IR 1687, 1671, 1467, 1439, 1418, 1378, 1294, 1209, 1159, 1109, 1030, 831, 760 cm^{-1}; ^{1}H NMR (500 MHz, $(CD_3)_2SO$) δ 7.22 (1H, d, *J* = 8.2 Hz, Ar**H**), 6.55 (1H, d, *J* = 2.4 Hz, Ar**H**), 6.53 (1H, dd, *J* = 8.2, 2.4 Hz, Ar**H**), 4.90 (1H, s, C**H**), 3.76 (3H, s, OC**H**$_3$), 3.63 (3H, s, OC**H**$_3$), 3.18 (6H, s, 2 x OC**H**$_3$); ^{13}C NMR (125.8 MHz, $(CD_3)_2SO$) δ 167.9 (2 x C), 160.6 (C), 157.1 (C), 151.8 (C), 132.9 (CH), 116.5

(C), 104.9 (CH), 98.9 (CH), 55.9 (CH_3), 55.3 (CH_3), 51.2 (CH), 28.2 (CH_3); HRMS (ESI –ve) Exact mass calculated for $C_{14}H_{15}N_2O_5$ $[M–H]^-$: 291.0896, found 291.0978.

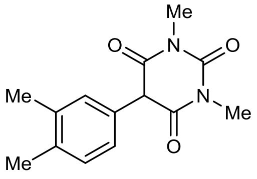

2ka

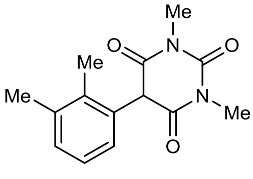

2kb

1,3-Dimethyl-5-(3,4-dimethylphenyl)barbituric acid (2ka) and 1,3-dimethyl-5-(2,3-dimethylphenyl)barbituric acid (2kb). A modification of General Procedure B (in that the reaction scale was 1.00 mmol) was followed using **1a** (182 mg, 1.00 mmol), $Rh_2(esp)_2$ (0.8 mg, 1 µmol), and *o*-xylene (1.00 mL). ^{1}H NMR analysis of the crude reaction mixture indicated a 3:1 mixture of isomers. Purification by flash column chromatography (1–10% acetone/(1:1 PhMe/pet. ether); 1% AcOH on AcOH-doped silica gel) provided 1,3-dimethyl-5-(3,4-dimethylphenyl)barbituric acid (**2ka**, 167 mg, 64%) and 1,3-dimethyl-5-(2,3-dimethylphenyl)barbituric acid (**2kb**, 36 mg, 14%) as white crystalline solids.

Major regioisomer **2ka**: R_f 0.65 (40:40:20:1 PhMe/pet. ether/acetone/AcOH on AcOH-doped SiO_2); m.p. 160–161 °C (IMS/pet. ether); IR 1699, 1659, 1459, 1440, 1369, 1270, 1130, 762, 754 cm^{-1}; ^{1}H NMR (300 MHz, $CDCl_3$) δ 7.13 (1H, d, *J* = 7.8 Hz, Ar**H**), 6.99 (1H, d, *J* = 1.9 Hz, Ar**H**), 6.93 (1H, dd, *J* = 7.8, 1.9 Hz, Ar**H**), 4.60 (1H, s, C**H**), 3.36 (6H, s, 2 x NC**H**$_3$), 2.25 (3H, s, ArC**H**$_3$), 2.24 (3H, s, ArC**H**$_3$); ^{13}C NMR (75.5 MHz, $CDCl_3$) δ 167.5 (2 x C), 151.7 (C), 137.9 (C), 137.5 (C), 131.0 (C), 130.5 (CH), 128.9 (CH), 124.9 (CH), 55.3 (CH), 28.8 (2 x CH_3), 19.8 (CH_3), 19.4 (CH_3); HRMS (ESI +ve) Exact mass calculated for $C_{14}H_{17}N_2O_3$ $[M+H]^+$: 261.1234, found 261.1238.

Minor isomer **2kb**: R_f 0.55 (40:40:20:1 PhMe/Pet. ether/acetone/AcOH on AcOH-doped SiO_2); m.p. 166–168 °C (IMS/Pet. ether); IR 1677, 1442, 1420, 1373, 1266, 1124, 1095, 798, 755 cm^{-1}; ^{1}H NMR (300 MHz, $CDCl_3$) δ 7.16 (1H, d, *J* = 7.4 Hz, Ar**H**), 7.09 (1H, t, *J* = 7.4 Hz, Ar**H**), 6.86 (1H, d, *J* = 7.4 Hz, Ar**H**), 4.90 (1H, s, C**H**), 3.37 (6H, s, 2 x NC**H**$_3$), 2.32 (3H, s, ArC**H**$_3$), 2.24 (3H, s, ArC**H**$_3$); ^{13}C NMR (75.5 MHz, $CDCl_3$) δ 167.4 (2 x C), 151.6 (C), 138.2 (C), 135.2 (C), 132.6 (C), 130.6 (CH), 126.4 (CH), 126.2 (CH), 53.5 (CH), 28.8 (2 x CH_3), 20.9 (CH_3), 16.1 (CH_3); HRMS (ESI +ve) Exact mass calculated for $C_{14}H_{17}N_2O_3$ $[M+H]^+$: 261.1234, found 261.1233.

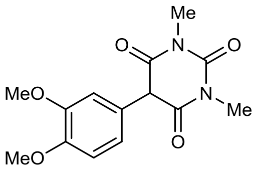

1,3-Dimethyl-5-(3,4-dimethoxyphenyl)barbituric acid (2l). General Procedure B was followed using veratrole (2.00 mL). ^{1}H NMR analysis of the crude reaction mixture indicated a 3:1 mixture of regioisomers. Purification by flash column chromatography (40:40:20:1 PhMe/pet. ether/acetone/AcOH on AcOH-doped SiO_2) provided the title compound (508 mg, 87%) as a 3:1 mixture of regioisomers as a white crystalline solid. The pure major isomer was obtained by recrystallization from IMS/pet.

ether (204 mg, 35%). R_f 0.30 (40:40:20:1 PhMe/pet. ether/acetone/AcOH on AcOH-doped SiO_2); m.p. 129–130 °C (IMS/pet. ether); IR 1698, 1607, 1516, 1329, 1248, 1133, 1029, 809, 756 cm^{-1}; ^1H NMR (400 MHz, $CDCl_3$) δ 6.84 (1H, d, *J* = 8.3 Hz, Ar**H**), 6.81 (1H, d, *J* = 2.2 Hz, Ar**H**), 6.69 (1H, dd, *J* = 8.3, 2.2 Hz, Ar**H**), 4.61 (1H, s, C**H**), 3.89 (3H, s, OC**H**3), 3.87 (3 H, s, OC**H**3), 3.37 (6H, s, 2 x NC**H**3); ^{13}C NMR (100.6 MHz, $CDCl_3$) δ 167.4 (2 x C), 151.6 (C), 149.5 (C), 125.8 (C), 119.4 (CH), 111.6 (CH), 111.57 (CH), 56.0 (CH_3), 55.9 (CH_3), 55.1 (CH), 28.9 (2 x CH_3); HRMS (ESI +ve) Exact mass calculated for $C_{14}H_{17}N_2O_5$ $[M+H]^+$: 293.1132, found 293.1128.

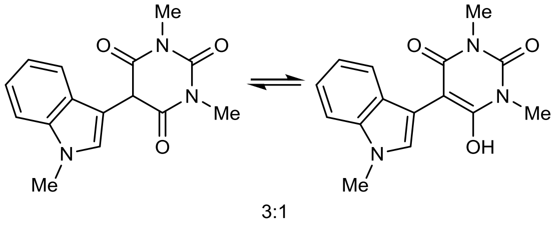

1,3-Dimethyl-5-(1-methylindol-3-yl)barbituric acid (3). A solution of 5-arylbarituric acid **1a** (182 mg, 1.00 mmol) and $Rh_2(esp)_2$ (0.8 mg, 1 µmol) in 1-methylindole (1.00 mL) was stirred at 60 °C for 24 h. ^1H NMR analysis of the crude reaction mixture indicated a >19:1 mixture of isomers. The reaction mixture was cooled to room temperature, diluted with Et_2O (4 mL) and the precipitate was collected by vacuum filtration to provide the title compound (238 mg, 73%) as a >19:1 mixture of regioisomers as a white crystalline solid. R_f 0.38 (40:40:20:1 PhMe/pet. ether/acetone/AcOH on AcOH-doped SiO_2); m.p. 223–224 °C (IMS); IR 1687, 1604, 1617, 1477, 1457, 1242, 1210, 1198, 1070, 739 cm^{-1}; ^1H NMR (270 MHz, $(CD_3)_2SO$, 70 °C*) δ 7.55–7.37 (2H, m, 2 x Ar**H**), 7.28 (1H, s, NC**H**=C), 7.18 (1H, t, *J* = 7.5 Hz, Ar**H**), 7.05 (1H, t, *J* = 7.5 Hz, Ar**H**), 5.12 (1H, br s, C**H**), 3.77 (3H, s, Indole NC**H**3), 3.23 (6H, s, 2 x barbiturate NC**H**3); ^{13}C NMR keto tautomer (125.8 MHz, $(CD_3)_2SO$, room temperature) δ 167.8 (2 x C), 151.9 (C), 136.3 (C), 128.9 (CH), 126.2 (C), 121.5 (CH), 119.2 (CH), 118.6 (CH), 109.9 (CH), 106.8 (C), 47.5 (CH), 32.4 (CH_3), 28.4 (CH_3); ^{13}C NMR enol tautomer, 2 x C not resolved (125.8 MHz, $(CD_3)_2SO$, room temperature) δ 150.8 (C), 136.6 (C), 130.8 (CH), 128.1 (C), 120.8 (CH), 119.9 (CH), 118.5 (CH), 109.5 (CH), 103.9 (C), 85.1 (C), 32.4 (CH_3, assumed coincident), 28.4 (CH_3, assumed coincident). HRMS (ESI +ve) Exact mass calculated for $C_{15}H_{16}N_3O_3$ $[M+H]^+$: 286.1186, found 286.1184.

*A tautomeric mixture is observed at room temperature.

NOESY correlations $H_b \leftrightarrow H_c$ (red) and $H_a \leftrightarrow H_g$ (blue) indicate that the indole **3** is substituted at C3.

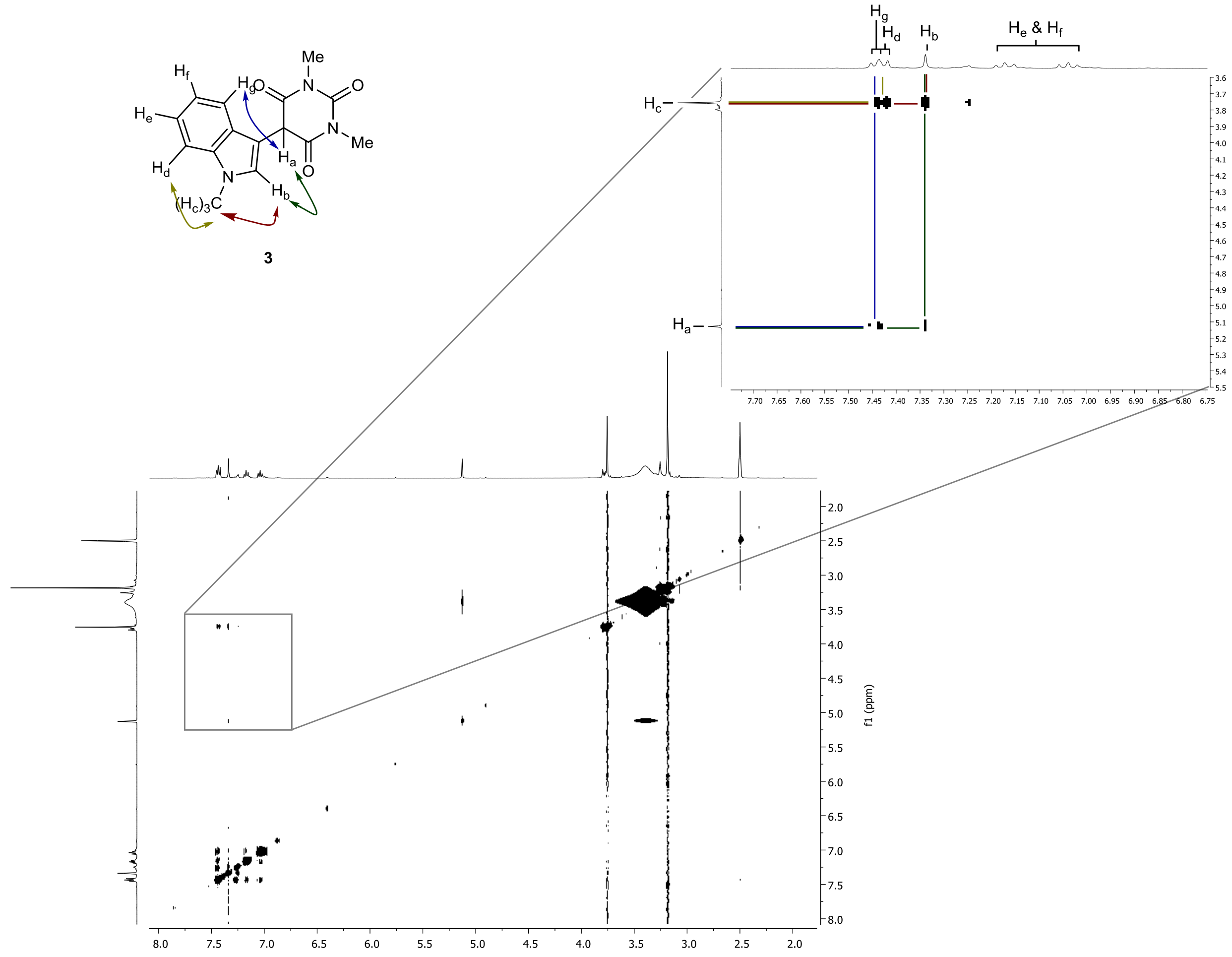

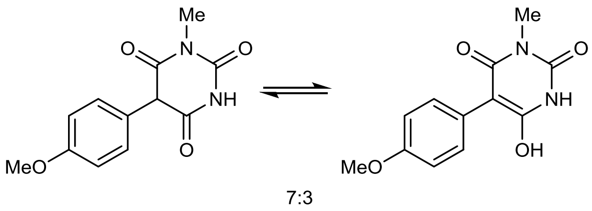

5-(4-Methoxyphenyl)-1-methylbarbituric acid (4a). A slurry of 5-diazobarbituric acid **1b** (84 mg, 0.50 mmol), $Rh_2(esp)_2$ (0.4 mg, 0.5 μmol), and anisole (0.50 mL) was stirred at 60 °C for 24 h. ^{1}H NMR analysis of the crude reaction mixture indicated a 10:1 mixture of regioisomers. The reaction mixture was concentrated *in vacuo* and purified by flash column chromatography (10–60% acetone/PhMe; 1% AcOH on AcOH-doped silica gel) to provide the title compound (84 mg, 68%) as a single regioisomer as a white crystalline solid. R_f 0.08 (40:40:20:1 PhMe/pet. ether/acetone/AcOH on AcOH-doped SiO_2); m.p. 187–188 °C (IMS/pet. ether); IR 2973, 1701, 1609, 1573, 1532, 1513, 1471, 1291, 1243, 1175, 1027, 828, 792, 779 cm^{-1}; ^{1}H NMR (500 MHz, $(CD_3)_2SO$) δ 11.53 (0.7H, s, N**H** keto), 11.04 (0.3H, s, N**H** enol), 7.18 (2H, d, J = 8.6 Hz, Ar**H**), 6.90 (2H, d, J = 8.6 Hz, 2 x Ar**H**), 4.82 (0.7H, s, C**H** keto), 3.74 (3H, s, OC**H**$_3$), 3.11 (3H, s, NC**H**$_3$); ^{13}C NMR keto tautomer (125.8 MHz, $(CD_3)_2SO$) δ 168.8 (C), 168.3 (C), 158.8 (C), 151.3 (C), 130.3 (2 x CH), 126.6 (C), 114.0 (2 x CH), 55.1 (CH_3), 54.4 (CH), 27.4 (CH_3); ^{13}C NMR enol tautomer (125.8 MHz, $(CD_3)_2SO$) δ 163.3 (C), 157.9 (C), 156.7 (C), 150.2

(C), 132.4 (2 x CH), 124.1 (C), 113.3 (2 x CH), 91.8 (C), 55.0 (CH_3), 26.9 (CH_3); HRMS (ESI +ve) Exact mass calculated for $C_{12}H_{13}N_2O_4$ $[M+H]^+$: 249.0870, found 249.0876.

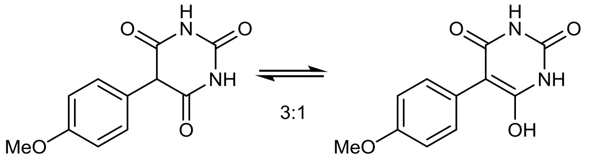

5-(4-Methoxyphenyl)barbituric acid (4b). A slurry of 5-diazobarbituric acid **1c** (154 mg, 1.00 mmol), $Rh_2(esp)_2$ (0.8 mg, 1 µmol), and anisole (1.00 mL) was stirred at 90 °C for 24 h. ^{1}H NMR analysis of the crude reaction mixture indicated a 9:1 mixture of regioisomers. The reaction mixture was cooled to room temperature, diluted with pet. ether (4 mL), and the precipitate was collected by vacuum filtration to provide the title compound (218 mg, 93%) as a 9:1 mixture of regioisomers as a pale yellow crystalline solid. R_f 0.20–0.70 streak (70:20:10:1 EtOAc/IPA/H_2O/AcOH on AcOH-doped SiO_2); m.p. 236–239 °C (IMS/pet. ether); IR 3210; 3092; 1747, 1714, 1682, 1517, 1427, 1357, 1257, 1226, 1182, 1035, 839, 829, 794, 762 cm^{-1}; ^{1}H NMR (500 MHz, $(CD_3)_2SO$) Major isomer δ 11.34 (1.5H, s, 2 x N**H** keto), 10.71 (0.5H, s, 2 x N**H** enol), 7.17 (2H, d, *J* = 8.5 Hz, Ar**H**), 6.91 (2H, d, *J* = 8.5 Hz, Ar**H**), 4.72 (1H, s, C**H** keto), 3.74 (3H, s, OC**H**$_3$); Minor isomer, only keto form observed δ 11.31 (2H, s, 2 x N**H**), 7.32 (1H, ddd, *J* = 8.1, 7.6, 1.6 Hz, Ar**H**), 7.27 (1H, dd, *J* = 7.4, 1.6 Hz, Ar**H**)), 6.99 (1H, d, *J* = 8.2 Hz, Ar**H**), 6.95 (1H, td, *J* = 7.4, 0.8 Hz, Ar**H**), 4.87 (1H, s, C**H**), 3.67 (3H, s, OC**H**$_3$); ^{13}C NMR (126 MHz, $(CD_3)_2SO$) Major isomer, keto form δ 169.5 (2 x C), 158.8 (C), 151.0 (C), 130.3 (2 x CH), 126.5 (C), 114.7 (2 x CH), 55.1 (CH_3), 54.2 (CH); Major isomer, enol form δ 161.1 (br, 2 x C), 157.9 (C), 149.8 (C), 132.3 (2 x CH), 123.8 (C), 113.3 (2 x CH), 92.1 (C), 55.1 (CH_3); Minor isomer, only keto observed δ 169.3 (2 x C), 156.2 (C), 150.9 (C), 133.0 (CH), 129.4 (CH), 123.9 (C), 120.5 (CH), 111.3 (CH), 55.7 (CH_3), 51.8 (CH); HRMS (ESI +ve) Exact mass calculated for $C_{11}H_{11}N_2O_4$ $[M+H]^+$: 235.0713, found 235.0719.

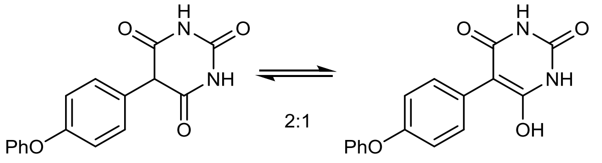

5-(4-Phenoxyphenyl)barbituric acid (4c). A slurry of 5-diazobarbituric acid **1c** (77 mg, 0.50 mmol), $Rh_2(esp)_2$ (1.0 mg, 1.3 µmol), and diphenyl ether (0.50 mL) was stirred at 120 °C for 24 h. ^{1}H NMR analysis of the crude reaction mixture indicated a 9:1 mixture of regioisomers. The reaction mixture was cooled to room temperature, diluted with pet. ether (4 mL), and the was precipitate collected by vacuum filtration to provide the title compound (132 mg, 89%) as a 9:1 mixture of regioisomers as a pale yellow crystalline solid. Trituration of 23 mg with Et_2O (5 mL) provided the title compound (18 mg, 69% from **1c**) as a single isomer. R_f 0.30–0.80 streak (70:20:10:1 EtOAc/IPA/H_2O/AcOH on AcOH-doped SiO_2); m.p. 240–244 °C (IMS/pet. ether); IR 3215, 3091,

1745, 1714, 1682, 1588, 1509, 1489, 1429, 1358, 1253, 1235, 797, 756, 693 cm^{-1}; ^{1}H NMR (500 MHz, $(CD_3)_2SO$) δ 11.37 (1.3 H, s, N**H** keto), 10.75 (0.7 H, s, N**H** enol), 7.40 (2H, t, *J* = 7.9 Hz, 2 x Ar**H**), 7.32–7.24 (2H, m, 2 x Ar**H**), 7.19–7.11 (1H, m, Ar**H**), 7.03 (2H, d, *J* = 7.9 Hz, 2 x Ar**H**), 6.97 (2H, d, *J* = 8.0 Hz, 2 x Ar**H**), 4.85 (0.7H, s, C**H** keto); ^{13}C NMR (126 MHz, $(CD_3)_2SO$) Keto form δ 169.2 (2 x C), 156.4 (C), 156.3 (C), 150.9 (C), 131.1 (2 x CH), 130.1 (2 x CH) 129.2 (C), 123.7 (CH), 118.9 (2 x CH), 118.4 (2 x CH), 54.3 (CH); Enol form δ 161.2 (br, 2 x C), 156.9 (C), 154.9 (C), 149.8 (C), 132.8 (2 x CH), 130.0 (2 x CH), 127.1 (C), 123.3 (CH), 118.5 (2 x CH), 118.0 (2 x CH), 91.8 (C); HRMS (ESI +ve) Exact mass calculated for $C_{11}H_{11}N_2O_4$ $[M+H]^+$: HRMS (ESI +ve) Exact mass calculated for $C_{16}H_{13}N_2O_4$ $[M+H]^+$: 297.0870, found 297.0856.

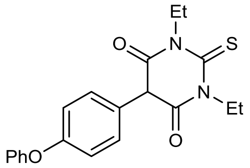

1,3-Diethyl-5-(4-phenoxyphenyl)-2-thiobarbituric acid (4d). A solution of 5-diazothiobarbituric acid **1d** (113 mg, 0.50 mmol) and $Rh_2(esp)_2$ (0.4 mg, 0.5 μmol) in diphenyl ether was stirred at 40 °C for 24 h. ^{1}H NMR analysis of the crude reaction mixture indicated a 6:1 mixture of regioisomers. The reaction mixture was concentrated *in vacuo* and purification by flash column chromatography (1–5% Et_2O/PhMe; 1% AcOH on AcOH-doped silica gel) provided the title compound (130 mg, 71%) as a 6:1 mixture of isomers as a pale yellow gum. R_f 0.60 (40:40:20:1 PhMe/pet. ether/acetone/AcOH on AcOH-doped SiO_2); IR 1723, 1691, 1587, 1505, 1488, 1434, 1388, 1371, 1341, 1296, 1241, 1206, 1171, 1109, 872, 832, 783, 758 cm^{-1}; ^{1}H NMR (300 MHz, $CDCl_3$) δ 7.40–7.32 (2H, m, 2 x Ar**H**), 7.21–7.11 (3H, m, 3 x Ar**H**), 7.05–6.96 (4H, m, 4 x Ar**H**), 4.68 (1H, s, C**H**), 4.52 (2H, dq, *J* = 13.4, 7.0 Hz, 2 x $C\mathbf{H}_{2a}$), 4.41 (2H, dq, *J* = 13.4, 7.0 Hz, 2 x $C\mathbf{H}_{2b}$), 1.26 (6H, t, *J* = 7.0 Hz, 2 x $C\mathbf{H}_3$); Minor isomer, diagnostic peaks δ 7.47–7.40 (1H, m, Ar**H**), 7.32–7.28 (2H, m, 2 x Ar**H**), 7.11–7.06 (3H, m, 3 x Ar**H**), 6.90–6.81 (3H, m, 3 x Ar**H**), 4.60 (1H, s, C**H**), 1.13 (1H, t, *J* = 7.0 Hz, 2 x $C\mathbf{H}_3$); ^{13}C NMR (75.5 MHz, $CDCl_3$) Major isomer δ 179.6 (C), 165.5 (2 x C), 158.1 (C), 156.2 (C), 129.9 (2 x CH), 128.8 (2 x C), 127.6 (C), 124.0 (CH), 119.6 (2 x CH), 119.1 (2 x CH), 55.7 (CH), 43.6 (2 x CH_2), 12.3 (2 x CH_3); Minor isomer δ 179.5 (C), 165.2 (2 x C), 158.5 (C), 155.9 (C), 132.9 (CH), 130.1 (CH), 129.8 (2 x CH), 126.2 (C), 123.7 (CH), 120.1 (CH), 118.6 (2 x CH), 118.1 (CH), 53.2 (CH), 43.7 (2 x CH_2), 12.0 (2 x CH_3); HRMS (ESI +ve) Exact mass calculated for $C_{20}H_{21}N_2O_3S$ $[M+H]^+$: 369.1267, found 369.1278.

Synthesis of MMP Inhibitor 8

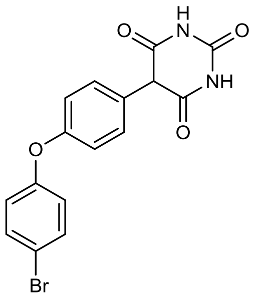

5-[4-(4-Bromophenoxy)phenyl]barbituric acid (5).[6] A suspension of 5-diazobarbituric acid **1c** (77 mg, 0.50 mmol) and $Rh_2(esp)_2$ (1.9 mg, 2.5 µmol) in 4-bromophenyl phenyl ether (1 mL) was stirred at 120 °C for 16 h. The reaction mixture was cooled to room temperature, diluted with pet. ether (5 mL), and the precipitate collected by vacuum filtration. The filter cake was washed with pet. ether to give the title compound (163 mg, 87%) as a 9:1 ratio of regioisomers as a pale yellow crystalline solid. Physical and spectroscopic data were in accordance with the literature.[6]

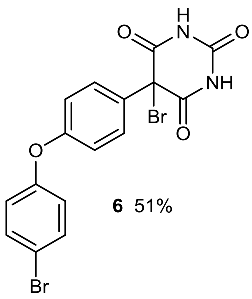

6 51%

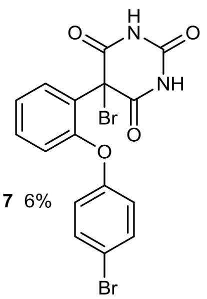

7 6%

5-Bromo-5-(4-(4-bromophenoxy)phenyl)barbituric acid (6)[6] and 5-bromo-5-(2-(4-bromophenoxy)phenyl)barbituric acid (7). To a suspension of 5-arylbarbituric acid **5** (9:1 regioisomeric mixture, 146 mg, 0.39 mmol) in CH_2Cl_2 (10 mL) was added pyridinium tribromide (125 mg, 0.389 mmol) in one portion. The mixture was stirred at room temperature for 1 h, pre-adsorbed onto silica gel and purified by flash column chromatography (2–14% acetone/PhMe) to give bromide **7** (11 mg, 6%) and bromide **6** (91 mg, 51%) as white crystalline solids. Data for **6** were as described previously.[6] Data for **7**: R_f 0.50 (20% acetone/PhMe); m.p. 224–226 °C decomp ($CHCl_3$); IR 3209, 3113, 1719, 1701, 1478, 1454, 1343, 1236, 1208, 1010, 837, 771, 755 cm^{-1}; ^{1}H NMR (500 MHz, CD_3CN) δ 9.35 (2H, s, 2 x N**H**), 7.96 (1H, dd, *J* = 7.8, 1.6 Hz, Ar**H**), 7.55–7.49 (2H, m, 2 x Ar**H**), 7.46 (1H, ddd, *J* = 8.2, 7.6, 1.6 Hz, Ar**H**), 7.31 (1H, td, *J* = 7.7, 1.1 Hz, Ar**H**), 6.87–6.83 (2H, m, 2 x Ar**H**), 6.84 (1H, dd, *J* = 8.1, 1.1 Hz, Ar**H**); ^{13}C NMR (125.8 MHz, CD_3CN) δ 167.9 (2 x C), 155.2 (C), 153.7 (C), 148.6 (C), 134.0 (2 x CH), 133.8 (CH), 132.6 (CH), 125.6 (C), 125.1 (CH), 121.8 (2 x CH), 118.3 (C), 117.9 (CH); HRMS (ESI +ve) Exact mass calculated for $C_{16}H_{10}Br_2N_2NaO_4$ $[M+Na]^+$: 476.8880, found 4769.8900.

6. Breyholz, H.-J.; Schäfers, M.; Wagner, S.; Höltke, C.; Faust, A.; Rabeneck, H.; Levkau, B.; Schober, O.; Kopka, K. *J. Med. Chem.*, **2005**, *48*, 3400–3409.

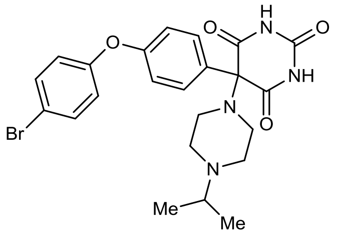

5-(4-(4-Bromophenoxy)phenyl)-5-(4-isoproplypiperazin-1-yl)barbituric acid (8).[6] MMP inhibitor **8** was synthesized as described previously.[6] To a solution of bromide **6** (59 mg, 0.13 mmol) in MeOH (1.00 mL) was added *N*-isopropylpiperazine (40 µL, 0.28 mmol) and the mixture was stirred at room temperature for 15 h. The precipitate was collected by filtration and the filter cake washed with cold MeOH (0.50 mL) to provide the MMP inhibitor **8** (46 mg, 70%) as a white crystalline solid. Physical and spectroscopic data were in accordance with the literature.[6]

Rh(II)-Catalyzed Coupling of 1,3-Dimethyl-5-diazobarbituric Acid with Cyclohexane

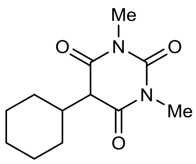

5-Cyclohexyl-1,3-dimethylbarbituric acid (9). A suspension of 5-diazobarbituric acid **1a** (91 mg, 0.50 mmol) and $Rh_2(esp)_2$ (1.0 mg, 1.3 µmol) in cyclohexane (2.00 mL) was stirred at 55 °C for 24 h. The reaction mixture was concentrated *in vacuo* and purified by flash column chromatography (2–10% acetone/pet. ether) to provide the title compound (99 mg, 83%) as a white crystalline solid. R_f 0.70 (20% acetone/PhMe); m.p. 129–130 °C (PhMe); IR 2947, 2908, 2851, 1692, 1660, 1461, 1442, 1414, 1371, 1321, 1264, 1146, 1115, 763 cm^{-1}; ^{1}H NMR (300 MHz, $CDCl_3$) δ 3.32 (1H, d, *J* = 3.9 Hz, C**H**Cy), 3.29 (6H, s, 2 x $NC\mathbf{H}_3$), 2.29–2.09 (1H, m, CHC**H**CH_2), 1.86–1.70 (2H, m, $C\mathbf{H}_2$), 1.70–1.56 (3H, m, $C\mathbf{H}_2$), 1.35–1.17 (2H, m, $C\mathbf{H}_2$), 1.19–0.98 (1H, m, $C\mathbf{H}_2$); ^{13}C NMR (75.5 MHz, $CDCl_3$) δ 168.5 (2 x C), 151.9 (C), 55.5 (CH), 44.1 (CH), 29.9 (2 x CH_2), 28.3 (2 x CH_3), 26.2 (2 x CH_2), 25.5 (CH_2); HRMS (ESI +ve) Exact mass calculated for $C_{12}H_{18}N_2NaO_3$ $[M+Na]^+$: 261.1210, found 261.1207.

NMR Spectra

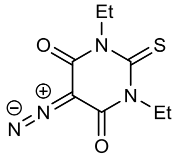

1d

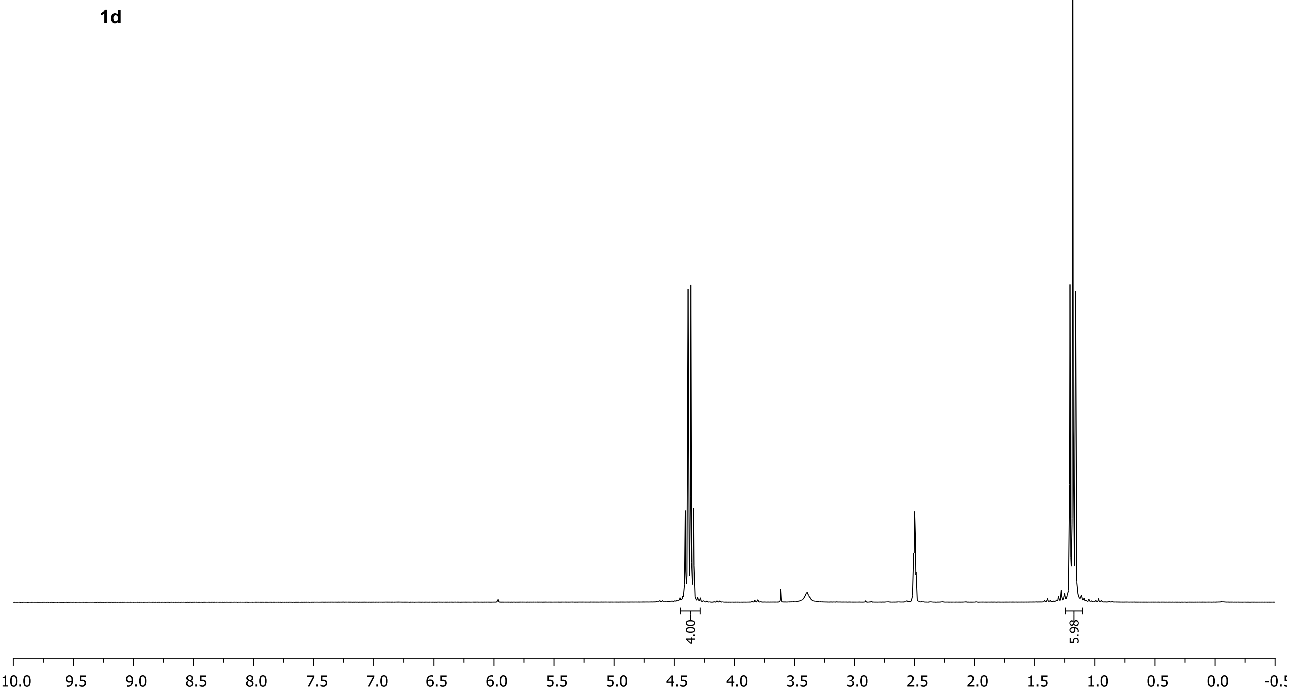

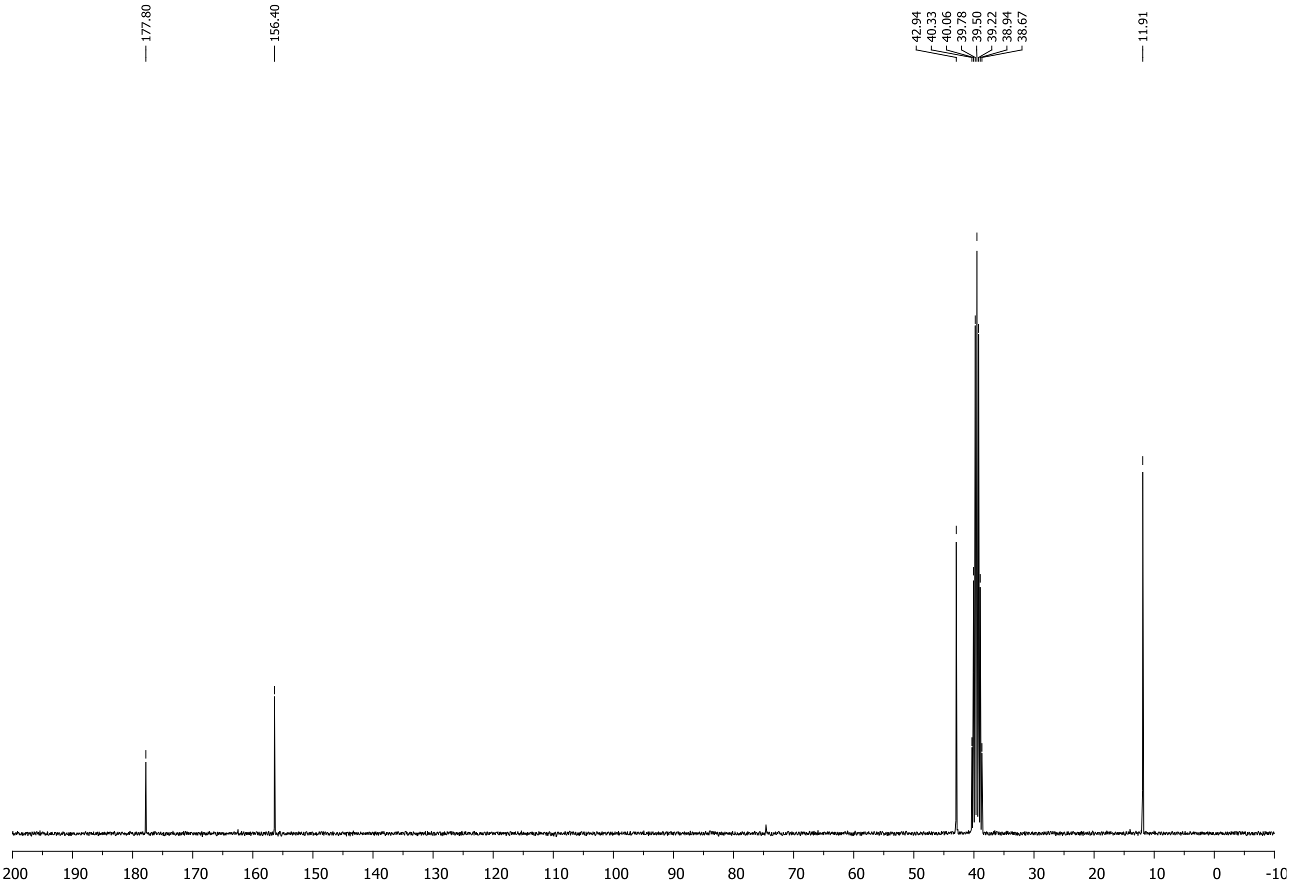

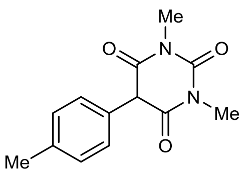

2b

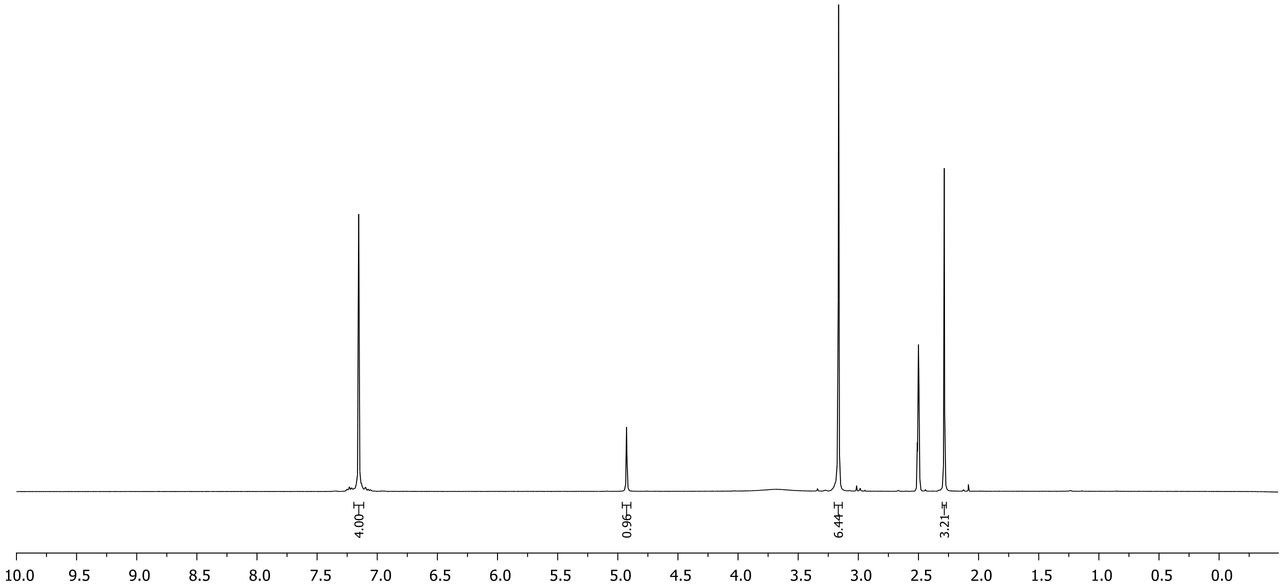

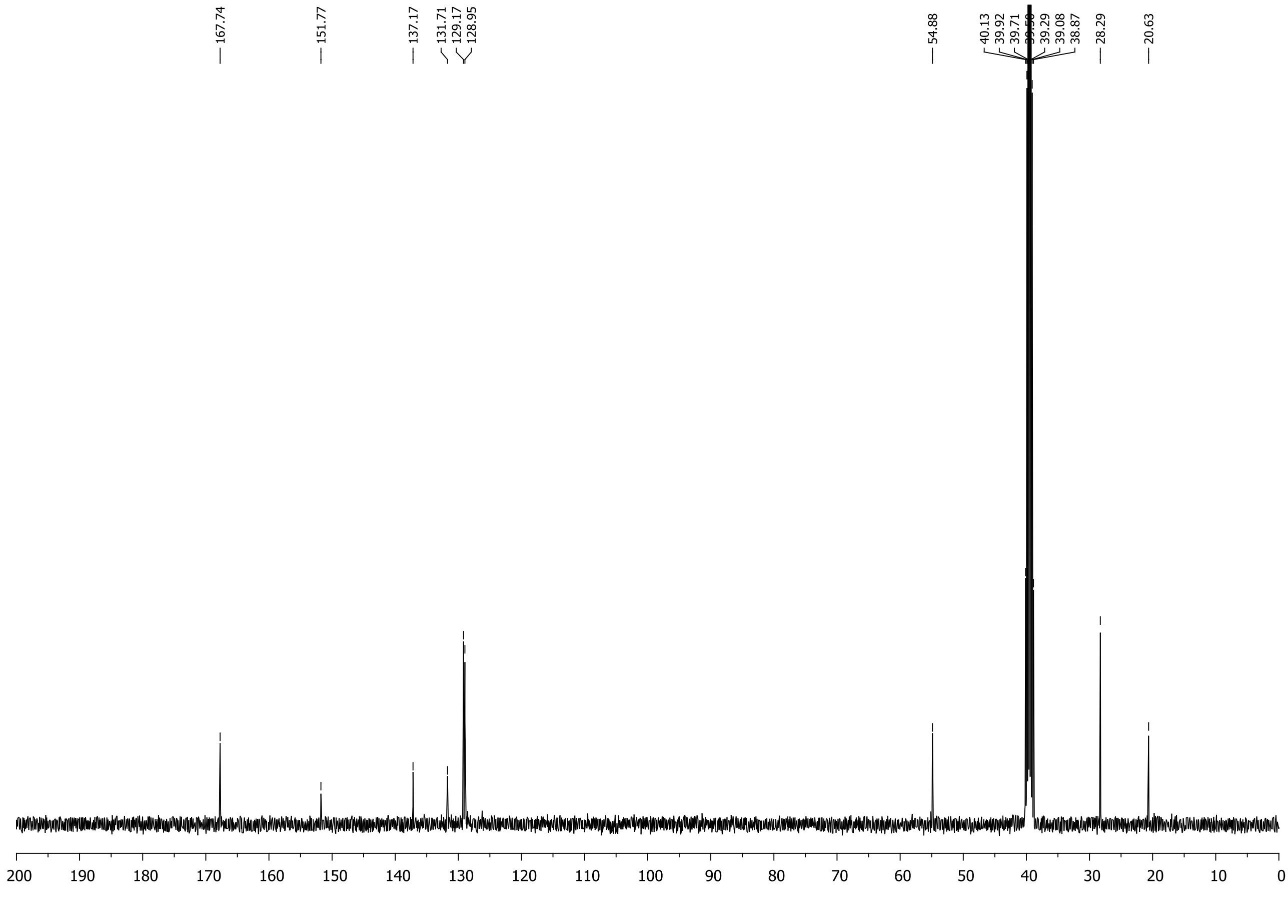

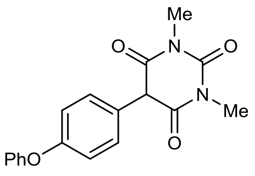
Me
O
N
O
N
Me
O
PhO

2d

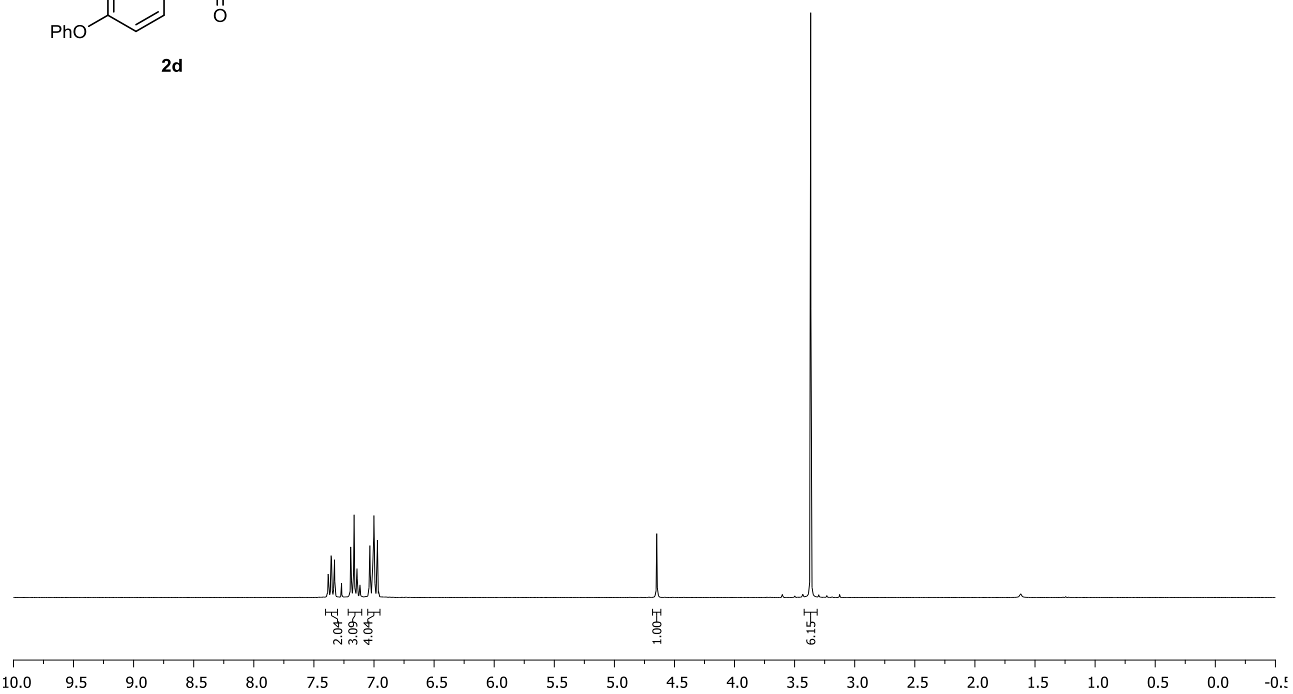
2.04
3.09
4.04
1.00
6.15
10.0 9.5 9.0 8.5 8.0 7.5 7.0 6.5 6.0 5.5 5.0 4.5 4.0 3.5 3.0 2.5 2.0 1.5 1.0 0.5 0.0

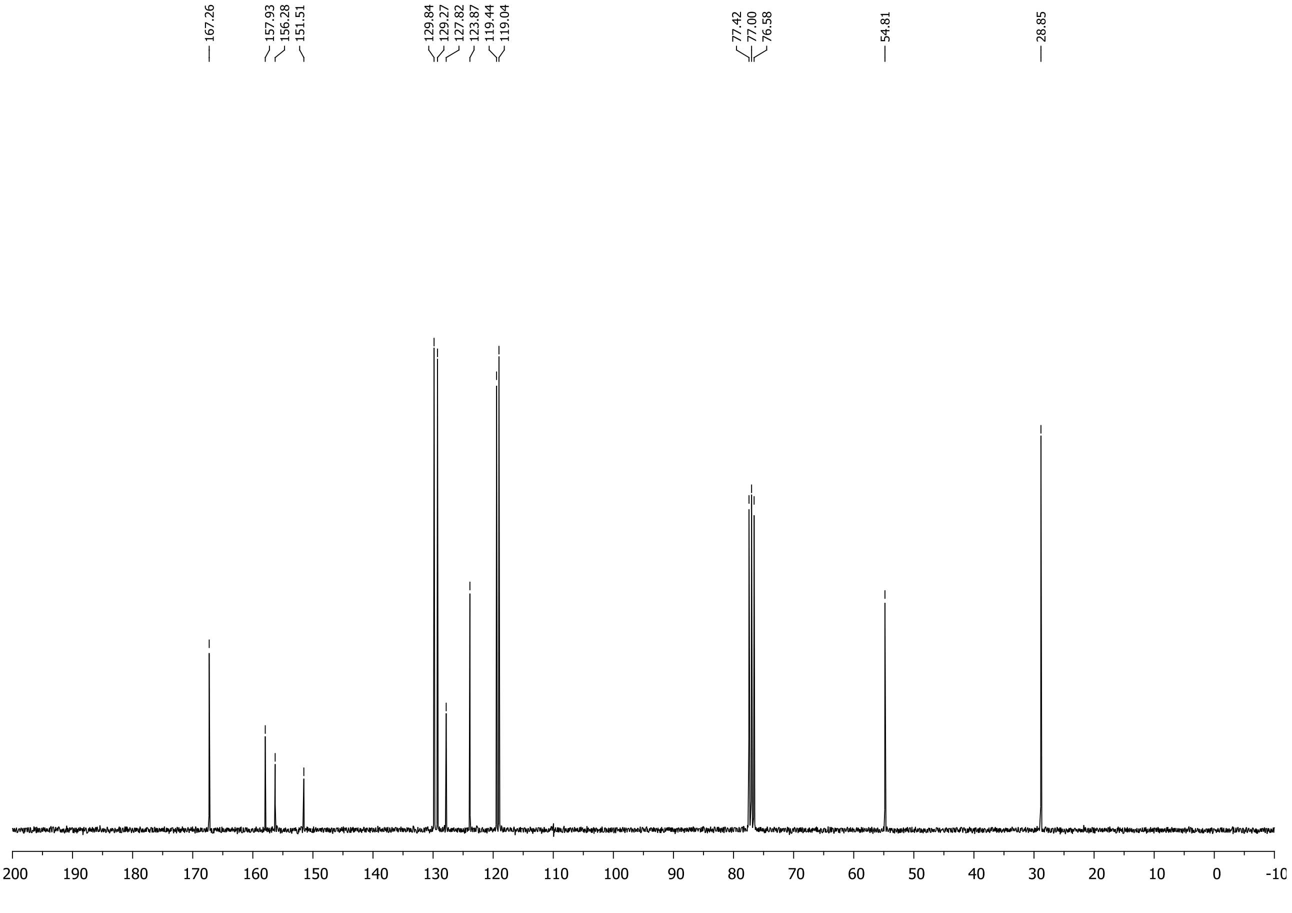
167.26
157.93
156.28
151.51
129.84
129.27
127.82
123.87
119.44
119.04
77.42
77.00
76.58
54.81
28.85
200 190 180 170 160 150 140 130 120 110 100 90 80 70 60 50 40 30 20 10 0

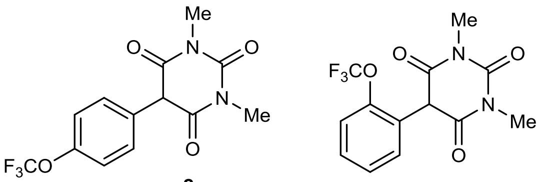

2e

4:1 mixture of regioisomers

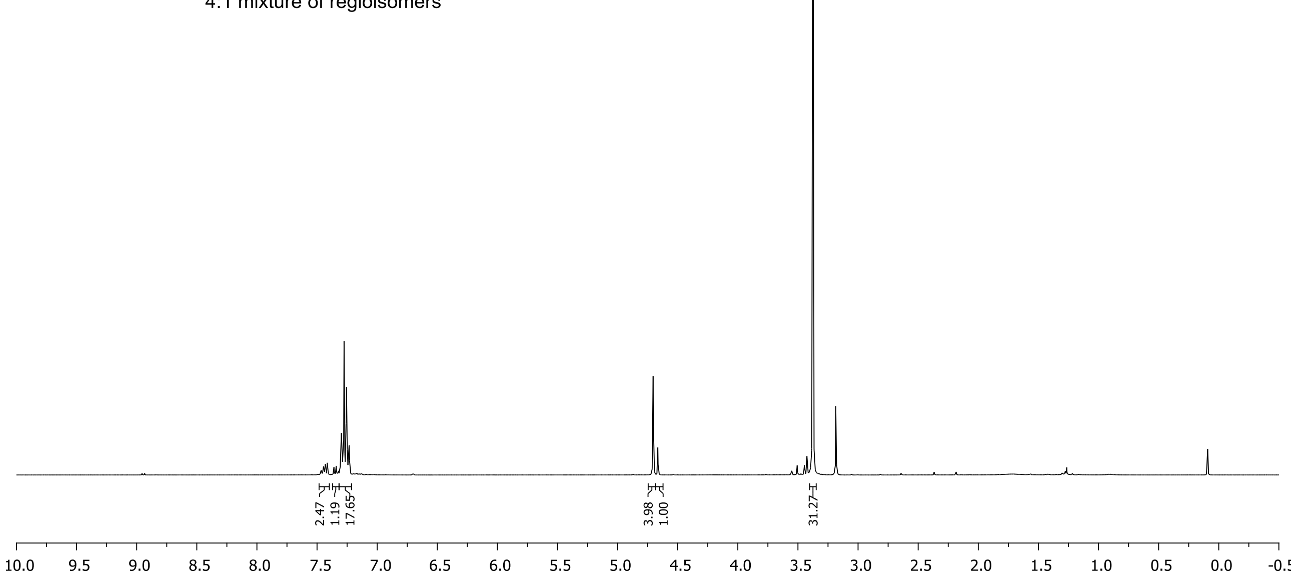

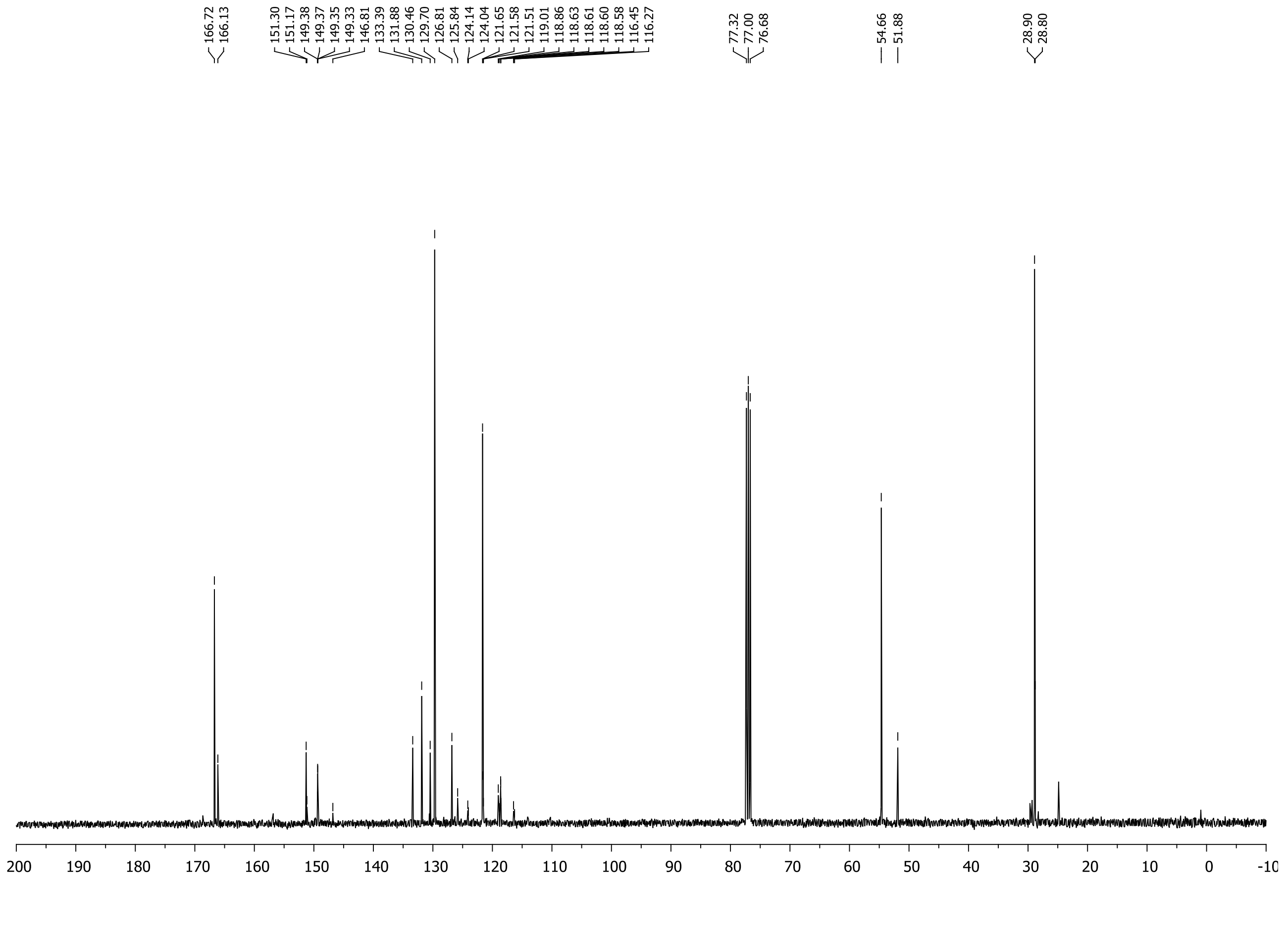

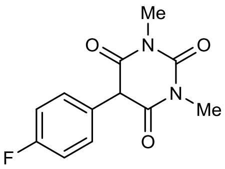
Me
O
N
O
N
Me
O
F

2f

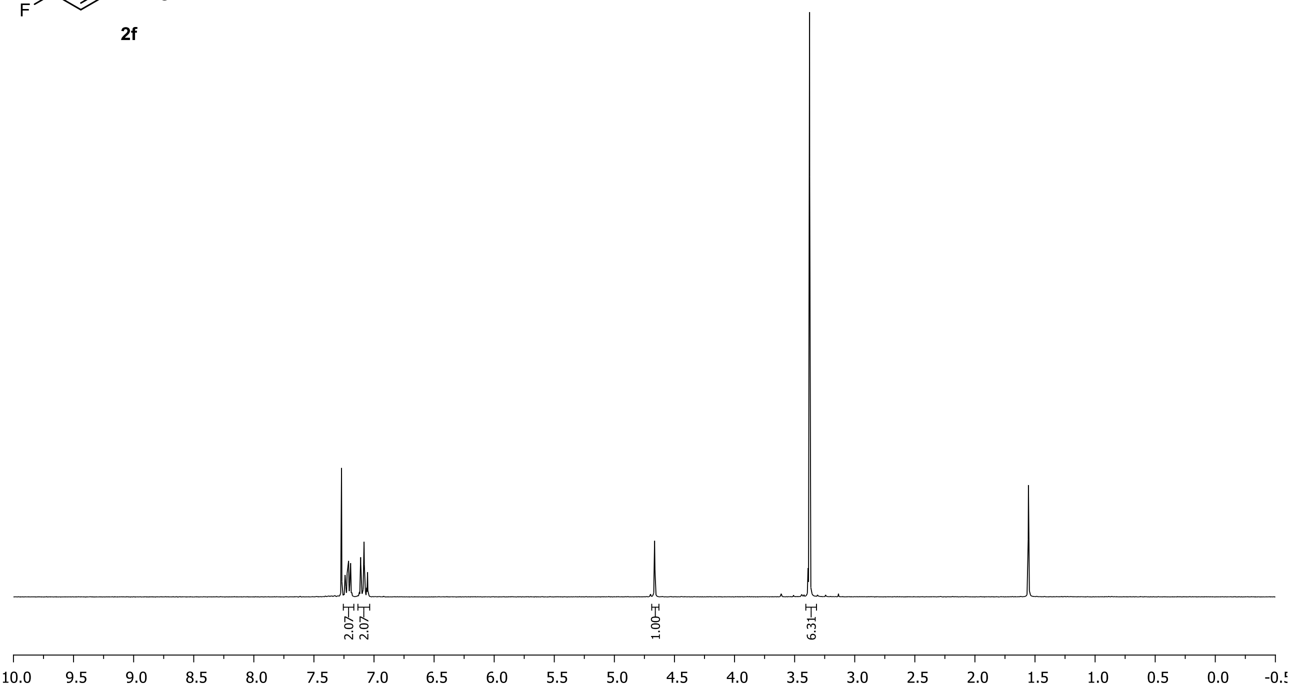
2.07
2.07
1.00
6.31
10.0 9.5 9.0 8.5 8.0 7.5 7.0 6.5 6.0 5.5 5.0 4.5 4.0 3.5 3.0 2.5 2.0 1.5 1.0 0.5 0.0

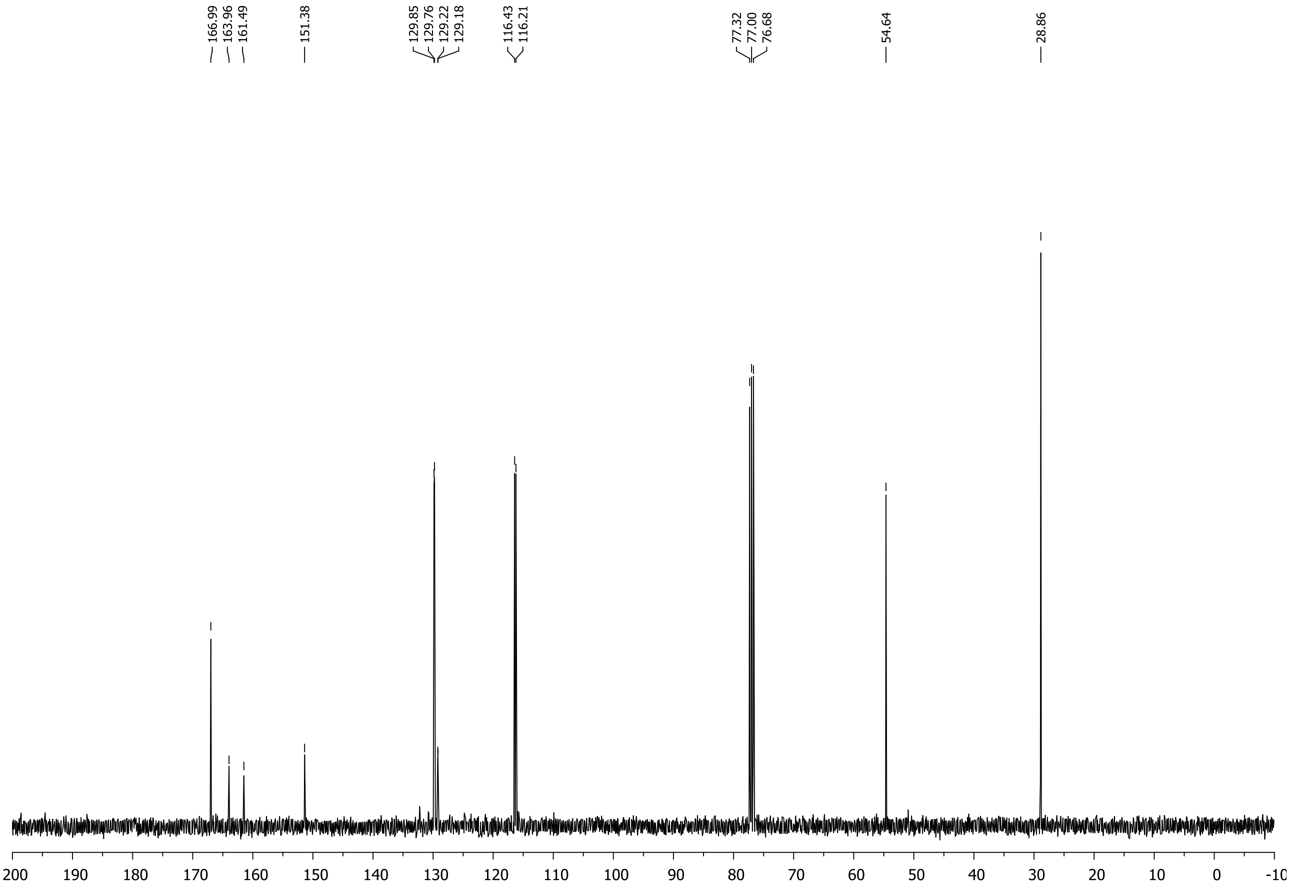
166.99
163.96
161.49
151.38
129.85
129.76
129.22
129.18
116.43
116.21
77.32
77.00
76.68
54.64
28.86
200 190 180 170 160 150 140 130 120 110 100 90 80 70 60 50 40 30 20 10 0

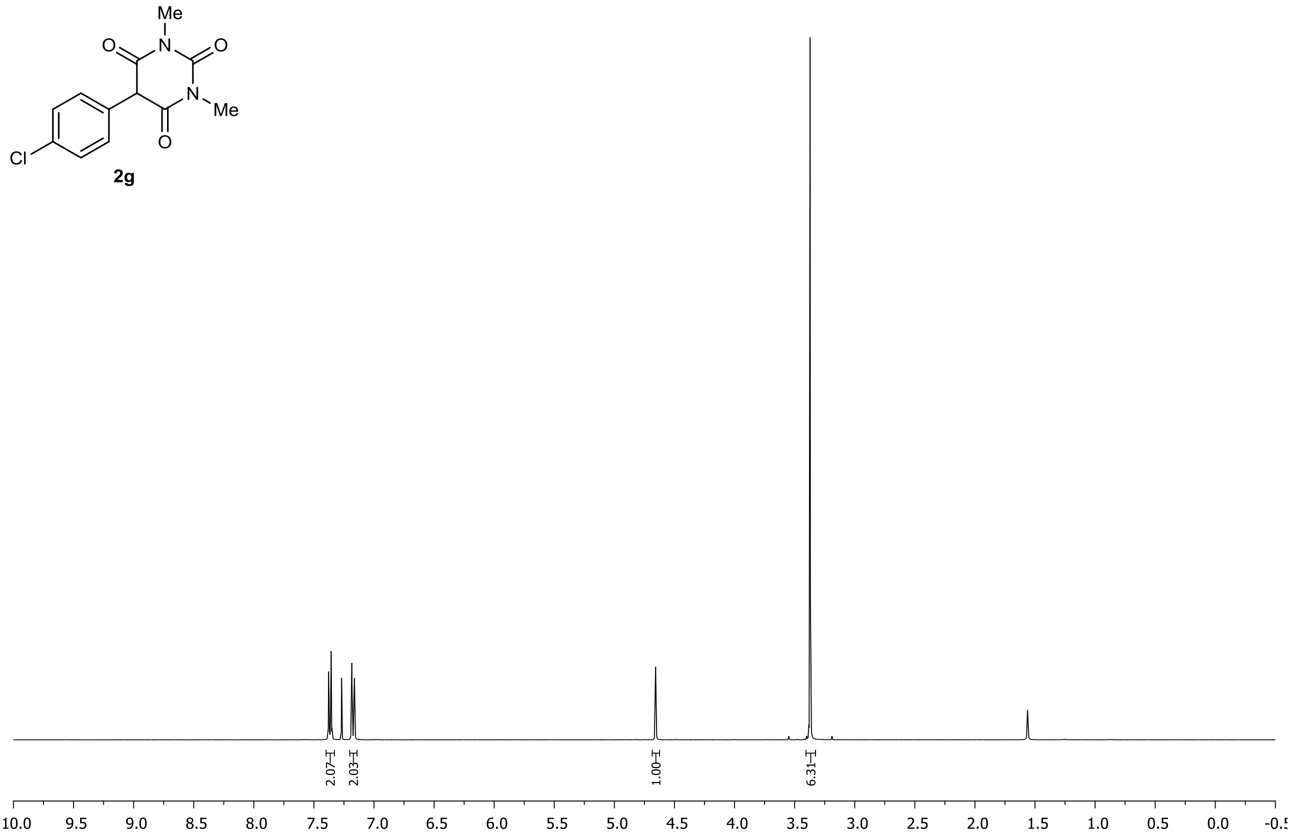
Me
O
N
O
N
Me
O
Cl
2.07
2.03
1.00
6.31
10.0 9.5 9.0 8.5 8.0 7.5 7.0 6.5 6.0 5.5 5.0 4.5 4.0 3.5 3.0 2.5 2.0 1.5 1.0 0.5 0.0

2g

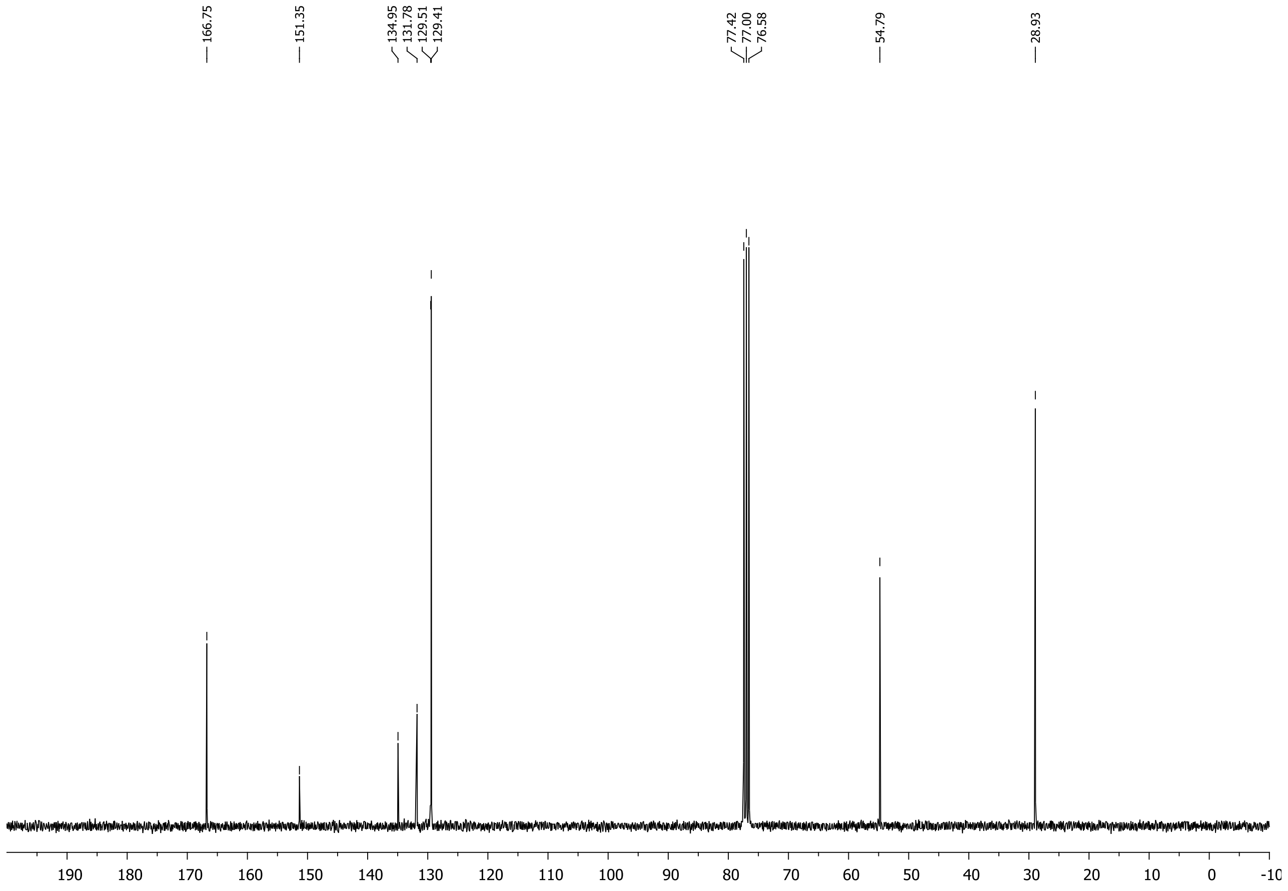
166.75
151.35
134.95
131.78
129.51
129.41
77.42
77.00
76.58
54.79
28.93
190 180 170 160 150 140 130 120 110 100 90 80 70 60 50 40 30 20 10 0

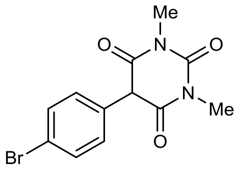
Me
O
N
O
N
Me
O
Br

2h

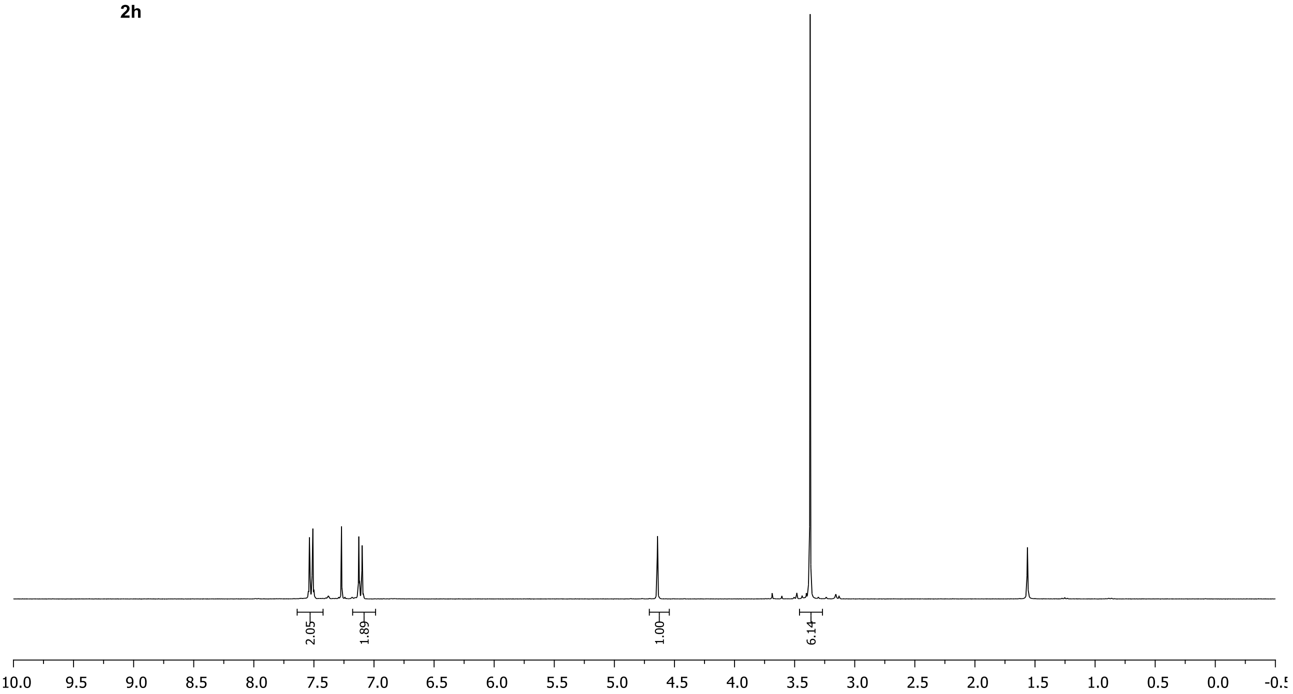
2.05
1.89
1.00
6.14
10.0
9.5
9.0
8.5
8.0
7.5
7.0
6.5
6.0
5.5
5.0
4.5
4.0
3.5
3.0
2.5
2.0
1.5
1.0
0.5
0.0

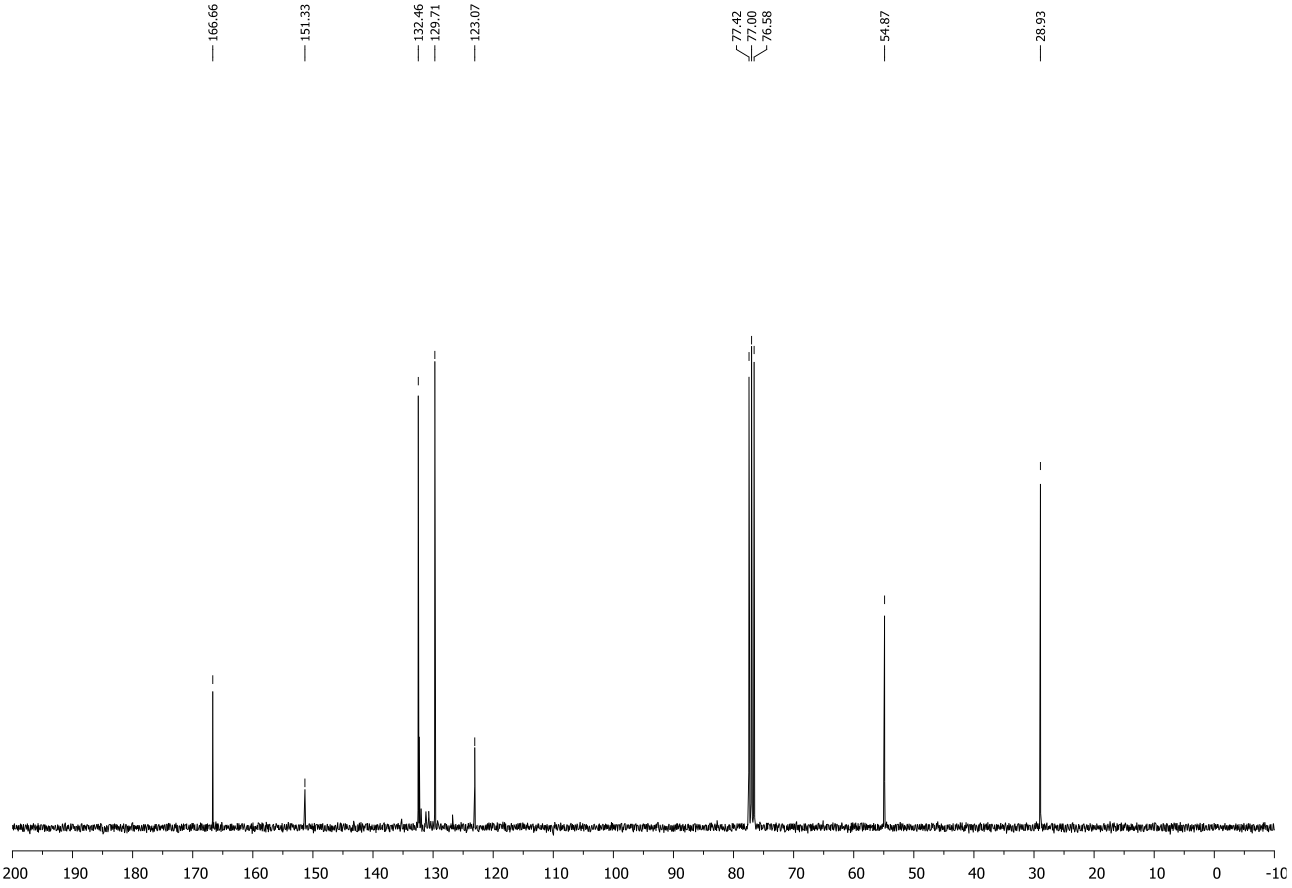
166.66
151.33
132.46
129.71
123.07
77.42
77.00
76.58
54.87
28.93
200
190
180
170
160
150
140
130
120
110
100
90
80
70
60
50
40
30
20
10
0

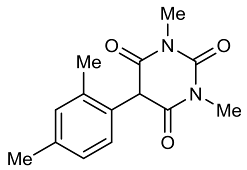
Me
O
N
O
Me
N
Me
O
Me

2i

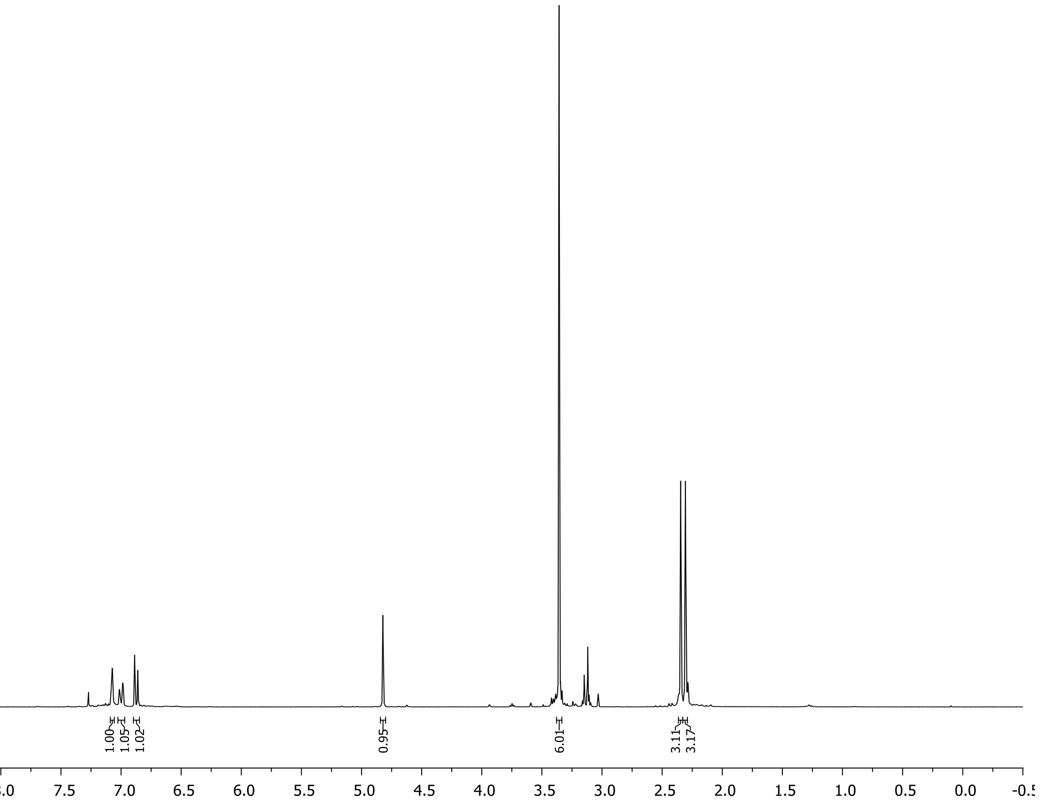
1.00
1.05
1.02
0.95
6.01
3.11
3.17
10.0 9.5 9.0 8.5 8.0 7.5 7.0 6.5 6.0 5.5 5.0 4.5 4.0 3.5 3.0 2.5 2.0 1.5 1.0 0.5 0.0

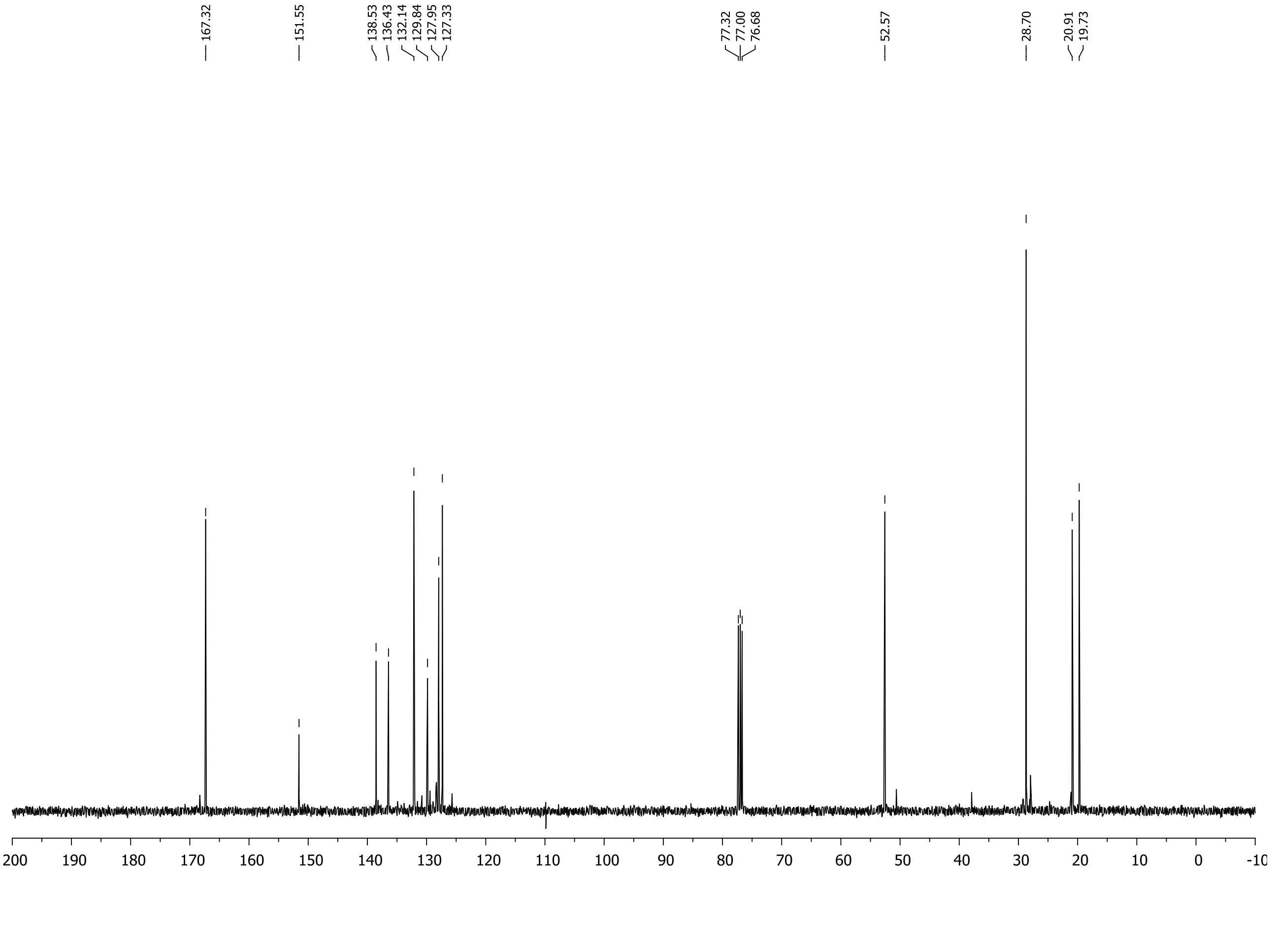
167.32
151.55
138.53
136.43
132.14
129.84
127.95
127.33
77.32
77.00
76.68
52.57
28.70
20.91
19.73
200 190 180 170 160 150 140 130 120 110 100 90 80 70 60 50 40 30 20 10 0

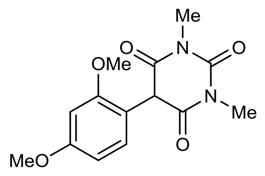
Me
O
N
O
OMe
N
Me
O
MeO

2j

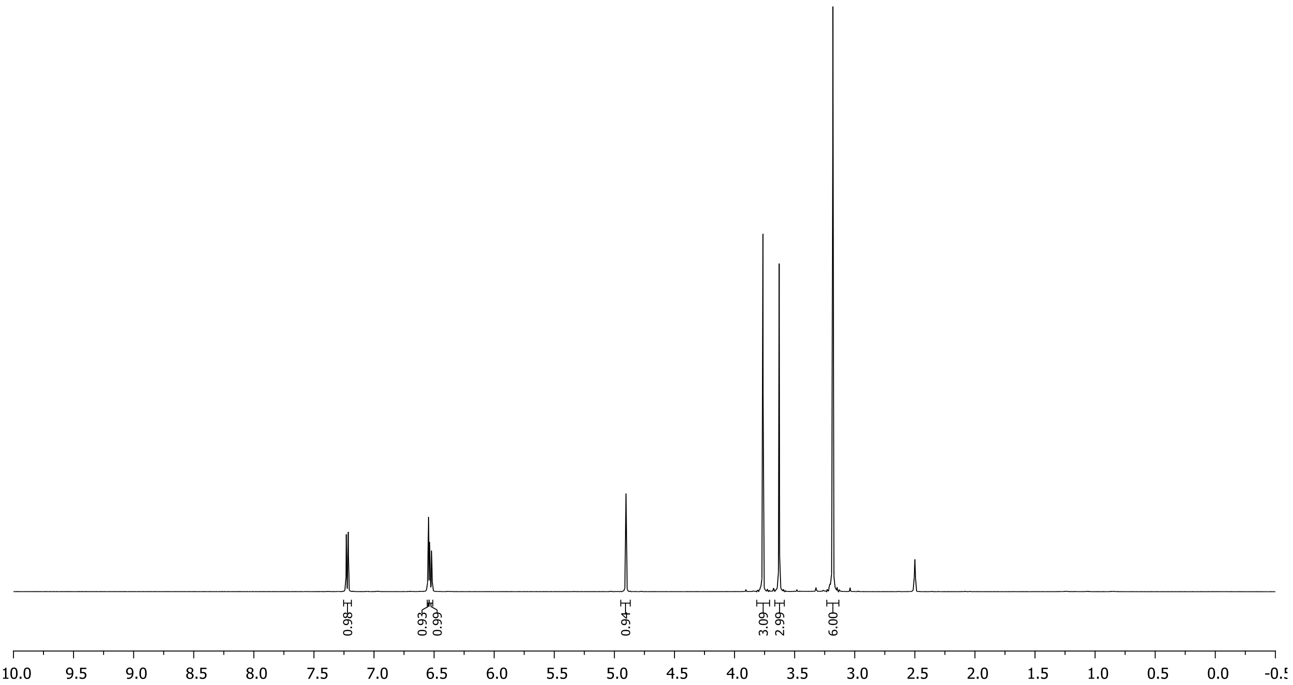
0.98
0.93
0.99
0.94
3.09
2.99
6.00
10.0 9.5 9.0 8.5 8.0 7.5 7.0 6.5 6.0 5.5 5.0 4.5 4.0 3.5 3.0 2.5 2.0 1.5 1.0 0.5 0.0

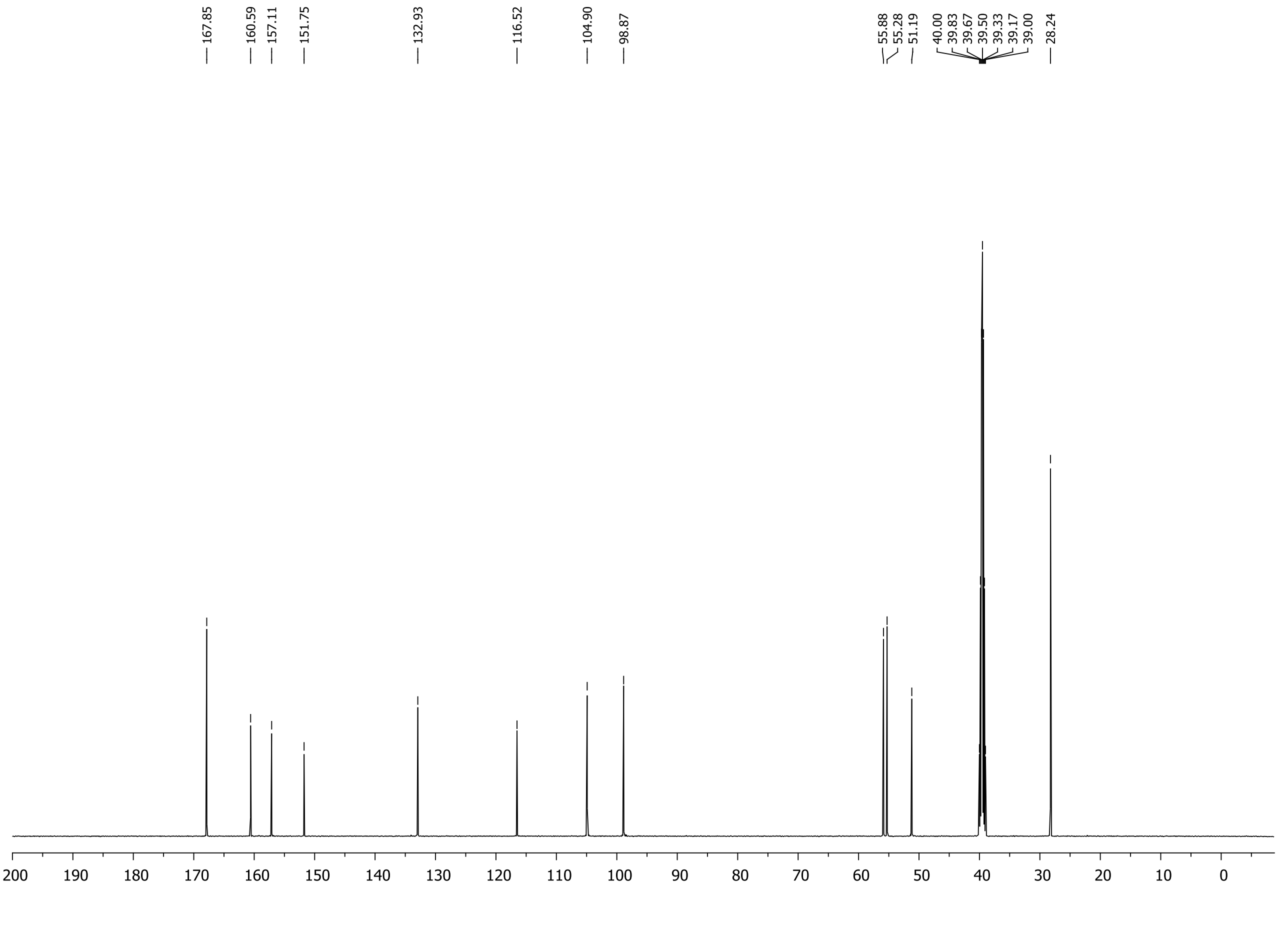
167.85
160.59
157.11
151.75
132.93
116.52
104.90
98.87
55.88
55.28
51.19
40.00
39.83
39.67
39.50
39.33
39.17
39.00
28.24
200 190 180 170 160 150 140 130 120 110 100 90 80 70 60 50 40 30 20 10 0

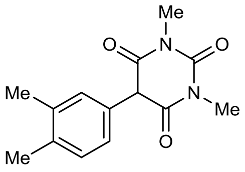
Me
O
N
O
Me
N
Me
Me
O

2ka

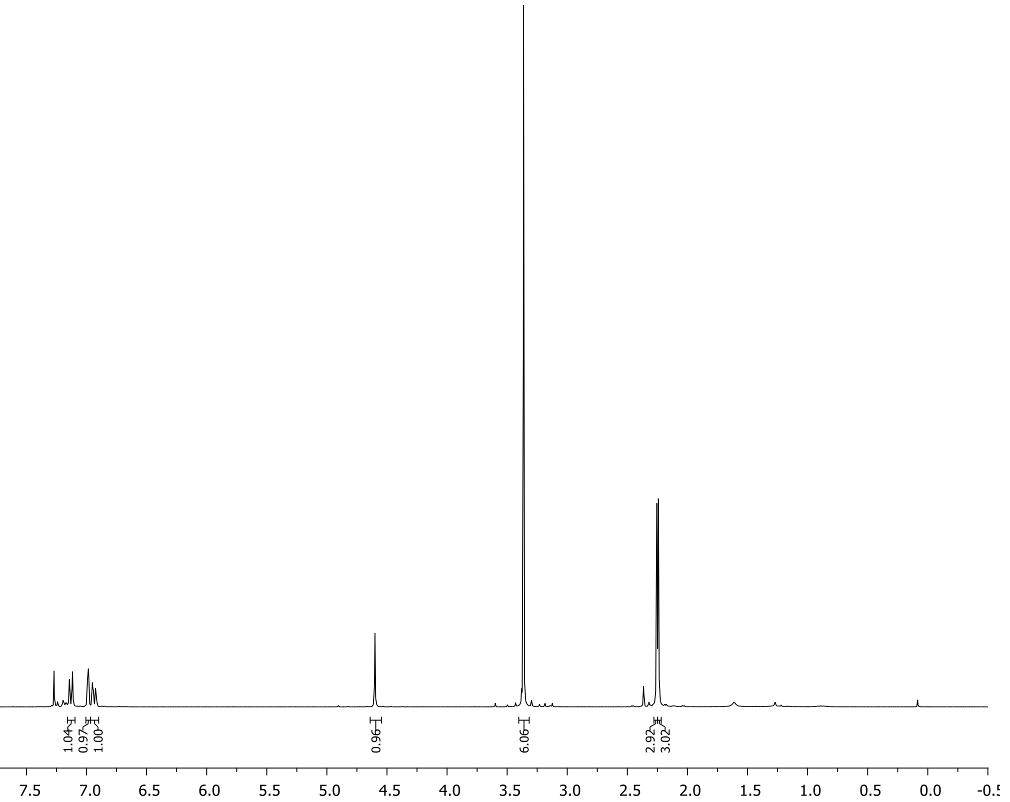
1.04
0.97
1.00
0.96
6.06
2.92
3.02
10.0
9.5
9.0
8.5
8.0
7.5
7.0
6.5
6.0
5.5
5.0
4.5
4.0
3.5
3.0
2.5
2.0
1.5
1.0
0.5
0.0

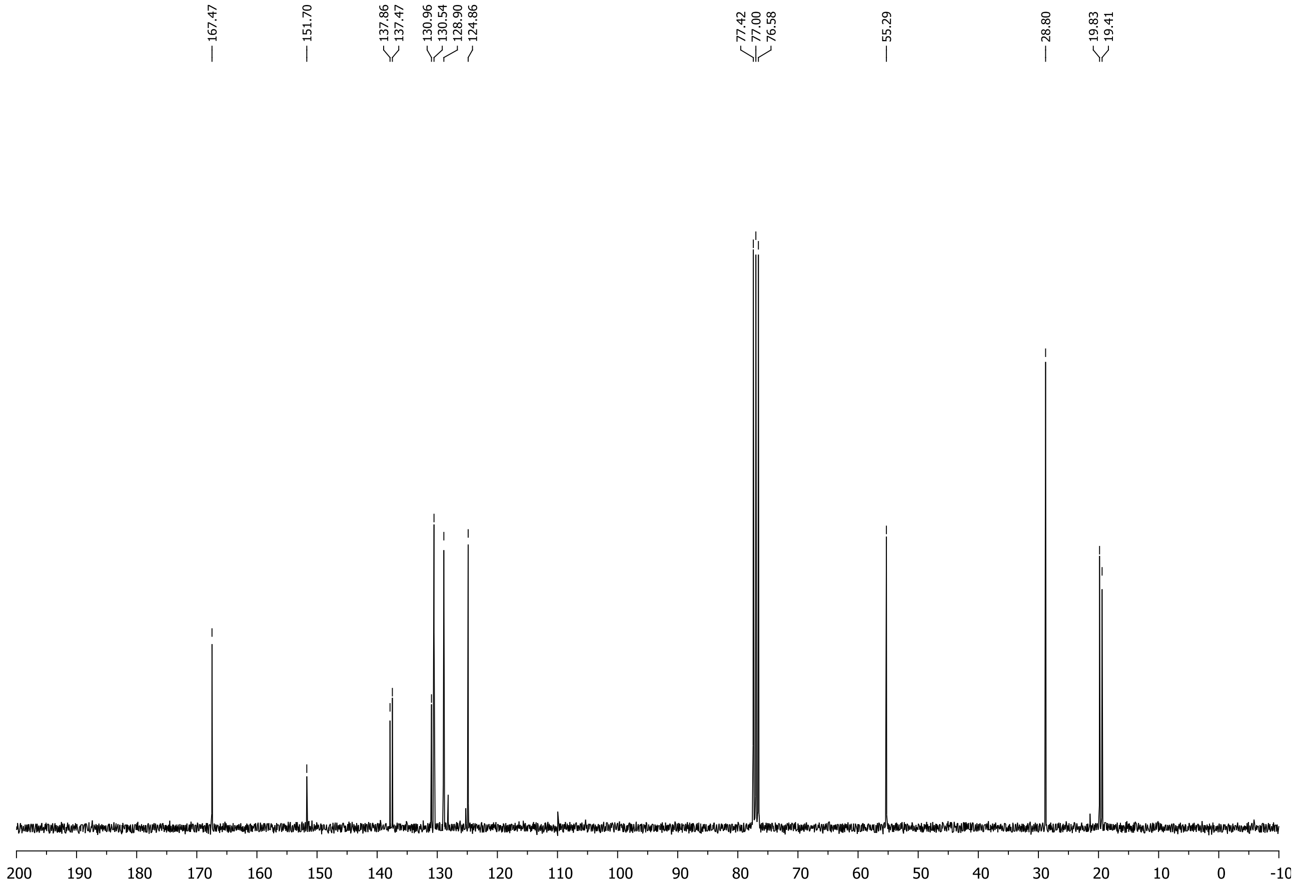
167.47
151.70
137.86
137.47
130.96
130.54
128.90
124.86
77.42
77.00
76.58
55.29
28.80
19.83
19.41
200
190
180
170
160
150
140
130
120
110
100
90
80
70
60
50
40
30
20
10
0

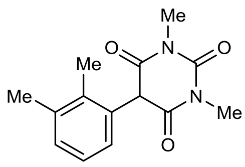
Me
O
N
O
Me
N
Me
Me
O

2kb

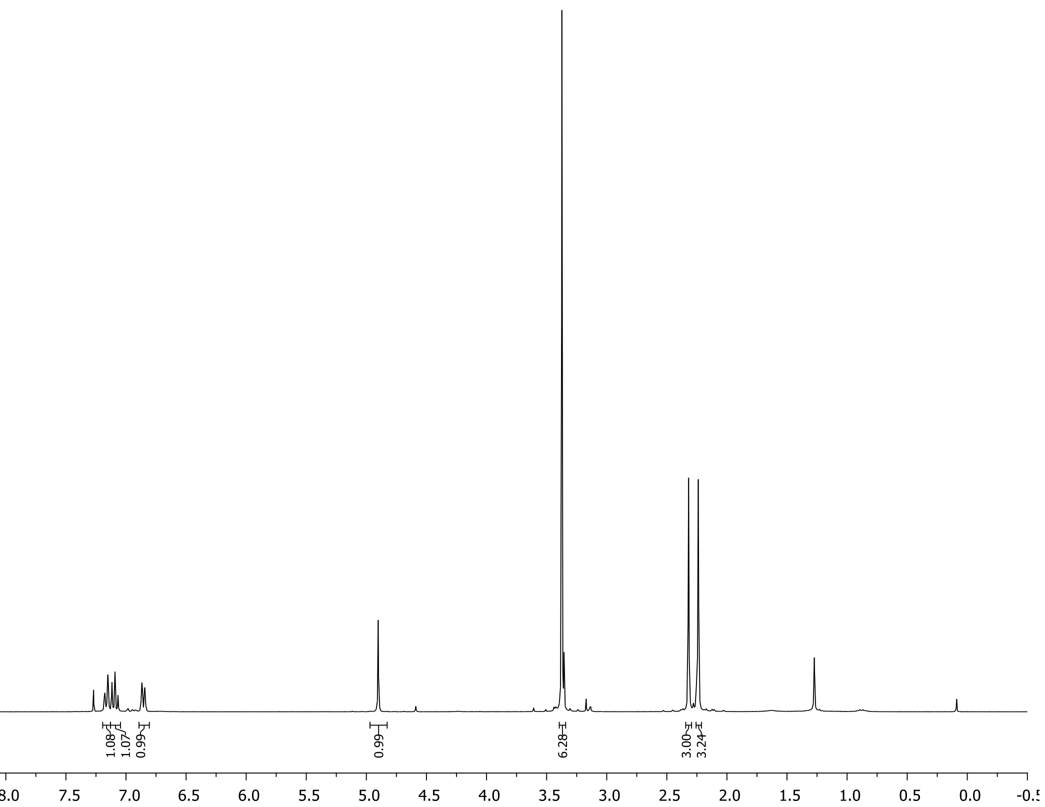
1.08
1.07
0.99
0.99
6.28
3.00
3.24
10.0 9.5 9.0 8.5 8.0 7.5 7.0 6.5 6.0 5.5 5.0 4.5 4.0 3.5 3.0 2.5 2.0 1.5 1.0 0.5 0.0

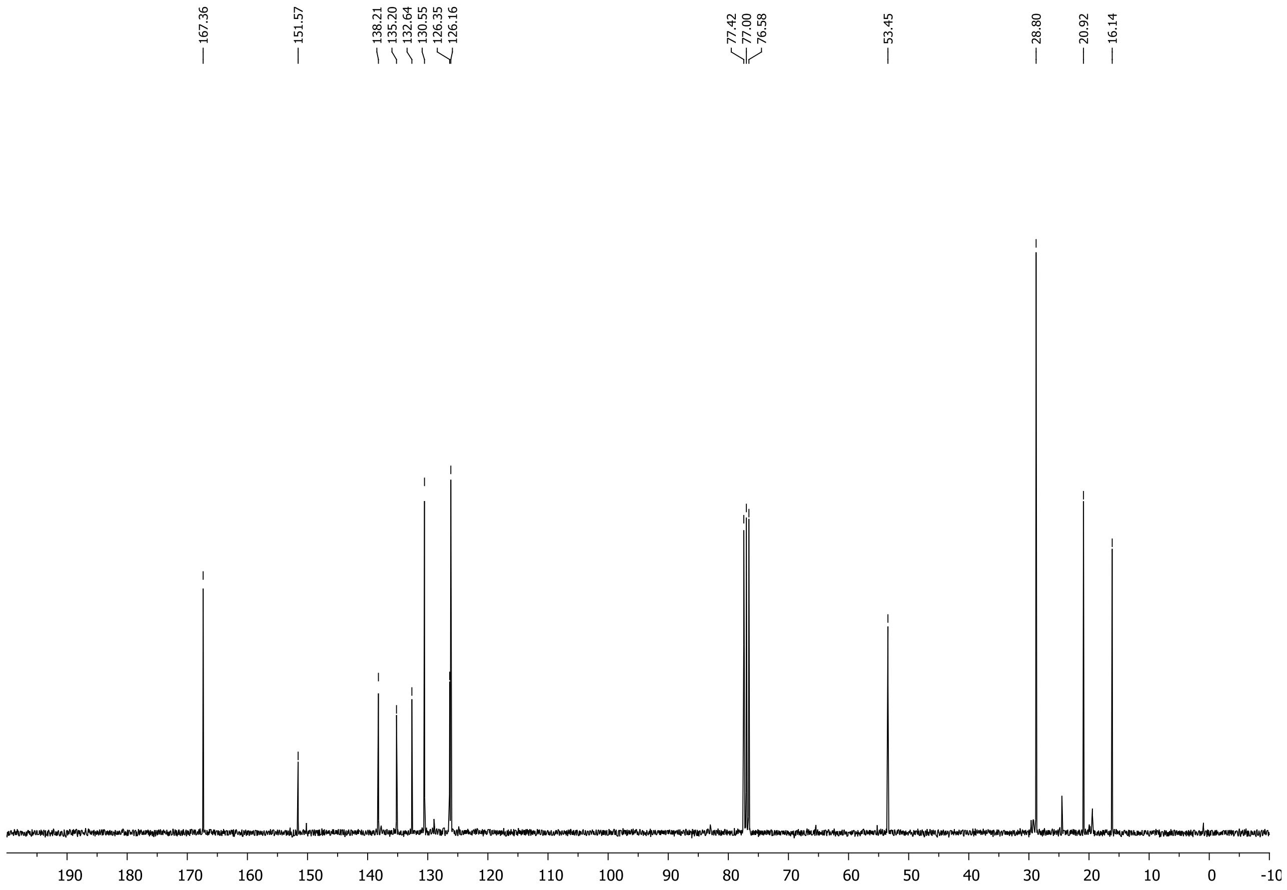
167.36
151.57
138.21
135.20
132.64
130.55
126.35
126.16
77.42
77.00
76.58
53.45
28.80
20.92
16.14
190 180 170 160 150 140 130 120 110 100 90 80 70 60 50 40 30 20 10 0

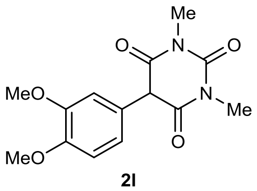
Me
O
N
O
MeO
N
Me
MeO
O

2l

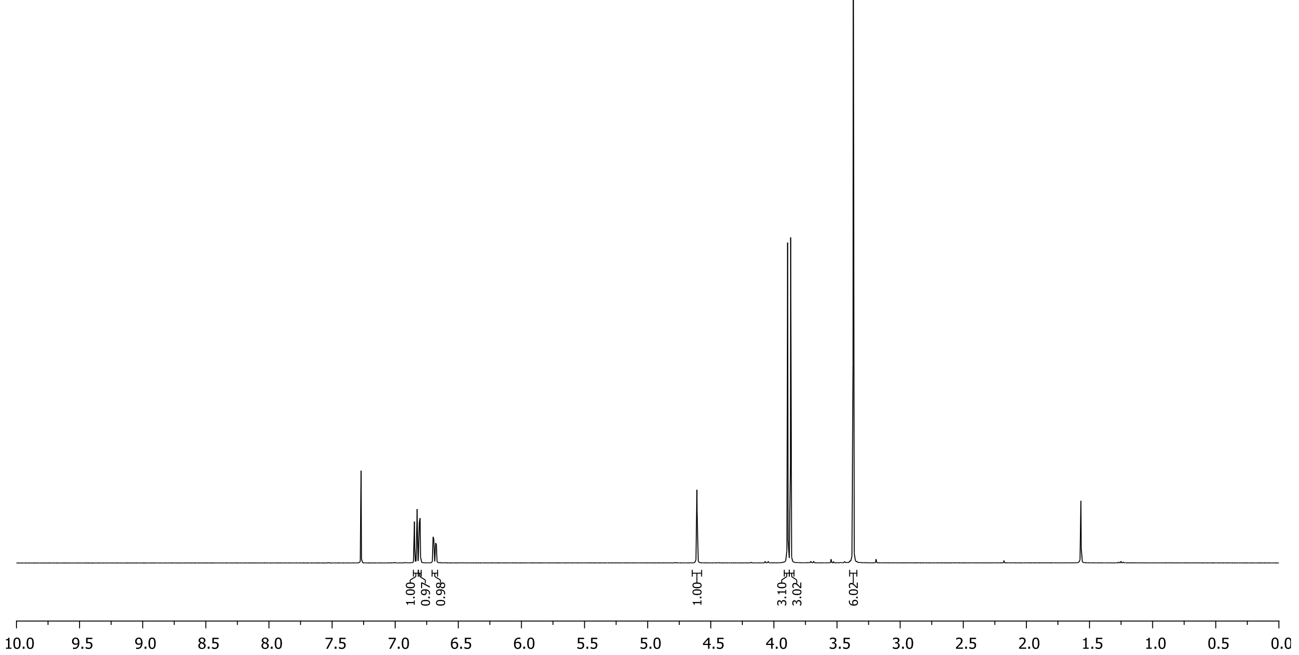
1.00
0.97
0.98
1.00
3.10
3.02
6.02
10.0 9.5 9.0 8.5 8.0 7.5 7.0 6.5 6.0 5.5 5.0 4.5 4.0 3.5 3.0 2.5 2.0 1.5 1.0 0.5 0.0

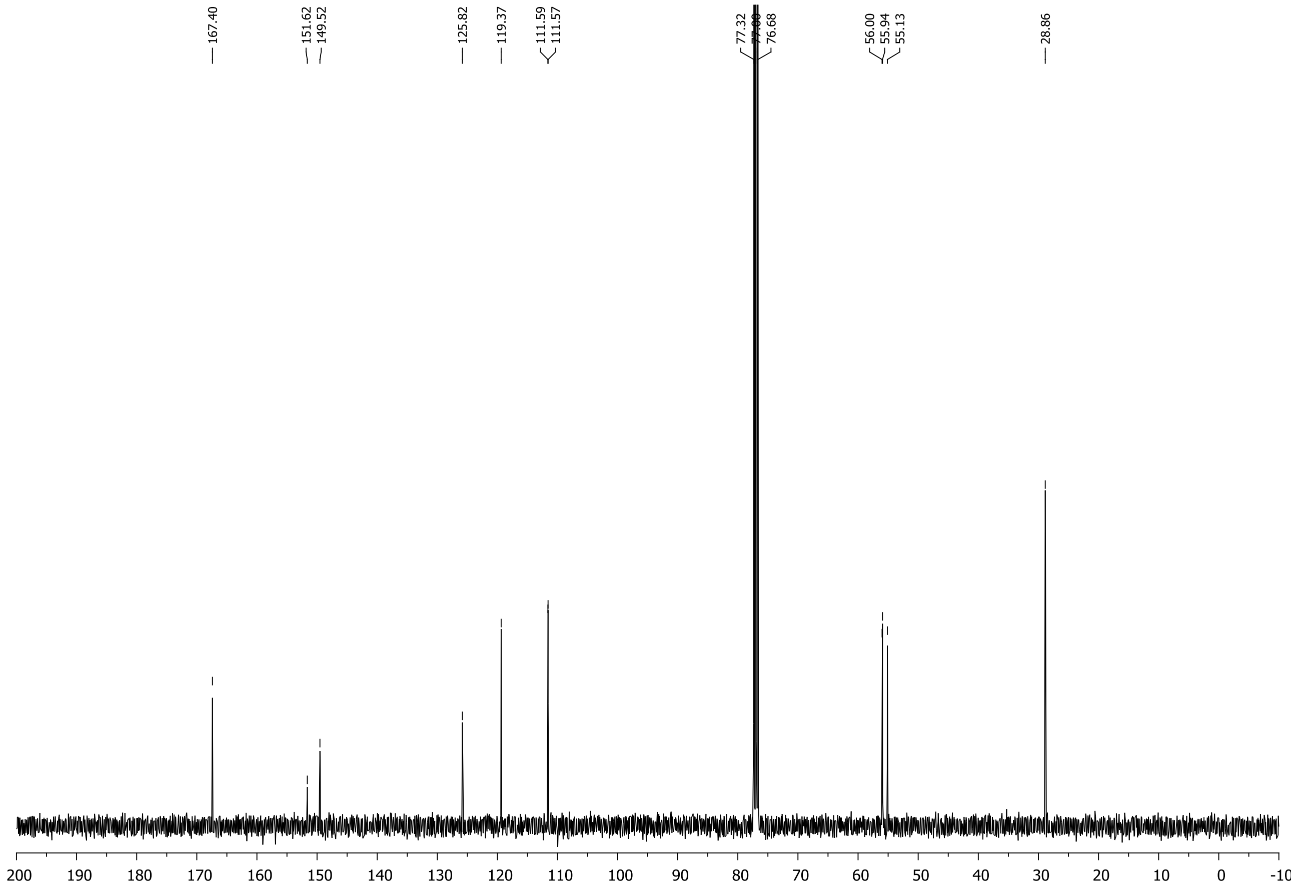
167.40
151.62
149.52
125.82
119.37
111.59
111.57
77.32
76.68
56.00
55.94
55.13
28.86
200 190 180 170 160 150 140 130 120 110 100 90 80 70 60 50 40 30 20 10 0 -10

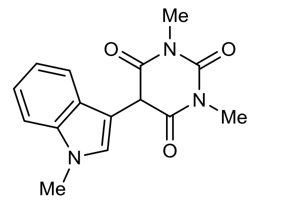

3
(^{1}H NMR spectrum run at 70 °C)

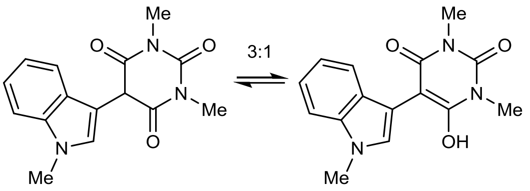

3
(^{13}C NMR spectrum run at RT)

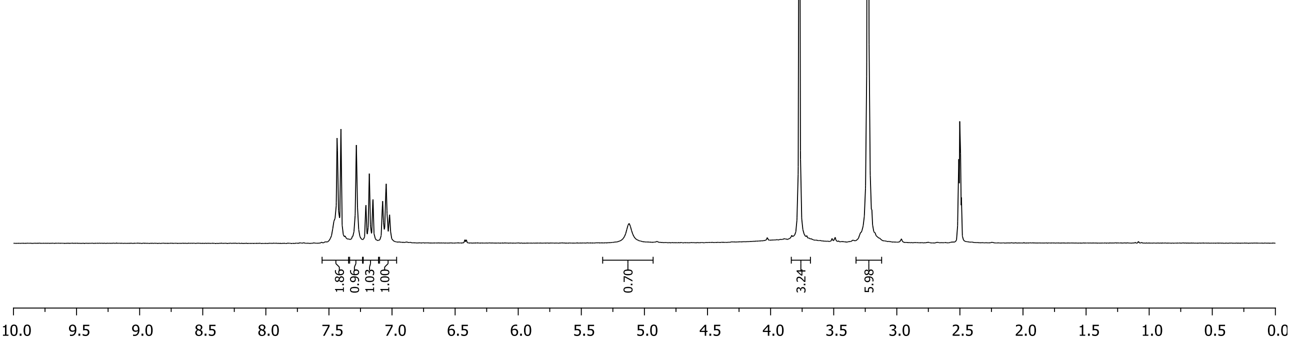

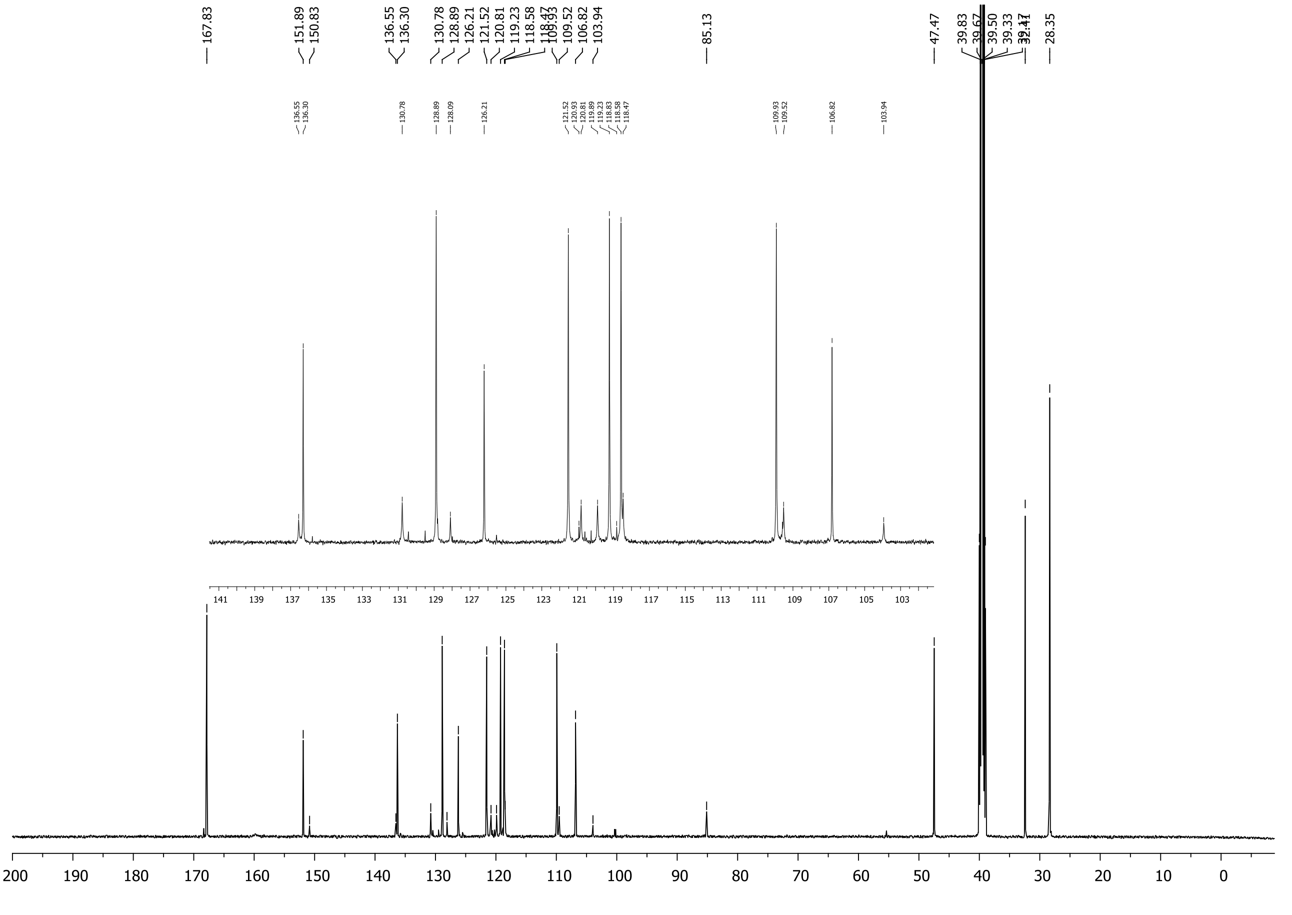

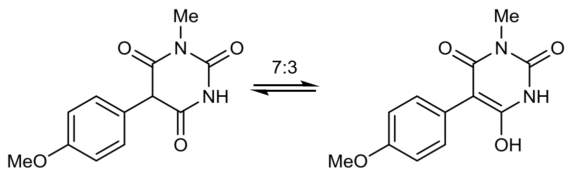
Me
O
N
O
NH
O
MeO
7:3
Me
O
N
O
NH
OH
MeO

4a

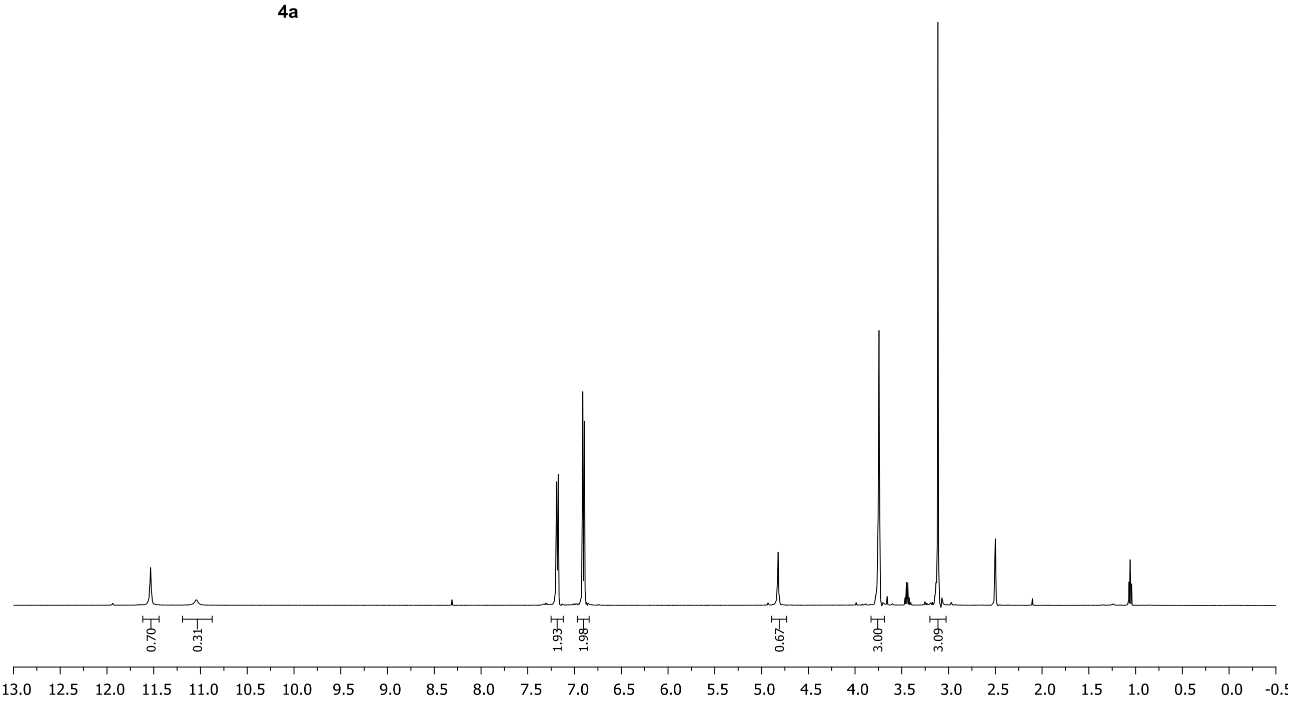
0.70
0.31
1.93
1.98
0.67
3.00
3.09
13.0 12.5 12.0 11.5 11.0 10.5 10.0 9.5 9.0 8.5 8.0 7.5 7.0 6.5 6.0 5.5 5.0 4.5 4.0 3.5 3.0 2.5 2.0 1.5 1.0 0.5 0.0

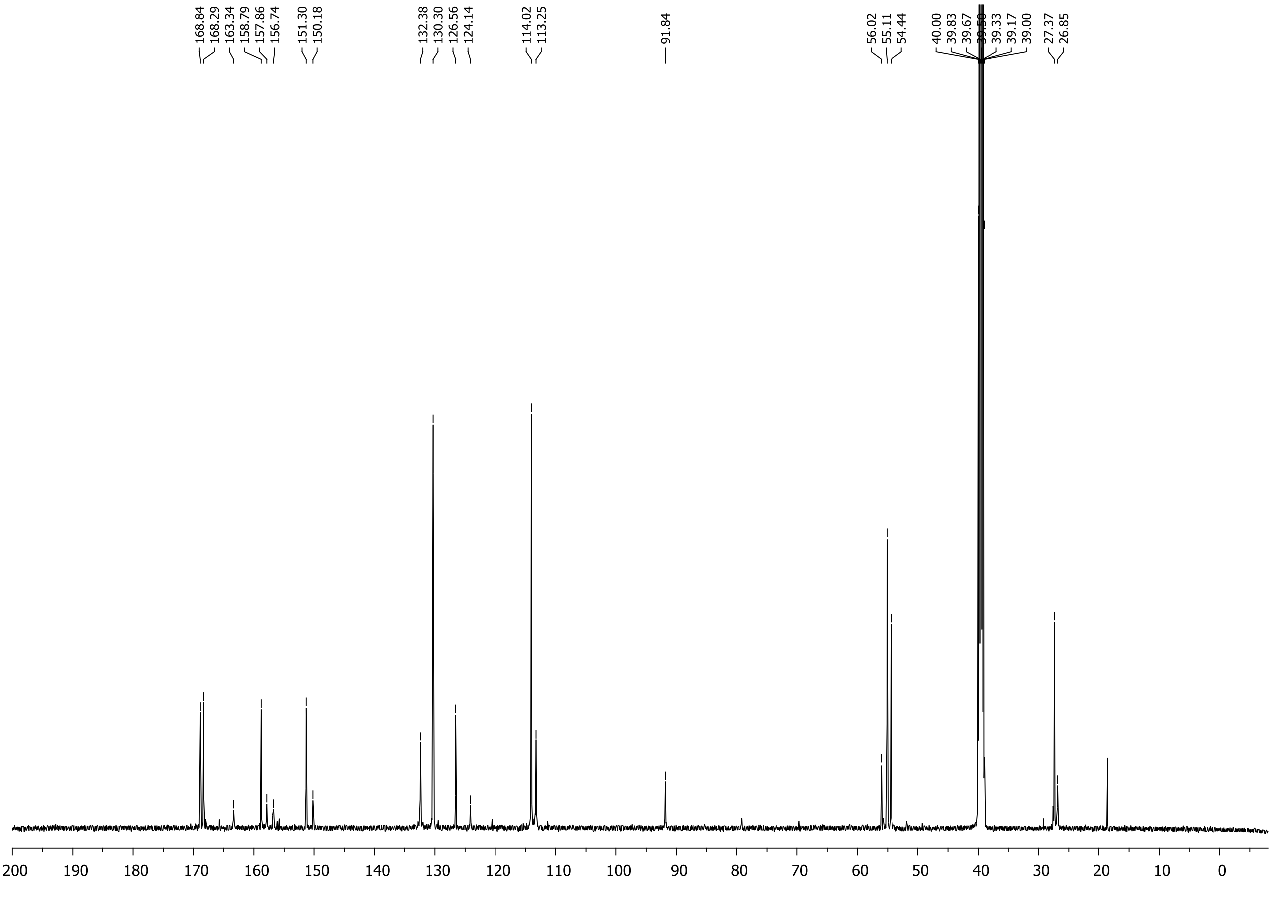
168.84
168.29
163.34
158.79
157.86
156.74
151.30
150.18
132.38
130.30
126.56
124.14
114.02
113.25
91.84
56.02
55.11
54.44
40.00
39.83
39.67
39.33
39.17
39.00
27.37
26.85
200 190 180 170 160 150 140 130 120 110 100 90 80 70 60 50 40 30 20 10 0

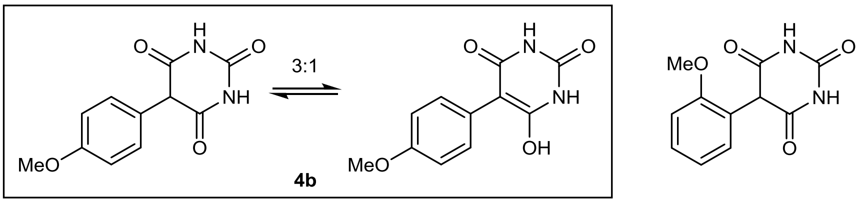

9:1 mixture of regioisomers
(enol form of minor regioisomer could
not be observed)

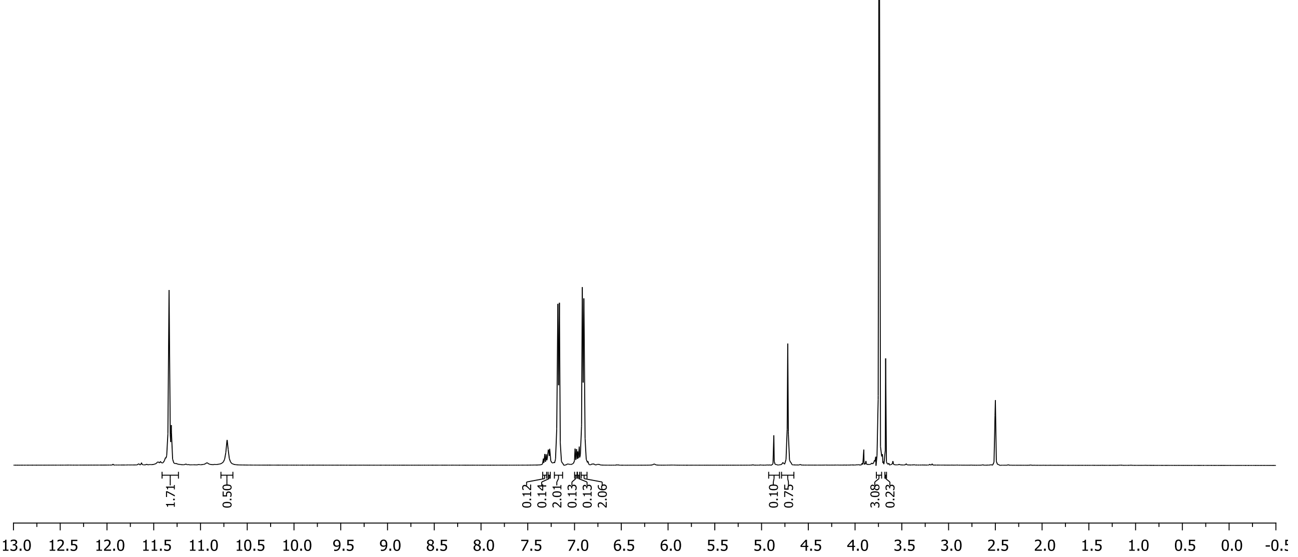

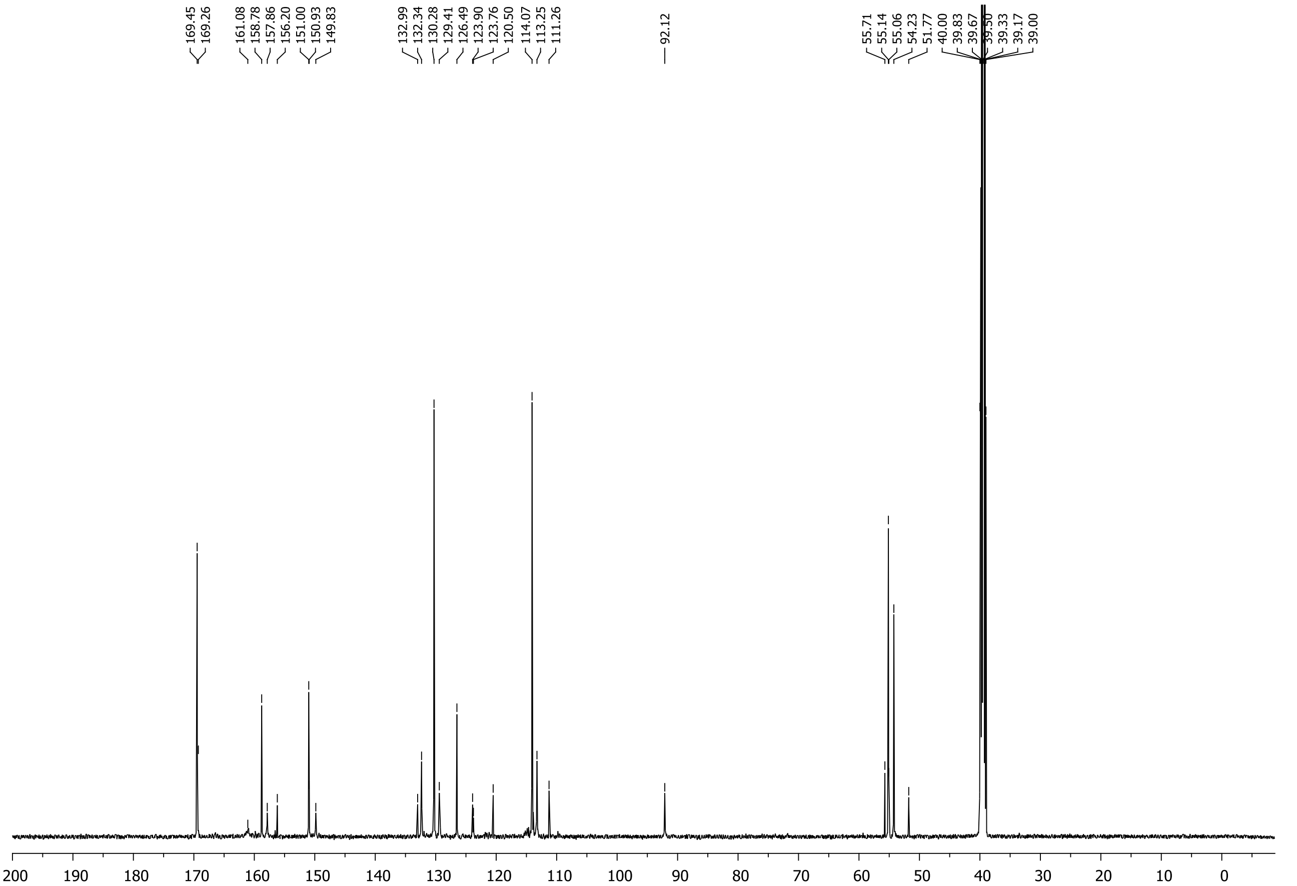

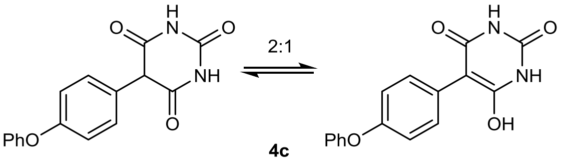
2:1
PhO
4c
PhO
OH

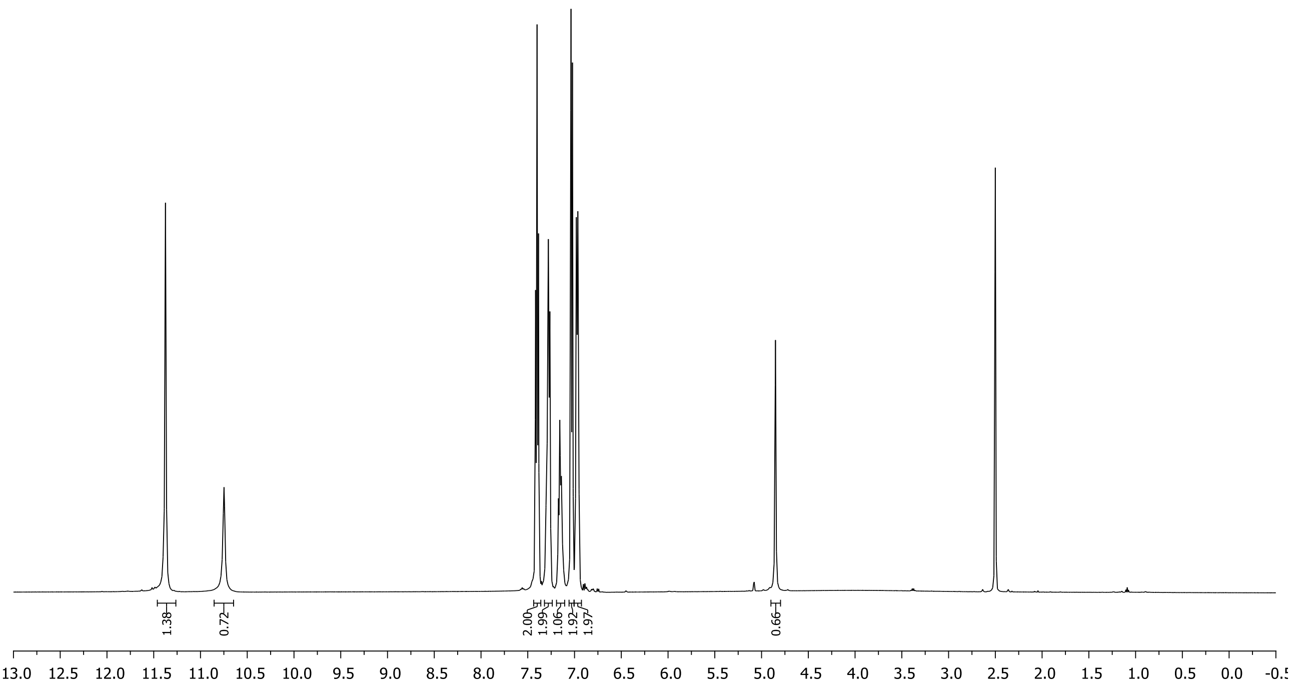
1.38
0.72
2.00
1.99
1.06
1.92
1.97
0.66
13.0 12.5 12.0 11.5 11.0 10.5 10.0 9.5 9.0 8.5 8.0 7.5 7.0 6.5 6.0 5.5 5.0 4.5 4.0 3.5 3.0 2.5 2.0 1.5 1.0 0.5 0.0

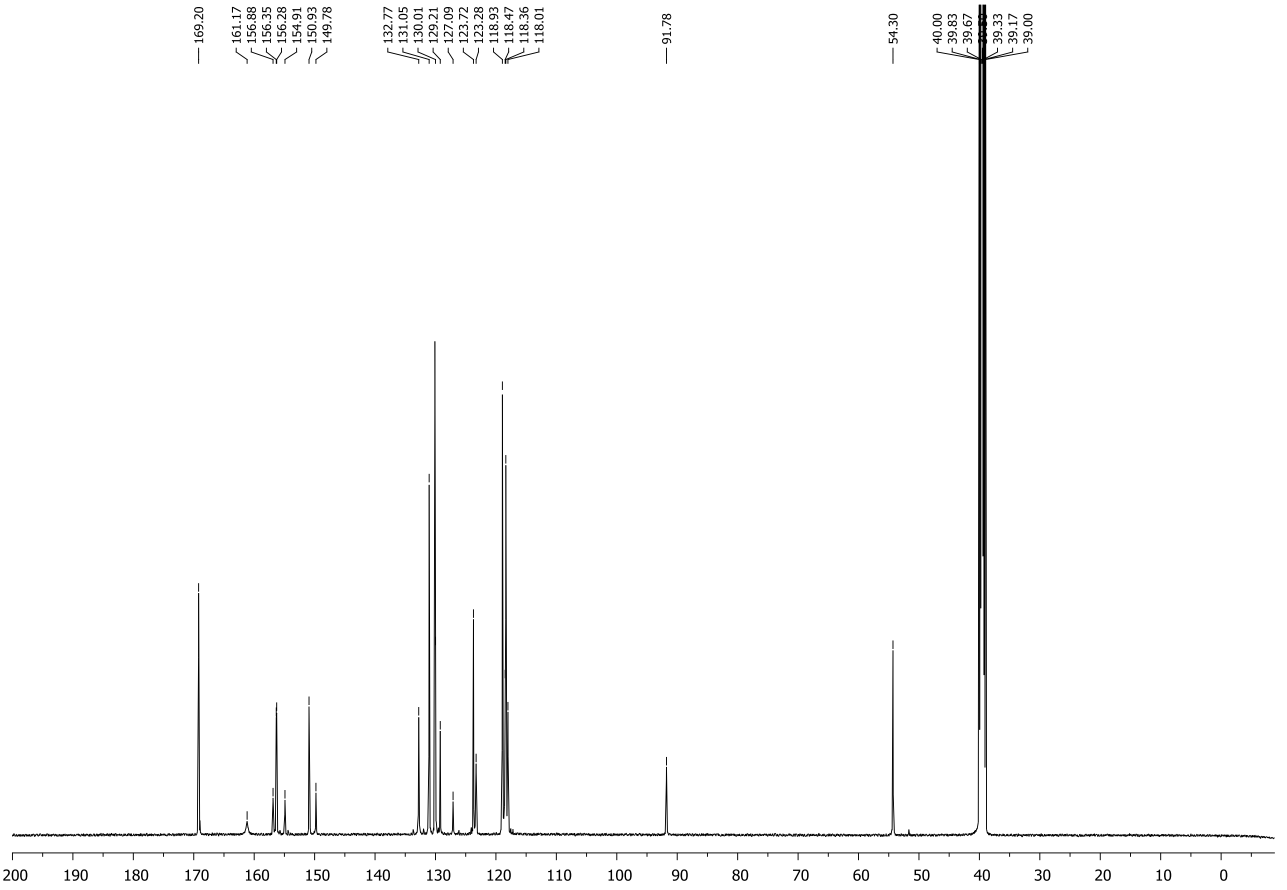
169.20
161.17
156.88
156.35
156.28
154.91
150.93
149.78
132.77
131.05
130.01
129.21
127.09
123.72
123.28
118.93
118.47
118.36
118.01
91.78
54.30
40.00
39.83
39.67
39.33
39.17
39.00
200 190 180 170 160 150 140 130 120 110 100 90 80 70 60 50 40 30 20 10 0

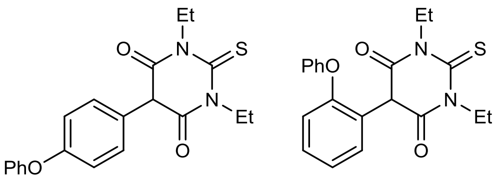

4d

6:1 mixture of regioisomers

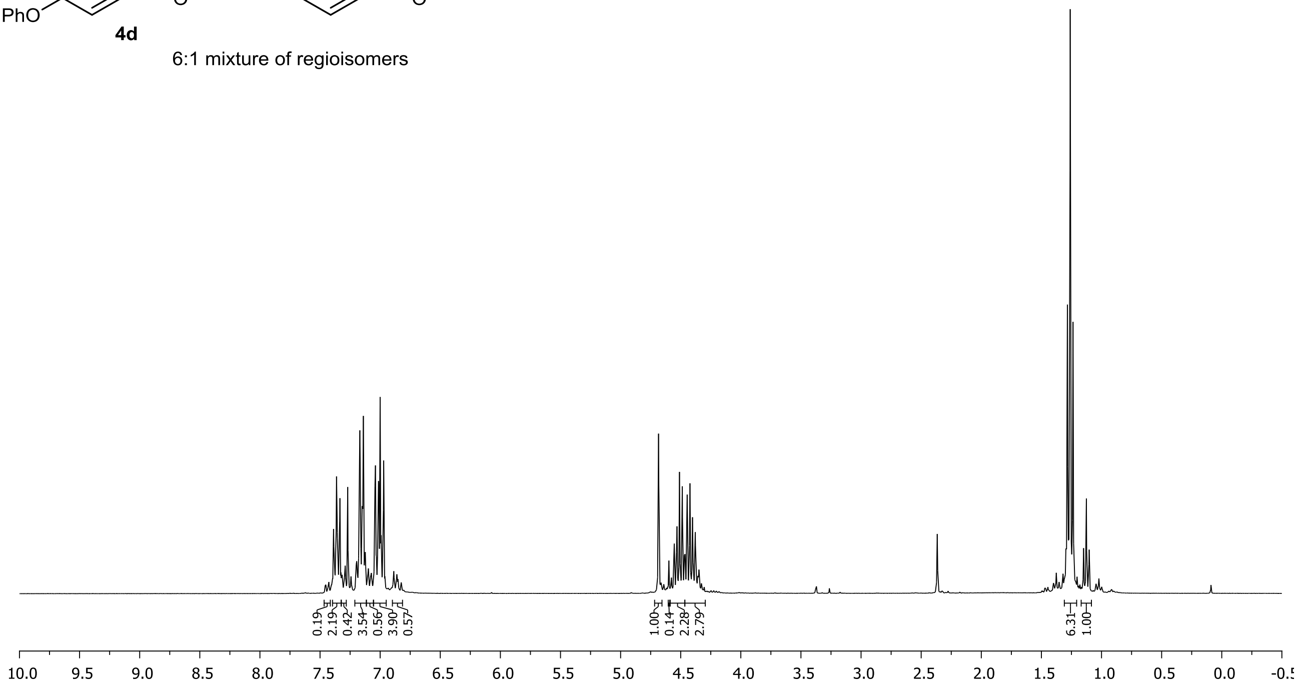

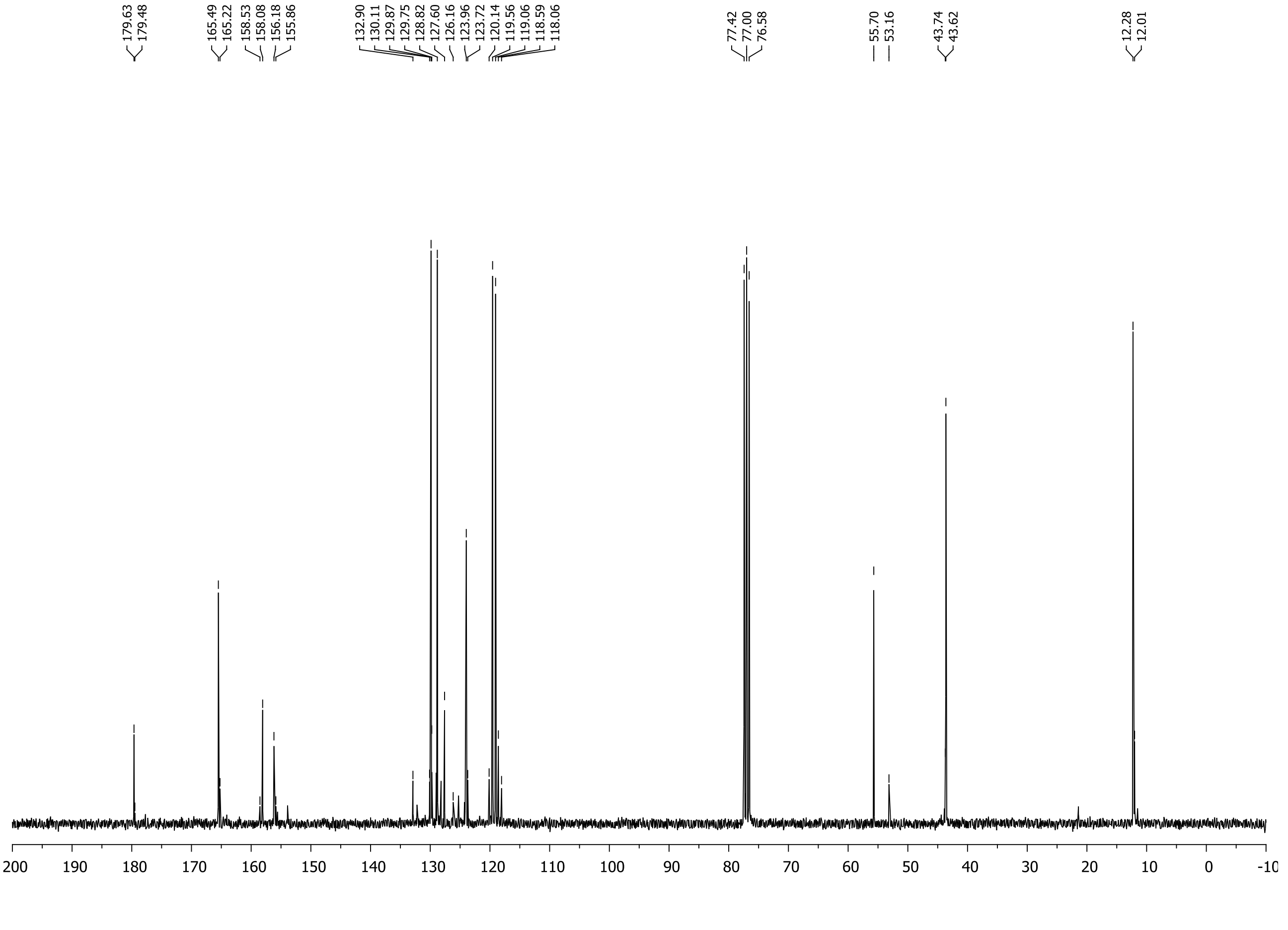

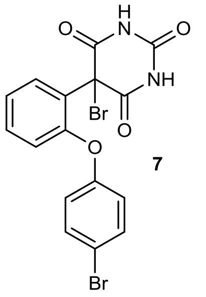

7

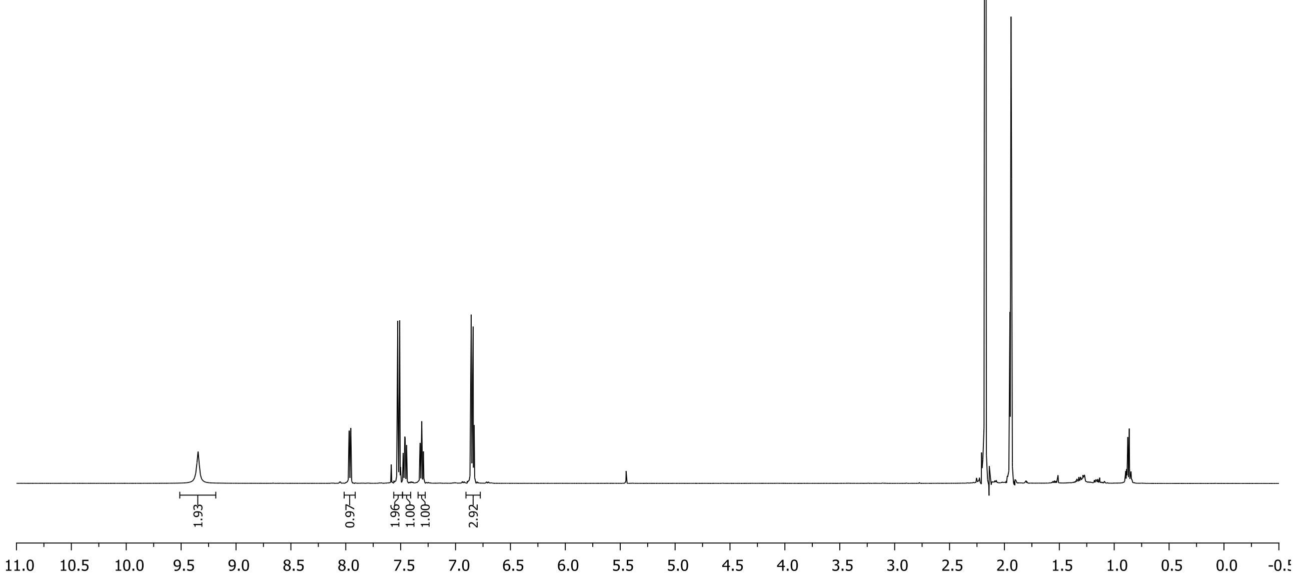
1.93
0.97
1.96
1.00
1.00
2.92
11.0 10.5 10.0 9.5 9.0 8.5 8.0 7.5 7.0 6.5 6.0 5.5 5.0 4.5 4.0 3.5 3.0 2.5 2.0 1.5 1.0 0.5 0.0

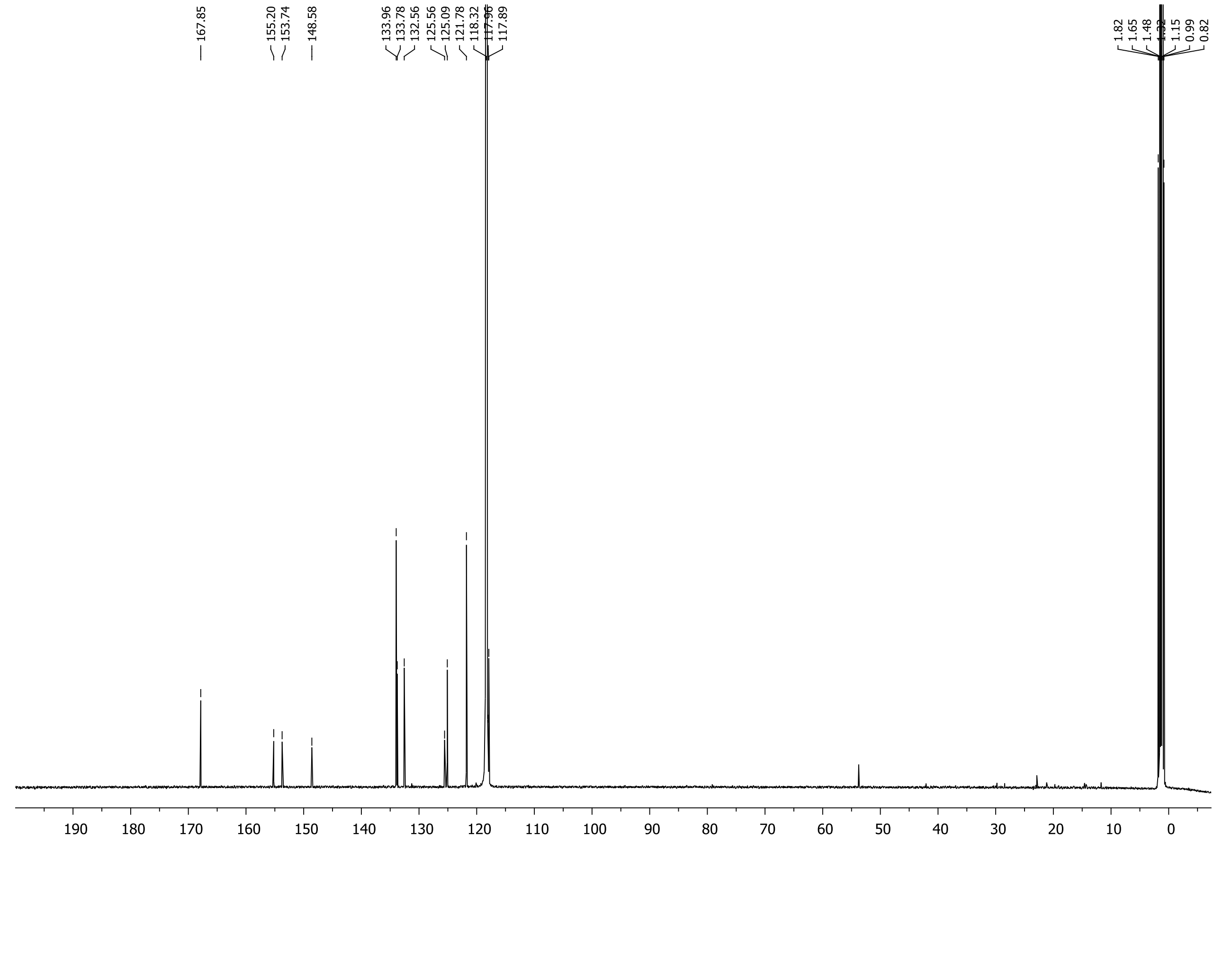
167.85
155.20
153.74
148.58
133.96
133.78
132.56
125.56
125.09
121.78
118.32
117.89
1.82
1.65
1.48
1.15
0.99
0.82
190 180 170 160 150 140 130 120 110 100 90 80 70 60 50 40 30 20 10 0

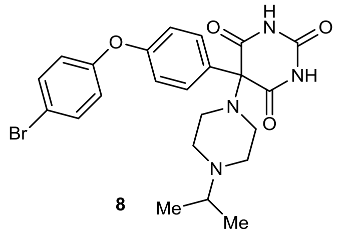

Br
O
H
N
NH
O
N
N
Me
Me

8

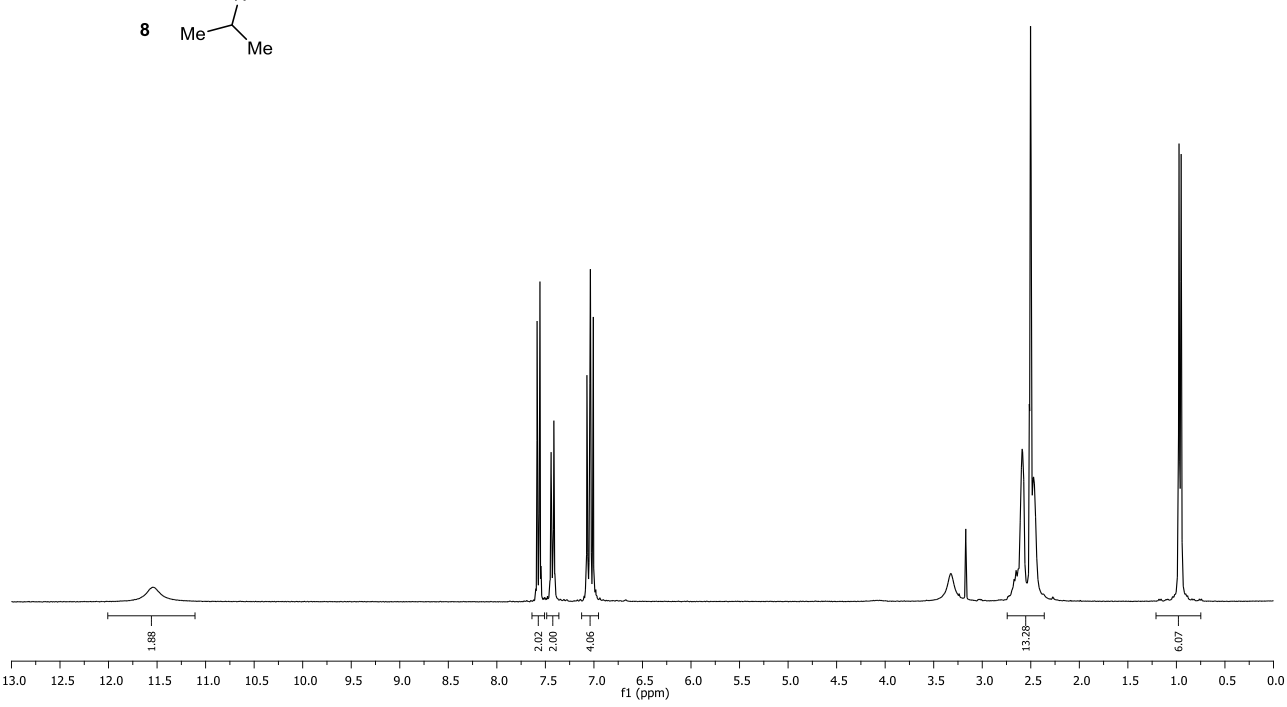

1.88
2.02
2.00
4.06
13.28
6.07
f1 (ppm)

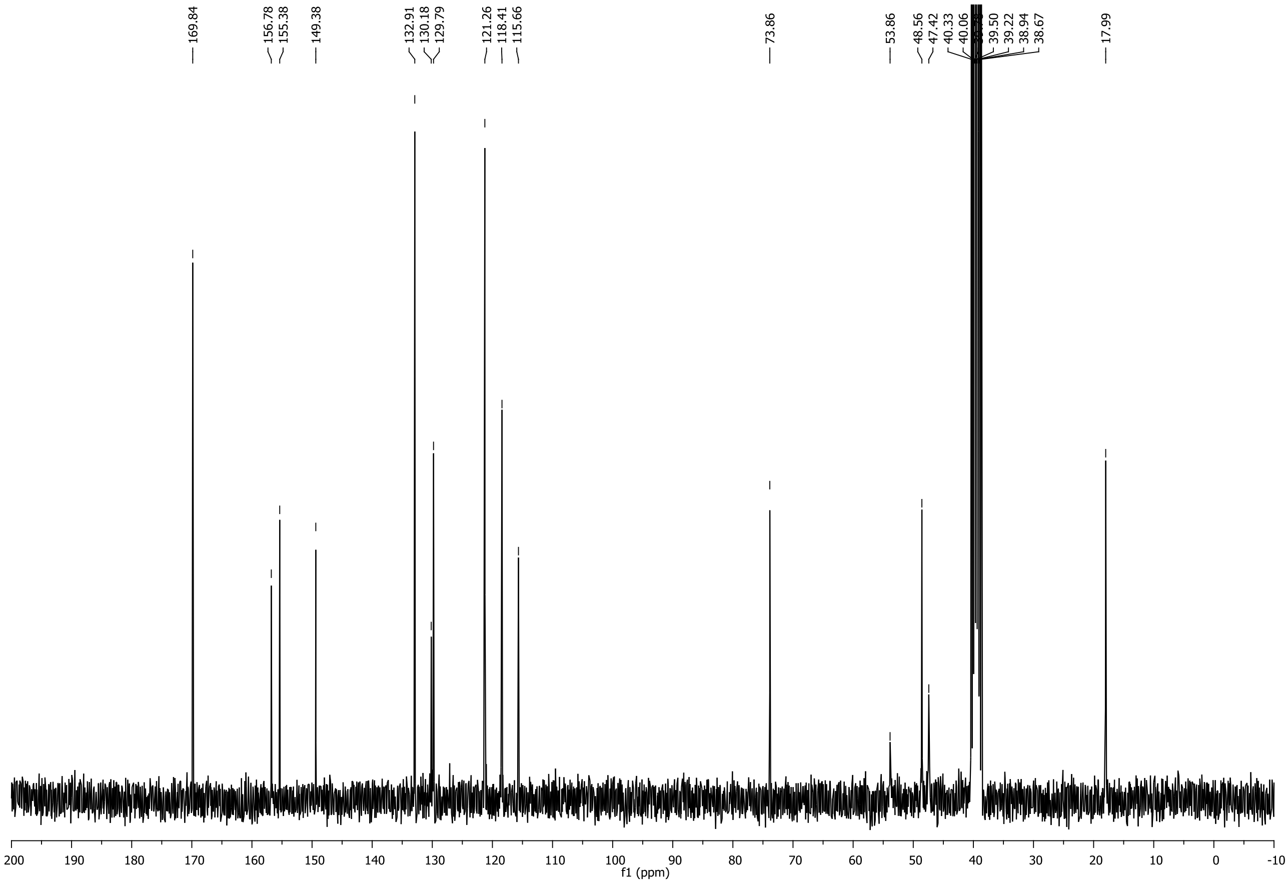

169.84
156.78
155.38
149.38
132.91
130.18
129.79
121.26
118.41
115.66
73.86
53.86
48.56
47.42
40.33
40.06
39.50
39.22
38.94
38.67
17.99
f1 (ppm)

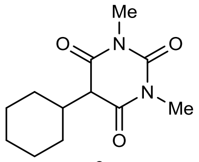
Me
O
N
O
N
Me
O

9

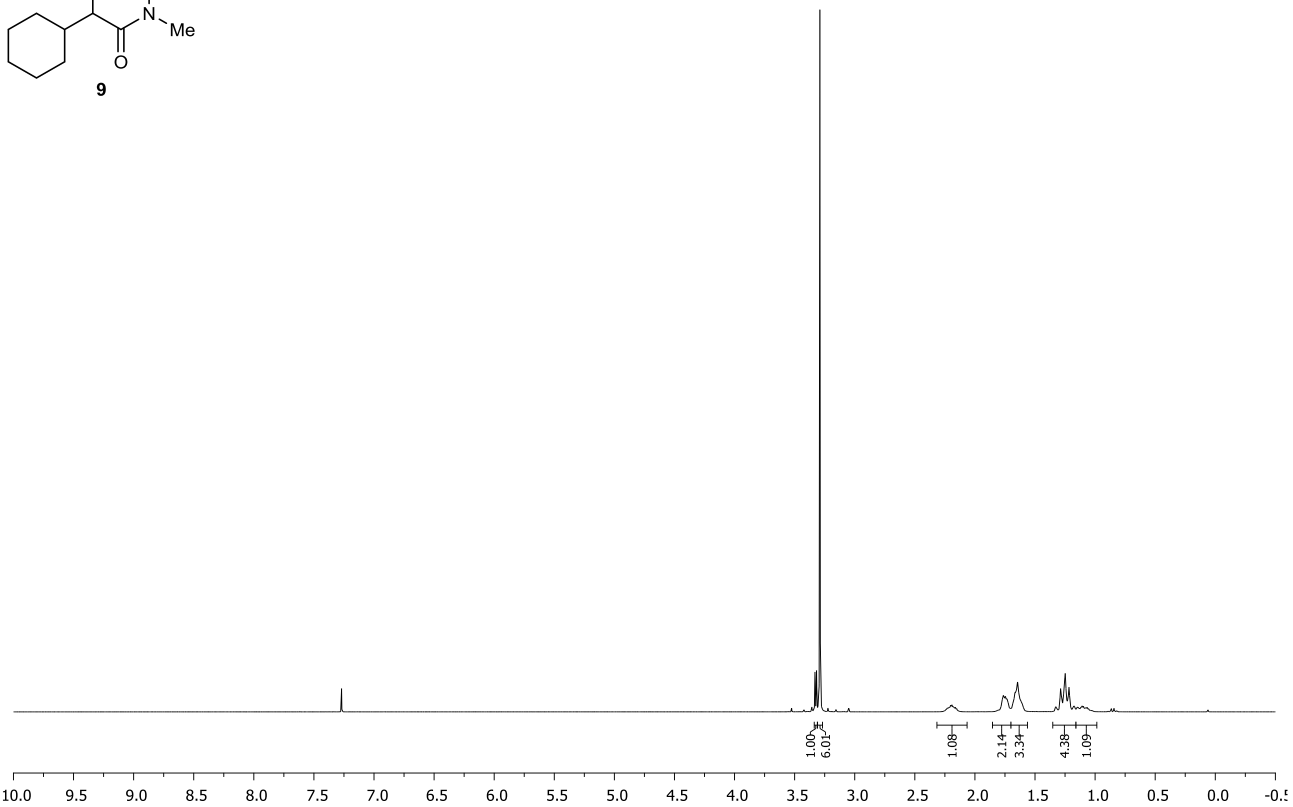
1.00
6.01
1.08
2.14
3.34
4.38
1.09
10.0 9.5 9.0 8.5 8.0 7.5 7.0 6.5 6.0 5.5 5.0 4.5 4.0 3.5 3.0 2.5 2.0 1.5 1.0 0.5 0.0

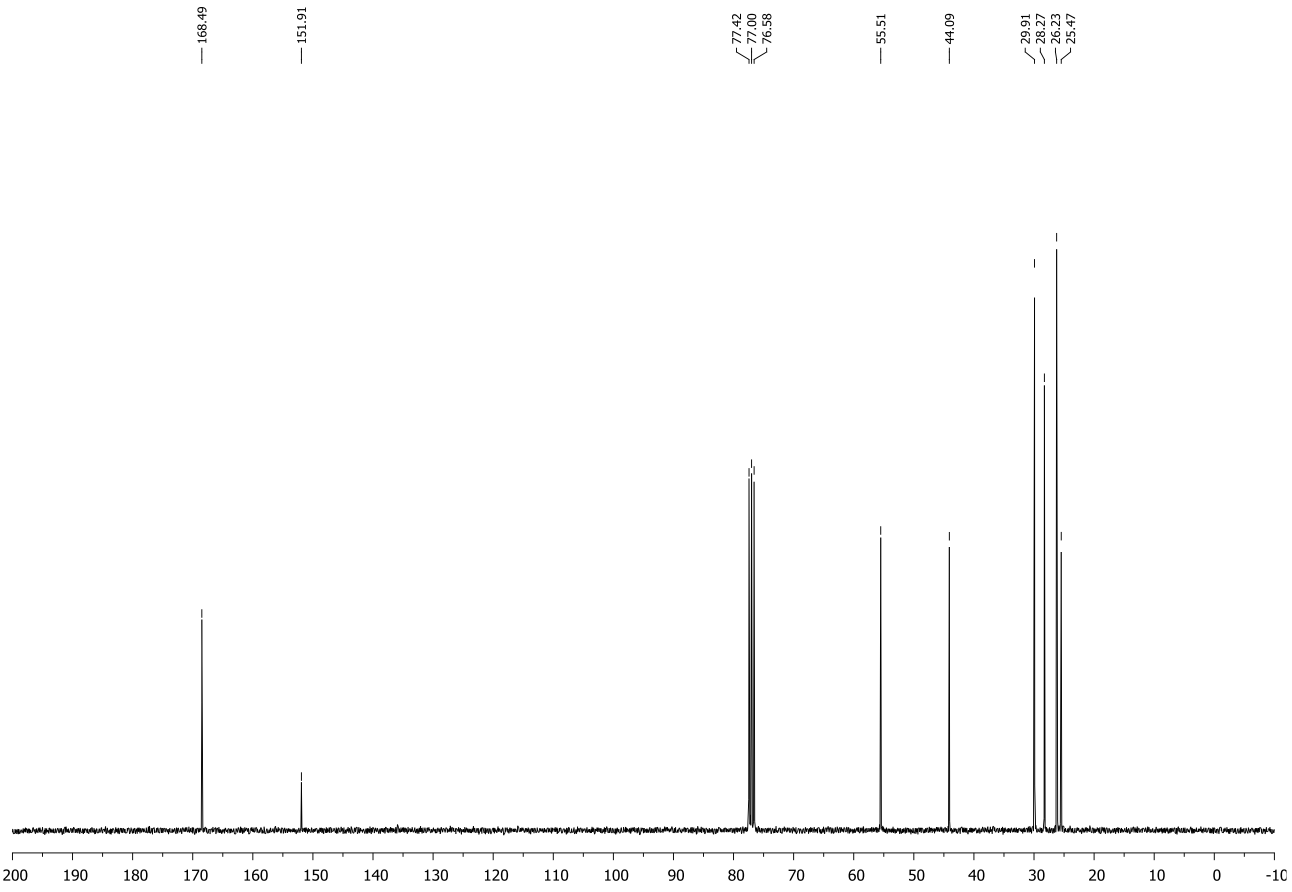
168.49
151.91
77.42
77.00
76.58
55.51
44.09
29.91
28.27
26.23
25.47
200 190 180 170 160 150 140 130 120 110 100 90 80 70 60 50 40 30 20 10 0 -10
